# Effect of Thermal Processing on the Metabolic Components of Black Beans on Ultra-High-Performance Liquid Chromatography Coupled with High-Field Quadrupole-Orbitrap High-Resolution Mass Spectrometry

**DOI:** 10.3390/molecules27123919

**Published:** 2022-06-18

**Authors:** Yuchao Feng, Xia Fan, Shu Zhang, Miao Yu, Tong Wu, Ying Liang, Changyuan Wang, Hongzhi Yang

**Affiliations:** 1 College of Food, Heilongjiang Bayi Agricultural University, Xinfeng Lu 5, Daqing 163319, China; fengyuchao0321@126.com (Y.F.); zshu996@163.com (S.Z.); fantayu_1997@163.com (M.Y.); wutong@byau.edu.cn (T.W.); liangying-64@163.com (Y.L.); 2 Institute of Quality Standards and Testing Technology for Agricultural Product, Chinese Academy of Agricultural Science, Beijing 100081, China; fanxia@caas.cn; 3 Chinese National Engineering Research Center, Daqing 163319, China

**Keywords:** metabolic components, hot working, UHPLC-QE-MS, black beans

## Abstract

An ultra-high-performance liquid chromatography coupled with high-field quadrupole-orbitrap mass spectrometry (UHPLC-QE-MS) histological platform was used to analyze the effects of two thermal processing methods (cooking and steaming) on the nutritional metabolic components of black beans. Black beans had the most amino acids, followed by lipids and polyphenols, and more sugars. Multivariate statistical analysis indicated that heat processing significantly affected the metabolic component content in black beans, with effects varying among different components. Polyphenols, especially flavonoids and isoflavones, were highly susceptible. A total of 197 and 210 differential metabolites were identified in both raw black beans and cooked and steamed black beans, respectively. Cooking reduced the cumulative content of amino acids, lipids, polyphenols, sugars, and nucleosides, whereas steaming reduced amino acid and lipid content, slightly increased polyphenol content, and significantly increased sugar and nucleoside content. Our results indicated that metabolic components were better retained during steaming than cooking. Heat treatment had the greatest impact on amino acids, followed by polyphenols, fatty acids, sugars, and vitamins, indicating that cooking promotes the transformation of most substances and the synthesis of a few. The results of this study provide a basis for further research and development of nutritional products using black beans.

## 1. Introduction

In recent years, global dietary preferences have gradually shifted toward increasing demand for plant-based foods, a trend that favors the expansion of the role of legumes in sustainable food systems, as they are rich in nutrients; are an excellent source of protein, vitamins, minerals, and antioxidants; and are low in fat [1,2,3]. Legumes also have a range of anti-diabetic, anti-obesity, anti-inflammatory, anti-mutagenic, and anti-cancer properties, and can lower cholesterol and blood pressure; moreover, they confer beneficial intestinal flora-regulating effects [4,5,6,7,8,9,10]. Despite these benefits, the consumption of legumes remains limited in many parts of the world, most likely due to the time required for processing. Heat treatment is a fundamental part of bean preparation which has a large influence on palatability and nutrient digestibility [11,12], and can also improve sensory attributes, such as aroma, taste, and texture [13]. However, due to the lack of standardized protocols or guidelines for cooking beans, the choice of processing method is usually based on affordability and convenience and not on maximizing the retention of nutritional properties. That food nutritional value is highly dependent on the form of processing has been well documented, and research has shown that several heat-insensitive anti-nutritional factors, such as urease and trypsin inhibitory factors in beans, can be inactivated during heat treatment. Thus, heat treatment can improve not only the nutritional value of beans [14], but also the digestibility and absorption of the micronutrients they contain.

Black beans (also known as black soybeans), a traditional food legume in China, are rich in nutrients and contain numerous active antioxidant elements, including polyphenols, phytosterols, vitamins, minerals, and trace elements [15], as well as having high levels of protein [16,17]. In addition to providing nutrients, the components of food items also influence physiological activities. Black beans have greater nutritional value and contain more active antioxidant substances than other, light-colored legumes [18], and studies have shown that black beans possess a variety of functional properties that are closely related to this high degree of antioxidant activity. For example, phytosterols and phenolic compounds in the coat of black beans have the potential to lower cholesterol levels in the gut microbiota by inhibiting the micelle solubility of cholesterol and reducing adipogenic gene expression [19,20,21], and polyphenolic substances in black beans can exert prebiotic and antibacterial effects on the pathogenic intestinal microbiota by altering the enzymatic activity of beneficial bacteria [22]. Furthermore, anthocyanins in the coating of black beans inhibit the production of pro-inflammatory cytokines and prevent beta-cell dysfunction, and thus have anti-inflammatory and anti-diabetic properties [23,24]. Research on rats with spontaneous hypertension has revealed that black beans have beneficial effects on the vascular compliance of small arteries, as well as potential for the nourishment, diuretics, and detoxification of blood [25,26].

Cooking and steaming are two of the most common approaches for preparing black beans. Because of the heat-resistant anti-nutritional factors inherent in these legumes, the application of heat is beneficial for improving their nutritional value [14]. However, nutrient leaching resulting from cooking can degrade vitamins, amino acids, and other compounds and/or thermally induced cross-linking reactions, and produce insoluble complexes which limit the digestibility of relevant nutrients [27]; thus, thermal processing can also have detrimental impacts on the nutritional maintenance of processed beans [28]. It is, therefore, crucial that the effects of different heat treatment processes on the nutrient composition of black beans be clarified in order to identify approaches that ensure maximal nutritional retention.

In this study, we treated black beans via atmospheric pressure cooking and steaming, and used an QE_XACTIVE_ HF omics platform to compare the effects of the two thermal treatments on bean nutrient quantity and content, and identified the pathways underlying changes in individual nutrient components for each treatment.

## 2. Results

### 2.1. Qualitative Results

Raw, cooked, and steamed black beans were characterized as having 827, 828, and 835 metabolic fractions in the positive and negative ion binding scan modes, respectively. Metabolic components included lipids, amino acids, sugars, polyphenols, alkaloids, terpenes, nucleosides/nucleotides, organic acids, ketones, aldehydes, acids, esters, alcohols, other alkenes, salts, pyrroles, pyrazines, and alkanes (with each group also containing derivatives). A total of 889 components were identified in the 3 kinds black bean samples, the majority of which were identified in positive ion mode. Further details regarding the metabolic components are provided in Table S1.

**Figure 1:**
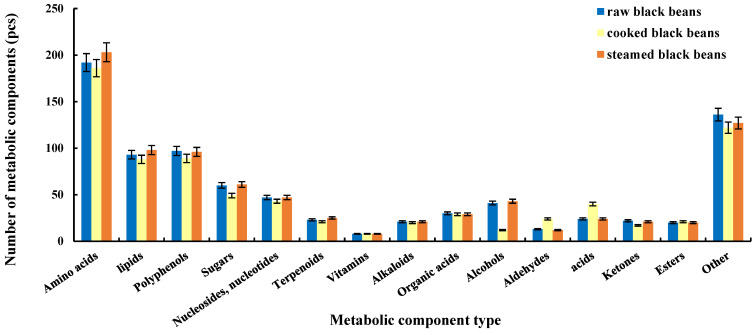
Comparison of the number of metabolic components in raw, steamed, and cooked black beans.

As shown in Figure 1, amino acids were the most common type of metabolic compound in black beans, followed by lipids and polyphenols. Thus, proteins, lipids, and polyphenols were more abundant than were other metabolic components, although sugars also accounted for a large proportion. In addition, raw black beans had the same type and similar amounts of metabolic components as the heat-processed black beans, which can be attributed to the nature of the metabolic components and processing methods. Common heat-processing methods, including steaming and cooking, typically have little effect on proteins, fats, sugars, and other substances, and although reductions in polyphenols and vitamins may occur, seldom is there a complete loss of these elements. In addition, the high sensitivity of the platform increases the likelihood that a given component will be detected even if only trace amounts remain following processing, which may account for why we observed only small changes in the number of metabolic components after heat treatment. Steaming, cooking, and other processing methods involve the use of water, high temperatures, and other factors that can lead to nutrient leaching or the degradation and cross-linking of some components [28]. These changes can greatly impact nutritional components, and thus alterations in their contents may be more notable. 

### 2.2. Orthogonal Projections to Latent Structures-Discriminant Analysis (OPLS-DA)

Metabolomic data based on the UHPLC-QE-MS platform are characterized by high dimensionality and small sample sizes. The variables include not only differential variables related to classification variables but also a large number of non-differential variables that may be correlated with one another. Therefore, dimensionality reduction using multivariate statistical analysis is more conducive to the visualization and subsequent analysis of the results.

**Figure 2:**
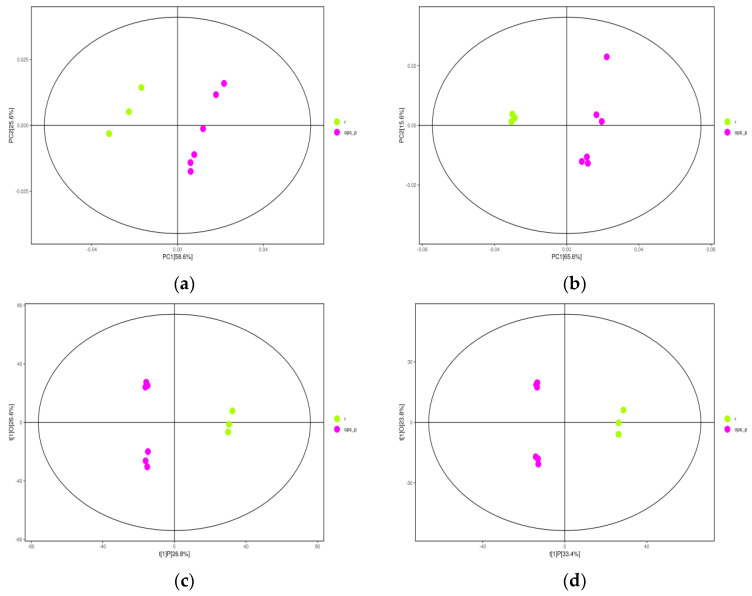
Dispersion point diagrams of the positive ion PCA and OPLS−DA of raw, steamed, and cooked black beans. (**a**) Dispersion point diagram of PCA in positive ion mode. (**b**) Dispersion point diagram of PCA in negative ion mode. (**c**) Dispersion point diagram of OPLS−DA in positive ion mode. (**d**) Dispersion point diagram of OPLS−DA in negative ion mode.

Principal component analysis (PCA) was used to process the sample data of raw, cooked, and steamed black beans. PCA scatter score plots under the positive and negative ion scanning modes are shown in Figure 2a,b (R^2^X = 0.938 and Q^2^ = 0.882 in the positive ion mode, R^2^X = 0.881 and Q^2^ = 0.814 in the negative ion mode). All model parameters were close to one, indicating that the model had high reliability and no overfitting, and all samples were within 95% confidence intervals. Significant differences were detected between raw black beans and the two types of heat-processed black beans, as demonstrated by their locations on the left and right sides of the confidence interval, respectively. However, no significant difference was found between steamed and cooked beans; moreover, aggregation within the sample group was poor, and the sample points were scattered. In contrast, there was a marked discrimination effect of the negative ion mode. 

OPLS-DA was then used to filter out the orthogonal variables unrelated to classification variables in the metabolic components, and the non-orthogonal variables and orthogonal variables were analyzed. Scatter scores of OPLS-DA in the positive and negative ion scanning modes are shown in Figure 2c,d (R^2^X = 0.535, R^2^Y = 0.999, and Q^2^ = 0.902 in the positive ion mode; R^2^X = 0.572, R^2^Y = 0.998, and Q^2^ = 0.94 in the negative ion mode). Both R^2^Y and Q^2^ were close to one, indicating that the model had high reliability with no overfitting. 

As shown in the OPLS-DA score chart, aggregation in the three sample groups was good, and significant differences were found between the raw, steamed, and cooked black beans, indicating that heat processing greatly affected the metabolic components of black beans. Although steamed and cooked black beans were located on the same side of the confidence interval, they occurred in the upper and lower quadrants, respectively. These results indicate that different heat-treatment methods have variable effects on the metabolic components of black beans.

### 2.3. Screening of Differential Metabolic Components of Black Beans during Heat Processing

**Figure 3:**
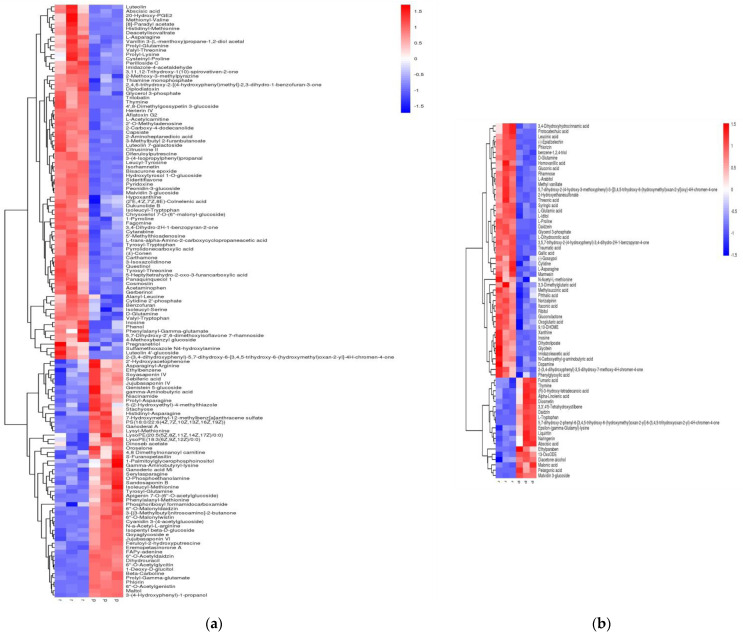
Heat map of hierarchical cluster analysis of differential metabolites in raw and cooked black beans. (**a**) Positive ion mode. (**b**) Negative ion mode.

Raw, cooked, and steamed black beans were compared and analyzed in pairs to screen for differential metabolic components. Differential metabolites are defined as substances present in the control group that differ greatly in content. The differential metabolites found in black beans are listed in Tables S2−S4, and the classification and quantities of the differential metabolites are shown in Table 1. Figure 3 shows the hierarchical cluster analysis heat map of the differential metabolites screened from raw and cooked black beans; red represents differential metabolites with high content and blue represents differential metabolites with low content. From this figure, it can be clearly seen that the content of the various metabolites differed significantly between raw and cooked black beans. Compared to cooked black beans, 197 different metabolites were screened under positive and negative ion modes, among which the content of 125 metabolites were found to be significantly reduced after cooking, and those of 72 metabolites increased after cooking. The results suggest that the use of large amounts of water and high temperatures to cook beans had a significant effect on the content of metabolic components. 

Analysis of the number of differential metabolites revealed that polyphenols, amino acids, and fatty acids were the components most affected by cooking, composing half of the total number of differential metabolites, among which the number of polyphenols accounted for the largest change. Additionally, sugar and nucleoside contents were also changed to a slightly higher degree than the other metabolic components. In terms of changes in cumulative content, the decrease in amino acid content was 9.67-fold that of the increase in amino acids, the decrease in lipid content was 4.73-fold that of the increase in lipids, and the decrease in polyphenol content was 1.06-fold higher than the increase in polyphenols. Flavonoids were the most varied polyphenols, followed by isoflavones. Sugar content cumulatively decreased by 1.05-fold of the cumulative increase, whereas nucleoside content cumulatively decreased by 4.12-fold compared to the cumulative increase.

To some extent, variations in multiple differential metabolites can also reflect the impact thermal processing has on metabolic components. Changes in amino acid content ranged from 0.2152–7.6190-fold, whereas changes in lipid content varied between 0.4716–8.6530-fold for most of the increased and decreased contents, respectively. The variation in polyphenol content was between 0.0961–11.3197-fold, and polyphenols were more susceptible to thermal processing. 

Changes in methothianyl valine (50.8817-fold), L-asparagine (35.1139-fold), deacetylisopentanoic acid (214.8903-fold), glycerophosphite (990.1842-fold), luteolin 7-galactoside (74.6299-fold), macrocoside (49.0878-fold), trilobin (32.2253-fold), luteolin 4′-glucoside (19.1610-fold), and luteolin (15.8823-fold), among others, were particularly significant following cooking (the degrees of the content changes for these substances after cooking are shown in parentheses).

A hierarchical cluster analysis heat map of differential metabolites clearly shows that their contents differed significantly between raw and steamed black beans. A total of 210 metabolites were identified in steamed black beans under positive and negative ion modes, of which 96 significantly decreased after steaming and 114 increased after steaming, an indication that metabolites were less affected by the smaller amount of water and lower temperature used for steaming. 

The quantification of differential metabolites indicated that polyphenols, amino acids, and lipids were the components most impacted by steaming, followed by sugars and nucleosides. These five substances accounted for more than half of the total number of differential metabolites, among which polyphenols were the most abundant. The cumulative decrease in amino acid content was 3.38-fold that of the cumulative increase, the cumulative decrease in lipid content was 5.14-fold that of the cumulative increase, and the cumulative decrease in polyphenol content was 0.90-fold that of the cumulative increase. Overall, polyphenol content increased slightly, whereas the decrease in cumulative content was attributed to flavonoids. Half of the increase in cumulative content was due to flavonoids, and half was due to isoflavones. The cumulative increase in sugar content was 3.02-fold of the cumulative decrease in sugar content, and the cumulative increase in nucleoside content was 17.86-fold of the cumulative decrease in nucleoside content. In terms of variation of change among the differential metabolites, for amino acids, the increase and decrease in content ranged from 0.0086–4.3727-fold, whereas that of lipids ranged from 0.0091–4.8169-fold and that of polyphenols ranged from 0.0003–5.2338-fold. That the range of changes were similar is evidence that steaming had essentially the same effect on the three metabolites. Changes in the contents of isopentanoic acid (273.0420-fold) and 2-(3,4-dihydroxyphenyl)-5,7-dihydroxy-6-[3,4,5-trihydroxy-6-(hydroxymethyl)oxan-2-yl]-4H-chromen-4-one (10.0405-fold) following steaming were particularly significant.

**Figure 4:**
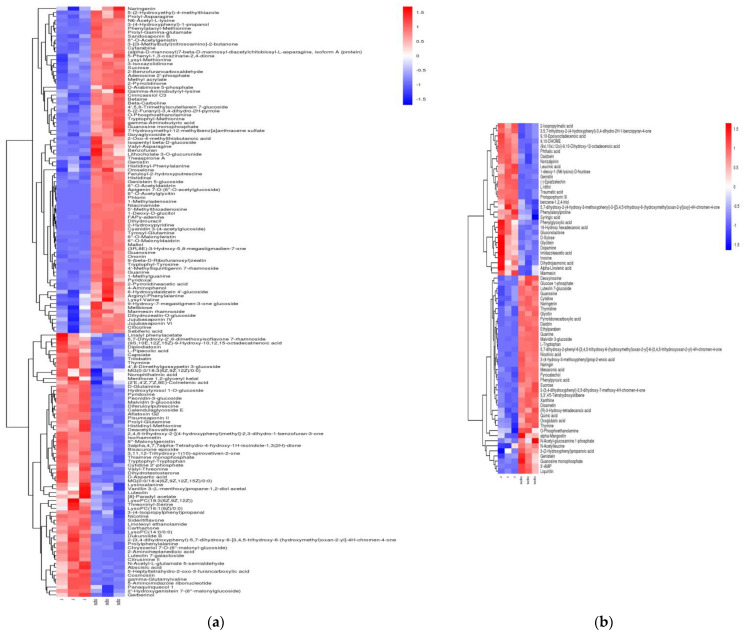
Heat map of hierarchical cluster analysis of differential metabolites of raw and steamed black beans. (**a**) Positive ion mode. (**b**) Negative ion mode.

Steaming and cooking are two of the most commonly used thermal processing methods for treating legumes. Figure 3 and Figure 4 show that although both heat treatments had significant impacts on the metabolic components of black beans, effects differed depending on treatment. During steaming and cooking, polyphenols were the most susceptible to alteration, among which flavonoids were the most affected, followed by isoflavones. During cooking, more metabolic components decreased in content than during steaming; the cumulative contents of amino acids, lipids, polyphenols, sugars, and nucleosides all decreased, and amino acid contents were reduced. In terms of quantity, more substances increased in content after steaming than after cooking; moreover, although amino acid and lipid contents decreased, polyphenol content increased slightly, and sugar and nucleoside contents increased significantly. Compared with cooking, lipid content was lost to a greater extent. These results suggest that cooking black beans by steaming leads to the better retention of metabolic components than cooking. 

A comparison of raw beans to steamed and cooked beans showed that the contents of 114 metabolic components were significantly altered by steaming and cooking, including 12 amino acids, 8 sugars, 10 lipids, 36 polyphenols (including 22 flavonoids), 9 nucleosides, and 39 alcohols, esters, terpenes, acids, alkaloids, aldehydes, and other substances. Thermal processing had the greatest effect on polyphenols, especially flavonoids, followed by amino acids, lipids, nucleosides, and sugars; the contents of alcohols, esters, acids, aldehydes, and other substances were also higher, indicating that these substances were transformed or degraded during heating.

**Figure 5:**
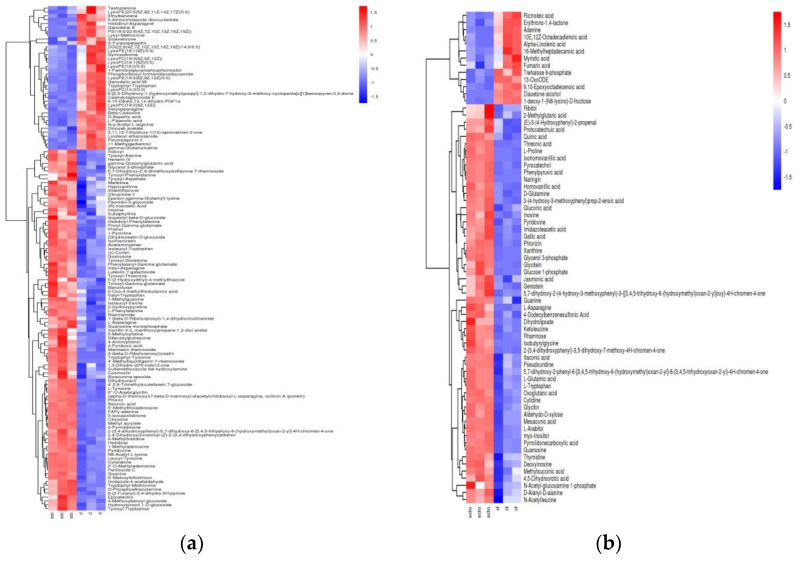
Heat map of hierarchical cluster analysis of differential metabolites of steamed and cooked black beans. (**a**) Positive ion mode. (**b**) Negative ion mode.

Figure 5 depicts a heat map of the hierarchical cluster analysis of the differential metabolites in black beans following steaming and cooking. As can be seen in the figure, differential metabolites were more abundant in steamed than cooked black beans. A total of 193 differential metabolites were identified in processed black beans in positive and negative ion modes. The content of 143 metabolic components (primarily consisting of amino acids, sugars, polyphenols, and nucleosides) was higher in steamed than in cooked black beans. Contents of 50 metabolic components were lower in steamed beans than in cooked beans, with lipids accounting for a large proportion of these components. In terms of changes in the contents of different components, the cumulative contents of different amino acids, sugars, polyphenolsins, and nucleosides in steamed black beans were 1.57-fold, 1.90-fold, 2.21-fold, and 3.20-fold higher than those in cooked black beans, respectively; the cumulative differential fatty acid content of cooked black beans was 2.03-fold higher than that of steamed beans; and levels of L-asparagine (35.5763-fold), tryptophan-tyrosine (42.0156-fold), glycerophosphate (964.1508-fold), 2-(3,4-dihydroxyphenyl)-3,5-dihydroxy-7-methoxy-4H-chromen-4-one (17.93-fold), naringin (2314.0121-fold), and 5-methylcytidine (38.4834-fold) were significantly higher in steamed than in cooked black beans. Comparisons between steamed and cooked beans are similar to those between raw and cooked beans and raw and steamed beans—further evidence that, for black beans, processing by steaming is more conducive to nutrient retention.

### 2.4. Pathway Analysis

Metabolic processes continue after harvesting but end after thermal processing, preventing the use of metabolic pathway analysis to investigate component pathways. Nonetheless, pathway analysis comparing beans prior to and after heating can provide insights into the transformation of metabolic components during the cooking process.

KEGG (Kyoto Encyclopedia of Genes and Genomes) annotation analysis and a comprehensive analysis (including enrichment and topological analyses) were performed to examine the pathways of the differential metabolic components, with key pathways (i.e., those with the highest differences in correlation) undergoing further investigation. The metabolic pathways associated with cooking and steaming are listed in Table 2 and Table 3, respectively. 

A total of 33 key pathways were identified for cooked black beans, of which 13 were related to amino acids, 2 were related to amino acid synthesis, and 11 were related to amino acid metabolism, indicating that although amino acids were transformed at high rates, fewer were synthesized after cooking. Seven pathways related to fatty acids were also identified, consisting of one fatty acid synthesis pathway and six fatty acid metabolic pathways. The synthesis pathway involved an unsaturated fatty acid, and the metabolic pathway primarily involved saturated fatty acids. Three pathways were related to polyphenols, all of which were synthetic pathways involving flavonoids, isoflavones, astragalus, and phenols. Additionally, three pathways associated with synthetic and metabolic glucose metabolism, three nucleoside metabolism and synthesis pathways, two alkaloid metabolic pathways, and two vitamin metabolic pathways were identified.

A total of 33 key pathways were identified in steamed black beans, of which 12 were related to pathways involving amino acids, 3 were involved in the synthesis of amino acids, and 9 consisted of amino acid metabolism pathways, suggesting that amino acid transformation is a major part of the steaming process. Six pathways involving the transformation, synthesis, and metabolism of glucose were identified, as were four pathways involving fatty acids, among which was one unsaturated fatty acid synthesis pathway and three saturated fatty acid metabolic pathways. Three flavonoid synthesis, three vitamin metabolism, three nucleotide metabolism and synthesis, and two alkaloid synthesis pathways were also detected.

Of these pathways, 25 were common to both heat-processing methods, thus representing the general effect heating has on metabolic transformations in black beans. Of these, amino acids were the most affected, followed by polyphenols, fatty acids, sugars, and vitamins. This indicates that thermal processing can promote the conversion of a majority of substances and the synthesis of a smaller number; more simply, cooking and steaming reduces the content of most components but increases the content of a few.

## 3. Discussion

Cooking promotes complex interactions among food components, affecting their nutritional, functional, and sensory (e.g., aroma, flavor) properties. At the same time, heat treatment can also trigger the production of substances that detract from these properties, or that may even be detrimental human health. Proteins, sugars, and lipids are the main components of food. In black beans, the results of our analysis suggest that the number and content of amino acids, lipids, and sugars were the most affected by heat processing. Exposure to heat generally increases the rate of oxidation of proteins and lipids; during cooking, caramelization, the Maillard reaction, and both lipid and protein oxidation can occur among proteins, lipids, and sugars, which may, in turn, trigger the transformation of other substances. As such, differences in the content and quantity of components after heat treatment can be attributed to interactions among various substances, such as the addition, rearrangement, and polymerization of carbonyl (reducing sugars, ketones, phenols, and aldehydes) and amino (proteins, peptides, amino acids, and amines) compounds.

Although previous studies have evaluated changes in legume components during thermal processing, most have focused on changes in the macroscopic content of specific substances. From a nutritional perspective, heat treatment can reduce protein content [29], as cooking can promote structural alterations in proteins, leading to protein denaturation, aggregation, and chemical modification [30]. For instance, Dong et al. reported that boiling reduces amino acid content in seeds [31,32,33], which we also found for steamed and cooked black beans; however, few studies have explored the reasons why heating reduces amino acid content. Of the handful of studies that have attempted to address this question, Chopra et al., for one, postulated that the general reduction in amino acids caused by heat treatment is due to amino acid dissolution and leaching [34], whereas Yagoub et al. [35] similarly suggested that transamination and deamination reactions may result in changes in amino acid content.

Exposure to high temperatures triggers lipid oxidation, leading to the formation of short-chain monomers and carbonyl compounds. For example, highly reactive microaldehydes and ketones can react with proteins, amino acids, and other metabolic components [36]. In addition to inducing their own changes, lipids can also be involved in the degradation of other nutrients, such as through oxidation and degradation of amino acids [37,38,39]. Studies have shown that lipid oxidation and acrylamide, carboxymethyl lysine, and furan contents produced during the thermal processing of food have important effects [40,41,42]. As demonstrated by our results, in addition to the five major categories of metabolic components for which significant changes in content occurred, elevated levels of acids, ketones, aldehydes, alcohols, and furans were also observed, providing further evidence that boiling and steaming transforms and degrades many substances. Fats and oils are also highly susceptible to hydrolysis reactions in the presence of high temperatures and moisture [43], the end products of which are glycerol and free fatty acids. Changes in the cumulative content of the differentially metabolized components indicate that cooking and steaming both caused significant reductions in lipid content, such that fatty acid synthesis occurred at a lower rate than did conversion and degradation.

Sugars can be caramelized during cooking and steaming, whereas polysaccharides can be hydrolyzed several times to produce monosaccharides, which can be formed into oxygen-heterocyclic and carboxyclic compounds with conjugated double bonds (e.g., furfural, furanone) through enolization processes (e.g., dehydration, dicarboxylic acid cracking, inverse aldehyde condensation, aldehyde alcohol condensation, free radical reactions). The thermal degradation of sugar also produces dicarbonyl compounds (e.g., methyl glyoxaldehydes, organic acids), which can be further condensed and polymerized to form various polymers. Black beans are rich in starch; cooking, steaming, and other thermal processes can gelatinize starch because of the increased utilization rate of water, from which sugars such as glucose, maltose, and maltooligosaccharide are readily generated. Previous research has demonstrated that cooking can reduce the starch content of black beans, indicating the occurrence of starch hydrolysis, which should increase sugar abundance. In this study, the overall decrease in the starch content of cooked black beans was 1.05-fold that of the cumulative increase, indicating that the decrease and increase in starch occurred at more or less similar rates. This may be due to two processes: first, although the overall sugar content increased, sugars also interact with other components, often converting them to something else; second, in addition to temperature, sugars are highly soluble in water, which is used in abundance during cooking. In contrast, sugar content increased by more than threefold relative to the cumulative decrease in steamed black beans, most likely because only a small amount of water is used during the steaming process and, thus, less sugar is dissolved. 

High temperatures can degrade or chemically oxidize unstable polyphenols to form quinones. The presence of water during steaming and cooking usually intensifies the degree of heat treatment [44]. Siah et al. [45] found that the phenol content of beans fell after cooking; likewise, Xu et al. [46], who compared polyphenol content in black beans after cooking and steaming, noted that both cooking methods significantly reduced the content of total polyphenols, flavonoids, condensed tannins, and single anthocyanins, but that steamed beans had a higher total polyphenol content than cooked beans. Although the results of our study are similar to those reported by Xu et al., it is worth noting that those authors used a macroscopic content determination approach. In a subsequent study, Xu et al. [47] used HPLC to conduct quantitative analyses of phenolic acids, anthocyanins, flavane-3-alcohols, flavonols, flavonoids, and other substances in different types of beans after thermal processing, the results of which suggested that, for phenol retention in legumes, steaming is superior to cooking; moreover, the authors found that, while all thermal processing methods reduced flavonoid content, conventional cooking had no effect on non-flavonoid contents. Here, we found that, among polyphenols, both cooking and steaming not only significantly reduced flavonoid and isoflavone contents but also that of lignin, phenylpropanoid, astragalus, and coumarin as well.

## 4. Materials and Methods

### 4.1. Plant Materials

Black beans (full grain, uniform color, and no pest damage) were purchased from Mudanjiang City, Heilongjiang Province, China, in 2021.

### 4.2. Reagents and Instruments

Methanol and acetonitrile were purchased from CNW Technologies, acetic acid from Fisher Chemical, and ammonium acetate from Sigma-Aldrich (Shanghai, China). All reagents were of LC-MS grade. Ultra-high-performance liquid chromatography was performed using Q Exactive HFX High-Resolution Mass Spectrometry (Heraeus Fresco Model 17 Centrifuge; Thermo Fisher Scientific, Waltham, MA, USA). A scale (model BSA124S-CW) was purchased from Sartorius. A Ps-60al ultrasonic instrument was purchased from Shenzhen Redbond Electronics Co., Ltd. A rice cooker (Mb-wfs4029 Midea) was purchased from Midea Life Electric Appliance Manufacturing Co., Ltd. (Foshan, China).

### 4.3. Sample Processing

#### 4.3.1. Processing of Raw Black Beans

Black beans were washed with distilled water and immediately dried in an oven at 40 °C to obtain a moisture content of 10–11%. The dried samples were crushed in a grinder, passed through a 70-mesh sieve, and stored at −20 °C until further use.

#### 4.3.2. Cooking Method

Black beans were washed with deionized water and dried following the procedures described in Xu et al. [47], weighed (50 g), and soaked at room temperature to ensure a hydration rate of 50%. The soaked samples were placed in an electric rice cooker at a solid–liquid ratio of 1:500. After cooking for 40 min, beans were removed and dried at low temperature in an oven until the samples maintained a constant weight, followed by crushing in a grinder, and stored at −20 °C until further use.

#### 4.3.3. Steaming Method

Black beans were washed with deionized water and air-dried following the procedures described in Xu et al. [47], weighed (50 g), and soaked at room temperature to ensure a hydration rate of 50%; 500 mL of deionized water was added to the rice cooker and placed into the steamer frame. Beans were laid flat and removed after steaming for 60 min (optimal steaming time was determined pre-experimentally). The beans were then dried in an oven at low temperature until the sample remained at a constant weight, followed by grinding and storage at −20 °C until further use.

### 4.4. QE_XACTIVE_ HF Detection

#### 4.4.1. Metabolite Extraction

Bean powder (5 g) was added to 20 mL of extraction solution (acetonitrile containing isotopically-labeled internal standard mixture) and extracted using ultrasound for 30 min. The solution was then vortexed and mixed using an oscillator for 30 min, then centrifuged at 12,000 rpm for 15 min at 4 °C. The resulting supernatant was filtered through a 0.25 μm membrane and transferred into a fresh glass vial for further analysis. A quality control (QC) sample was prepared by mixing an equal aliquot of supernatant from all samples.

#### 4.4.2. LC-MS/MS Analysis

LC-MS/MS analysis was performed using a UHPLC system (Vanquish, Thermo Fisher Scientific) with a UPLC HSS T3 column (2.1 mm × 100 mm, 1.8 μm) coupled to a Q Exactive HFX mass spectrometer (Orbitrap MS, Thermo). The mobile phase consisted of 5 mM ammonium acetate and 5 mM acetic acid in water (A) and acetonitrile (B). The auto-sampler temperature was 4 °C and injection volume was 3 μL. 

The QE HFX mass spectrometer was used because of its capacity to acquire MS/MS spectra in information-dependent acquisition (IDA) mode in the control of the acquisition software (Xcalibur, Thermo). In this mode, the acquisition software continuously evaluated the full-scan MS spectrum. The ESI source conditions were set as follows: sheath gas flow rate, 30 Arb; Aux gas flow rate, 10 Arb; capillary temperature, 350 °C; full MS resolution of 60,000, MS/MS resolution of 7500, collision energy 10/30/60 in NCE mode; spray voltage of 4.0 kV (positive) or −3.8 kV (negative).

### 4.5. Data Analysis

#### 4.5.1. Data Preprocessing and Annotation

Raw data were converted to mzXML format using ProteoWizard and processed with an in-house program developed using R and based on XCMS, for peak detection, extraction, alignment, and integration. An in-house MS2 database (BiotreeDB) was used for metabolite annotation. The cut-off for annotation was set at 0.3.

#### 4.5.2. Principal Component Analysis (PCA)

SIMCA software (v16.0.2, Sartorius Stedim Data Analytics AB, Umeå, Sweden) was used for centralized (CTR) data formatting, followed by automatic modeling analysis.

#### 4.5.3. Orthogonal Partial Least Squares-Discriminant Analysis (OPLS-DA)

SIMCA software was used for OPLS-DA modeling analysis, which was performed for the first principal component, and sevenfold cross validation was used to verify the quality of the model. Subsequently, R2Y (interpretability of the model to the classification variable Y) and Q2 (predictability of the model), obtained after cross-validation, were used to evaluate model validity.

#### 4.5.4. Screening of Differential Metabolites

The *p*-value of the Student’s *t*-test was set to less than 0.05, and the variable importance in the projection (VIP) of the first principal component of the OPLS-DA model was set to greater than 1.

#### 4.5.5. Hierarchical Cluster Analysis of Differential Metabolites

Values for the differential metabolites obtained by comparison of each group were calculated using the Euclidean distance matrix, and differential metabolites were clustered using the complete linkage method and displayed as a thermal diagram.

#### 4.5.6. KEGG Annotation and Metabolic Pathway Analysis of Differential Metabolites

The Kyoto Encyclopedia of Genes and Genomes (KEGG) annotation of differential metabolites was performed using the KEGG pathway database (http://www.kegg.jp/kegg/pathway.html, accessed on 10 March 2022). Authoritative metabolite databases, such as KEGG and PubChem, were mapped using differential metabolites. After obtaining the matching information of differential metabolites, the pathway database of the corresponding species was searched, and the metabolic pathways were analyzed.

## 5. Conclusions

In this study, a UHPLC-QE-MS omics platform was used to investigate the effects of two thermal processing methods—cooking and steaming—on the metabolic components of black beans, specifically endogenous metabolites. The results showed that, although both heat treatments significantly affected the metabolic fractions, the extent of the effects varied greatly among fractions. Polyphenols, especially flavonoids and isoflavones, were the most susceptible to both processing methods. Cooking reduced the content of the metabolic components of black beans by a larger number of substances, and the accumulated content of amino acids, lipids, polyphenols, sugars, and nucleosides was reduced, with a complete loss of amino acids. Steaming increased the content of more substances, reduced the content of amino acids and lipids, slightly increased the content of polyphenols, and significantly increased the contents of sugars and nucleosides. These results suggest that steaming is more conducive to the retention of metabolic components in black beans than cooking. A comprehensive analysis of the differences between the metabolic components of cooked and steamed black beans revealed that thermal processing had the greatest effect on the conversion of metabolic components of black beans for amino acids, followed by polyphenols, fatty acids, sugars, and vitamins, an indication that thermal processing can promote the conversion of most substances of all types and the synthesis of a few (i.e., the content of most substances will be reduced, whereas the content of only a few will be increased).

Here, heat treatments were performed only under atmospheric pressure. As such, it will be necessary to evaluate other types of processing methods, such as dry heat, high-pressure treatment, and drying and freezing treatments, to comprehensively evaluate the impact of processing on the nutritional quality of beans. Processing conditions and the time needed for processing are key factors that limit the utilization of beans. Thus, optimizing these processing conditions is crucial for ensuring the safety and quality of legumes prepared by heat treatment. However, this process involves complex interactions between various factors, and the identification of optimal processing conditions is needed to improve bean palatability and quality, and to maximize nutrient retention. At present, relatively little research has focused on nutrient transformation in beans resulting from thermal processing; as such, clarifying the changes in nutritional components triggered by different processing methods has practical significance for the development of black-bean-based products and the expansion of bean popularity and consumption worldwide.

## Figures and Tables

**Table 1:** Comparison of differential metabolite quantities.

	Raw−Cooked			Raw−Steamed			Steamed−Cooked		
	Reduced Content	Increased Content	Total	Reduced Content	Increased Content	Total	High Content	Low Content	Total
Amino acids	21	13	34	15	17	32	34	9	43
Sugars	10	3	13	7	9	16	11	1	12
Lipids	12	11	23	19	6	25	9	20	29
Polyphenols	24	19	43	20	30	50	29	2	31
Nucleosides	8	3	11	5	16	21	18	2	20
Other	20	9	29	9	11	20	15	7	22
Aldehydes	3	3	6	2	2	4	2	0	2
Alkaloids	5	0	5	3	3	6	4	0	4
Acids	10	1	11	7	6	13	8	0	8
Terpenes	2	5	7	3	4	7	1	5	6
Ketones	2	2	4	2	4	6	1	1	2
Vitamins	1	0	1	1	0	1	2	0	2
Organic acids	0	3	3	0	2	2	4	1	5
Esters	6	1	7	3	1	4	1	1	2
Alcohols	0	0	0	0	3	3	4	1	5
Total	124	73	197	96	114	210	143	50	193

**Table 2:** Pathway analysis of different components of black beans following steaming.

	Pathway	Total	Hits	Raw p	−ln(p)	Holm Adjust	FDR	Impact	Hits Cpd
1	Glycerophospholipid metabolism	25	2	0.153	1.879	1	1	0.186	O-Phosphoethanolamine cpd:C00346; Glycerol 3-phosphate cpd:C00093;
2	Glycerolipid metabolism	13	1	0.309	1.173	1	1	0.079	Glycerol 3-phosphate cpd:C00093
3	Sphingolipid metabolism	13	1	0.309	1.173	1	1	0.333	O-Phosphoethanolamine cpd:C00346
4	alpha-Linolenic acid metabolism	23	1	0.562	0.577	1	1	0.160	Alpha-Linolenic acid cpd:C06427
5	Biosynthesis of unsaturated fatty acids	42	1	0.781	0.247	1	1	0.000	Alpha-Linolenic acid cpd:C06427
6	Citrate cycle (TCA cycle)	20	1	0.512	0.670	1	1	0.034	Fumaric acid cpd:C00122
7	Butanoate metabolism	18	1	0.475	0.744	1	1	0.000	L-Glutamic acid cpd:C00025
8	beta-Alanine metabolism	12	1	0.289	1.240	1	1	0.000	Dihydrouracil cpd:C00429
9	Histidine metabolism	16	2	0.366	1.004	1	1	0.000	Imidazole-4-acetaldehyde cpd:C05130; Imidazoleacetic acid cpd:C02835
10	Alanine, aspartate, and glutamate metabolism	22	3	0.039	3.255	1	1	0.339	L-Glutamic acid cpd:C00025; L-Asparagine cpd:C00152; Fumaric acid cpd:C00122
11	Tyrosine metabolism	18	2	0.128	2.054	1	1	0.045	Dopamine cpd:C03758; Fumaric acid cpd:C00122
12	Cysteine and methionine metabolism	34	1	0.623	0.473	1	1	0.048	5′-Methylthioadenosine cpd:C00170
13	Arginine and proline metabolism	38	3	0.144	1.938	1	1	0.227	L-Glutamic acid cpd:C00025; L-Proline cpd:C00148; Fumaric acid cpd:C00122
14	Glycine, serine, and threonine metabolism	30	1	0.660	0.415	1	1	0.000	L-Tryptophan cpd:C00078
15	Phenylalanine, tyrosine, and tryptophan biosynthesis	21	1	0.529	0.637	1	1	0.000	L-Tryptophan cpd:C00078
16	Tryptophan metabolism	27	1	0.621	0.476	1	1	0.171	L-Tryptophan cpd:C00078
17	Glutathione metabolism	26	1	0.607	0.499	1	1	0.078	L-Glutamic acid cpd:C00025
18	Nitrogen metabolism	15	1	0.415	0.879	1	1	0.000	L-Glutamic acid cpd:C00025
19	Aminoacyl-tRNA biosynthesis	67	4	0.203	1.597	1	1	0.000	L-Asparagine cpd:C00152; L-Tryptophan cpd:C00078; L-Proline cpd:C00148; L-Glutamic acid cpd:C00025
20	Porphyrin and chlorophyll metabolism	29	1	0.648	0.435	1	1	0.000	L-Glutamic acid cpd:C00025
21	Flavonoid biosynthesis	43	2	0.449	0.802	1	1	0.122	Naringenin cpd:C00509; (-)-Epiafzelechin cpd:C12128; Luteolin cpd:C01514
22	Stilbenoid, diarylheptanoid, and gingerol biosynthesis	10	1	0.300	1.203	1	1	0.000	3,3′,4′5-Tetrahydroxystilbene cpd:C05901
23	Flavone and flavonol biosynthesis	9	1	0.226	1.488	1	1	0.000	Luteolin cpd:C01514
24	Vitamin B6 metabolism	11	1	0.269	1.314	1	1	0.000	Pyridoxine cpd:C00314
25	Thiamine metabolism	11	2	0.036	3.334	1	1	0.471	5-(2-Hydroxyethyl)-4-methylthiazole cpd:C04294; Thiamine monophosphate cpd:C01081
26	Pantothenate and CoA biosynthesis	14	1	0.329	1.112	1	1	0.000	Dihydrouracil cpd:C00429
27	Pyrimidine metabolism	38	2	0.665	0.408	1	1	0.000	Dihydrouracil cpd:C00429; Cytidine cpd:C00475
28	Purine metabolism	61	4	0.241	1.424	1	1	0.050	Hypoxanthine cpd:C00262; Phosphoribosyl formamidocarboxamide cpd:C04734; Inosine cpd:C00294; Xanthine cpd:C00385
29	Isoquinoline alkaloid biosynthesis	6	1	0.192	1.648	1	1	0.500	Dopamine cpd:C03758
30	Indole alkaloid biosynthesis	7	1	0.221	1.510	1	1	0.000	L-Tryptophan cpd:C00078
31	Pentose phosphate pathway	18	1	0.475	0.744	1	1	0.000	Gluconic acid cpd:C00257
32	Galactose metabolism	26	1	0.525	0.644	1	1	0.049	Stachyose cpd:C01613
33	Glucosinolate biosynthesis	54	1	0.859	0.152	1	1	0.000	L-Tryptophan cpd:C00078

**Table 3:** Pathway analysis of different components of black beans following cooking.

	Pathway	Total	Hits	Raw p	−ln(p)	Holm Adjust	FDR	Impact	Hits Cpd
1	Thiamine metabolism	11	3	0.006	5.157	0.50088	0.5009	0.471	5-Aminoimidazole ribonucleotide cpd:C03373; 5-(2-Hydroxyethyl)-4-methylthiazole cpd:C04294; Thiamine monophosphate cpd:C01081
2	Vitamin B6 metabolism	11	2	0.056	2.884	1	1	0.000	Pyridoxine cpd:C00314; Pyridoxal cpd:C00250
3	Nicotinate and nicotinamide metabolism	12	1	0.355	1.036	1	1	0.000	Nicotinic acid cpd:C00253
4	Glycerophospholipid metabolism	25	2	0.224	1.498	1	1	0.081	O-Phosphoethanolamine cpd:C00346; Citicoline cpd:C00307
5	Sphingolipid metabolism	13	1	0.378	0.973	1	1	0.333	O-Phosphoethanolamine cpd:C00346
6	alpha-Linolenic acid metabolism	23	1	0.570	0.562	1	1	0.160	Alpha-Linolenic acid cpd:C06427
7	Biosynthesis of unsaturated fatty acids	42	1	0.788	0.238	1	1	0.000	Alpha-Linolenic acid cpd:C06427
8	Cysteine and methionine metabolism	34	2	0.345	1.065	1	1	0.138	2-Oxo-4-methylthiobutanoic acid cpd:C01180; 5′-Methylthioadenosine cpd:C00170
9	beta-Alanine metabolism	12	1	0.355	1.036	1	1	0.000	Dihydrouracil cpd:C00429
10	Glycine, serine, and threonine metabolism	30	2	0.668	0.403	1	1	0.000	Betaine cpd:C00719; L-Tryptophan cpd:C00078
11	Arginine and proline metabolism	38	1	0.754	0.282	1	1	0.008	N-Acetyl-L-glutamate 5-semialdehyde cpd:C01250
12	Phenylalanine metabolism	8	1	0.253	1.374	1	1	0.167	Phenylpyruvic acid cpd:C00166
13	Histidine metabolism	16	1	0.443	0.814	1	1	0.000	Imidazoleacetic acid cpd:C02835
14	Phenylalanine, tyrosine, and tryptophan biosynthesis	21	2	0.171	1.767	1	1	0.000	Phenylpyruvic acid cpd:C00166; L-Tryptophan cpd:C00078
15	Tyrosine metabolism	18	1	0.483	0.728	1	1	0.045	Dopamine cpd:C03758
16	Pyruvate metabolism	21	1	0.537	0.622	1	1	0.000	2-Isopropylmalic acid cpd:C02504
17	Valine, leucine, and isoleucine biosynthesis	26	1	0.615	0.486	1	1	0.048	2-Isopropylmalic acid cpd:C02504
18	Tryptophan metabolism	27	1	0.629	0.463	1	1	0.171	L-Tryptophan cpd:C00078
19	Aminoacyl-tRNA biosynthesis	67	1	0.918	0.085	1	1	0.000	L-Tryptophan cpd:C00078
20	Purine metabolism	61	7	0.019	3.981	1	1	0.105	Xanthine cpd:C00385; Guanosine monophosphate cpd:C00144; Guanine cpd:C00242; Deoxyinosine cpd:C05512; Inosine cpd:C00294; Guanosine cpd:C00387; 5-Aminoimidazole ribonucleotide cpd:C03373;
21	Pantothenate and CoA biosynthesis	14	1	0.401	0.915	1	1	0.000	Dihydrouracil cpd:C00429
22	Pyrimidine metabolism	38	3	0.397	0.923	1	1	0.012	Cytidine cpd:C00475; Thymidine cpd:C00214; Dihydrouracil cpd:C00429
23	Stilbenoid, diarylheptanoid, and gingerol biosynthesis	10	1	0.306	1.185	1	1	0.000	3,3′,4′5-Tetrahydroxystilbene cpd:C05901
24	Flavone and flavonol biosynthesis	9	1	0.280	1.273	1	1	0.000	Luteolin cpd:C01514
25	Flavonoid biosynthesis	43	3	0.460	0.776	1	1	0.122	Naringenin cpd:C00509; (-)-Epiafzelechin cpd:C12128; Luteolin cpd:C01514
26	Galactose metabolism	26	2	0.615	0.486	1	1	0.070	Melibiose cpd:C05402; Glucose 1-phosphate cpd:C00103
27	Glucosinolate biosynthesis	54	2	0.866	0.144	1	1	0.010	2-Oxo-4-methylthiobutanoic acid cpd:C01180; L-Tryptophan cpd:C00078
28	Pentose and glucuronate interconversions	12	2	0.066	2.724	1	1	0.000	D-Xylose cpd:C00181; Glucose 1-phosphate cpd:C00103
29	Glycolysis or gluconeogenesis	25	1	0.601	0.510	1	1	0.000	Glucose 1-phosphate cpd:C00103
30	Starch and sucrose metabolism	30	1	0.668	0.403	1	1	0.172	Glucose 1-phosphate cpd:C00103
31	Amino sugar and nucleotide sugar metabolism	41	1	0.780	0.248	1	1	0.110	Glucose 1-phosphate cpd:C00103
32	Isoquinoline alkaloid biosynthesis	6	1	0.196	1.628	1	1	0.500	Dopamine cpd:C03758
33	Indole alkaloid biosynthesis	7	1	0.225	1.491	1	1	0.000	L-Tryptophan cpd:C00078

Total: the number of all metabolites in this pathway; Hits: the number of differential metabolites affecting this pathway; Raw P: *p*-value obtained by enrichment analysis; −ln(p): negative natural logarithm of *p*-value; Holm adjustment: *p*-value adjusted by the Holm–Bonferroni method for multiple hypothesis testing; FDR: *p*-value corrected by the false discovery rate (FDR) method for multiple hypothesis testing; Impact: the impact factors obtained from topology analysis; Hits Cpd: names and KEGG IDS of differential metabolites affecting the pathway; total Cpd: the names of all metabolites contained in this pathway and their KEGG IDS.

## Data Availability

Not applicable.

## Supplementary Materials

The following supporting information can be downloaded at: https://www.mdpi.com/article/10.3390/molecules27123919/s1. Table S1: List of all metabolites, Table S2: List of differential metabolites between raw black beans and cooked black beans, Table S3: List of differential metabolites between raw black beans and steamed black beans, Table S4: List of differential metabolites between cooked black beans and steamed black beans.

## Sample Availability

Samples are not available from the authors.
