# Supplementary material for: Effect of Thermal Processing on the Metabolic Components of Black Beans on Ultra-High-Performance Liquid Chromatography Coupled with High-Field Quadrupole-Orbitrap High-Resolution Mass Spectrometry
Source: Molecules. 2022 Jun 18;27(12):3919. doi: 10.3390/molecules27123919 (PMC9227771; doi:10.3390/molecules27123919)
Supplement: Supplementary file 1 [file molecules-27-03919-s001.zip › molecules-1760059-supplementary.pdf]

Supplementary Materials- Tables

Table S1 Table S1 List of all metabolites

Table S2 List of differential metabolites between unprocessed black soybeans and cooked black soybeans

Table S3 List of differential metabolites between Unprocessed black soybeans and Steamed black soybeans

Table S4 List of differential metabolites between cooked black soybeans and Steamed black soybeans

Table S1 List of all metabolites

	Name	score	RT	m/z	classification	Acquisition mode	unprocessed black soybeans	steamed black soybeans	cooked black soybeans
1	L-Valine	1.000	323.052	116.071	amino acid	[H]-	+	+	+
2	L-Norleucine	1.000	329.139	130.086	amino acid	[H]-	+	+	+
3	Ornithine	0.999	529.169	131.081	amino acid	[H]-	+	+	+
4	L-Proline	0.999	333.297	114.055	amino acid	[H]-	+	+	+
5	Alanylglycine	0.997	357.307	145.061	amino acid	[H]-	+	+	+
6	Pyroglutamic acid	0.995	368.001	128.034	amino acid	[H]-	+	+	+
7	L-Glutamic acid	0.993	417.911	146.045	amino acid	[H]-	+	+	+
8	L-Asparagine	0.993	398.701	131.045	amino acid	[H]-	+	+	+
9	L-Tyrosine	0.989	297.037	180.059	amino acid	[H]-	+	+	+
10	L-Arginine	0.986	536.431	173.103	amino acid	[H]-	+	+	+
11	D-Alanyl-D-alanine	0.960	338.297	159.076	amino acid	[H]-	+	+	+
12	Glycylproline	0.957	367.968	171.077	amino acid	[H]-	+	+	+
13	L-Threonine	0.955	378.284	118.050	amino acid	[H]-	+	+	+
14	N-Acetylornithine	0.954	382.025	173.092	amino acid	[H]-	+	+	+
15	N-Acetyl-L-methionine	0.938	224.008	190.053	amino acid	[H]-	+	+	+
16	Phenylalanylproline	0.934	260.008	261.124	amino acid	[H]-	+	+	+
17	N-Acetyl-L-phenylalanine	0.931	196.430	206.081	amino acid	[H]-	+	+	+
18	N-Acetylglutamic acid	0.894	402.633	188.061	amino acid	[H]-	+	+	+
19	N-Alpha-acetyllysine	0.887	255.685	187.108	amino acid	[H]-	+	+	+
20	N-Acetylleucine	0.887	219.207	172.097	amino acid	[H]-	+	+	+
21	N-Acetyl-L-aspartic acid	0.862	413.332	174.040	amino acid	[H]-	+	+	+
22	gamma-Glutamyltyrosine	0.862	398.465	309.109	amino acid	[H]-	+	+	+
23	Epsilon-(gamma-Glutamyl)-lysine	0.789	477.674	274.140	amino acid	[H]-	+	+	+
24	1-deoxy-1-(N6-lysino)-D-fructose	0.759	424.806	132.029	amino acid	[H]-	+	+	+
25	L-Homoserine	0.738	425.837	118.050	amino acid	[H]-	+	+	+
26	4,5-Dihydroorotic acid	0.736	176.453	157.026	amino acid	[H]-	+	+	+
27	Histidinyl-Serine	0.708	392.438	241.092	amino acid	[H]-	+	+	+
28	N2-gamma-Glutamylglutamine	0.577	435.718	274.104	amino acid	[H]-	+	+	+
29	Glutamylglutamic acid	0.563	475.002	275.088	amino acid	[H]-	+	+	+
30	N-methyl-L-glutamic Acid	0.522	323.235	160.060	amino acid	[H]-	+	+	+
31	N-Acetylvaline	0.480	26.447	158.081	amino acid	[H]-	+	+	+
32	Aspartame	0.387	368.076	293.114	amino acid	[H]-	+	+	+
33	L-Alloisoleucine	1.000	709.002	132.102	amino acid	[H]+	+	+	+
34	Protein serine	1.000	437.210	106.050	amino acid	[H]+	+	+	+
35	D-Proline	1.000	262.811	116.071	amino acid	[H]+	+	+	+
36	(alpha-D-mannosyl)7-beta-D-mannosyl-diacetylchitobiosyl-L-asparagine, isoform A (protein)	1.000	368.834	90.055	amino acid	[H]+	+	+	+
37	L-Serine	0.999	362.438	106.050	amino acid	[H]+	+	+	+
38	Guanidinosuccinic acid	0.999	377.200	176.066	amino acid	[H]+	+	+	+
39	L-Isoleucine	0.999	298.181	132.102	amino acid	[H]+	+	+	+

40	(±)-erythro-Isoleucine	0.999	432.823	132.102	amino acid	[H]+	+	+	+
41	L-Phenylalanine	0.998	281.562	166.086	amino acid	[H]+	+	+	+
42	Glycitein	0.997	114.919	285.075	amino acid	[H]+	+	+	+
43	L-Lysine	0.997	538.369	147.113	amino acid	[H]+	+	+	+
44	D-Serine	0.997	317.650	106.050	amino acid	[H]+	+	+	+
45	Dihydroxyfumitremorgin C	0.997	336.644	412.184	amino acid	[H]+	+	+	+
46	D-Aspartic acid	0.996	424.735	134.045	amino acid	[H]+	+	+	+
47	L-Histidine	0.995	445.375	156.077	amino acid	[H]+	+	+	+
48	Glycyl-Valine	0.995	306.947	175.108	amino acid	[H]+	+	+	+
49	L-Pipecolic acid	0.993	224.347	130.083	amino acid	[H]+	+	+	+
50	L-Methionine	0.991	335.329	150.058	amino acid	[H]+	+	+	+
51	D-Glutamine	0.990	395.613	147.076	amino acid	[H]+	+	+	+
52	Alanyl-Leucine	0.988	253.423	203.139	amino acid	[H]+	+	+	+
53	Pyro-L-glutaminyl-L-glutamine	0.986	188.788	258.108	amino acid	[H]+	+	-	+
54	1-Aminocyclopropanecarboxylic acid	0.985	80.902	102.055	amino acid	[H]+	+	+	+
55	Lysyl-Tyrosine	0.985	245.727	310.173	amino acid	[H]+	+	+	+
56	Isoleucyl-Serine	0.984	281.566	219.133	amino acid	[H]+	+	+	+
57	N-Methyl-a-aminoisobutyric acid	0.984	236.335	118.087	amino acid	[H]+	+	+	+
58	Glycylleucine	0.983	305.099	189.123	amino acid	[H]+	+	+	+
59	Norvaline	0.980	407.905	118.087	amino acid	[H]+	+	+	+
60	(±)-Tryptophan	0.969	285.086	205.097	amino acid	[H]+	+	+	+
61	Glycyl-Serine	0.969	365.758	163.071	amino acid	[H]+	+	+	+
62	3-Methylhistidine	0.962	404.297	170.092	amino acid	[H]+	+	+	+
63	D-Alanine	0.952	388.977	90.055	amino acid	[H]+	+	+	+
64	Lysyl-Leucine	0.950	551.640	260.196	amino acid	[H]+	+	+	+
65	L-Targinine	0.947	526.117	189.134	amino acid	[H]+	+	+	+
66	4-Amino-2-methylenebutanoic acid	0.946	101.720	116.071	amino acid	[H]+	+	+	+
67	Asymmetric dimethylarginine	0.946	524.110	203.150	amino acid	[H]+	+	+	+
68	Tryptophanamide	0.943	71.852	204.113	amino acid	[H]+	+	+	+
69	Glycyltyrosine	0.938	325.335	239.102	amino acid	[H]+	+	+	+
70	Phenylalanyl-Alanine	0.936	254.240	237.123	amino acid	[H]+	+	+	+
71	Prolyl-Threonine	0.934	121.174	217.118	amino acid	[H]+	-	+	-
72	Leucyl-Tyrosine	0.932	231.431	295.165	amino acid	[H]+	+	+	+
73	Leucyl-Isoleucine	0.932	241.988	245.185	amino acid	[H]+	+	+	+
74	N-Ethylglycine	0.925	49.979	104.071	amino acid	[H]+	+	+	+
75	Racemethionine	0.918	219.842	150.058	amino acid	[H]+	+	+	+
76	N2-(3-Hydroxysuccinoyl)arginine	0.914	397.355	291.130	amino acid	[H]+	+	+	+
77	Prolyl-Alanine	0.914	253.531	187.107	amino acid	[H]+	+	+	+
78	Tyrosyl-Methionine	0.911	235.000	313.121	amino acid	[H]+	+	-	-
79	Leucyl-Aspartate	0.910	372.589	247.139	amino acid	[H]+	+	+	+
80	N-Carboxyethyl-g-aminobutyric acid	0.910	411.782	176.092	amino acid	[H]+	+	+	+
81	Prolylphenylalanine	0.909	257.011	263.139	amino acid	[H]+	+	+	+

82	Prolyl-Asparagine	0.907	350.807	230.113	amino acid	[H]+	-	+	+
83	L-2-Amino-3-methylenehexanoic acid	0.904	304.105	144.102	amino acid	[H]+	+	+	+
84	Prolyl-Methionine	0.901	241.145	247.111	amino acid	[H]+	+	+	+
85	Vinylacetylglycine	0.898	321.444	144.065	amino acid	[H]+	+	+	+
86	Prolyl-Aspartate	0.897	426.260	231.097	amino acid	[H]+	+	+	+
87	Aspartyl-Glutamate	0.895	476.136	263.087	amino acid	[H]+	+	+	+
88	Tyrosyl-Tyrosine	0.893	251.489	345.144	amino acid	[H]+	+	-	+
89	Prolyl-Valine	0.892	230.550	215.139	amino acid	[H]+	+	+	+
90	Leucyl-Valine	0.892	218.103	231.170	amino acid	[H]+	+	+	+
91	L-beta-aspartyl-L-glycine	0.891	433.244	191.066	amino acid	[H]+	+	+	+
92	Glutamylthreonine	0.891	426.397	249.108	amino acid	[H]+	+	+	+
93	Tyrosyl-Valine	0.890	241.157	281.149	amino acid	[H]+	+	+	+
94	Arginyl-Leucine	0.888	357.614	288.203	amino acid	[H]+	+	+	+
95	Prolyl-Serine	0.886	320.274	203.103	amino acid	[H]+	+	+	+
96	N-Butyrylglycine	0.885	44.721	146.081	amino acid	[H]+	+	+	+
97	Prolyl-Tyrosine	0.881	335.025	279.133	amino acid	[H]+	-	+	+
98	Arginyl-Methionine	0.879	366.237	306.159	amino acid	[H]+	-	-	+
99	Leucyl-Threonine	0.877	268.442	233.149	amino acid	[H]+	+	+	+
100	Aspartylphenylalanine	0.876	365.811	281.113	amino acid	[H]+	+	+	+
101	Phenylalanyl-Methionine	0.871	208.384	297.126	amino acid	[H]+	-	+	+
102	Glutaminylvaline	0.868	90.484	246.144	amino acid	[H]+	+	+	+
103	Valyl-Aspartate	0.862	402.697	233.113	amino acid	[H]+	+	+	+
104	Histidinyl-Threonine	0.860	364.873	257.124	amino acid	[H]+	+	+	+
105	Valyl-Valine	0.855	235.029	217.155	amino acid	[H]+	+	+	+
106	N-a-Acetyl-L-arginine	0.853	392.121	217.129	amino acid	[H]+	+	+	+
107	beta-Alanine	0.852	53.363	90.055	amino acid	[H]+	+	+	+
108	Arginyl-Gamma-glutamate	0.845	461.563	303.177	amino acid	[H]+	+	+	+
109	Phenylalanyl-Threonine	0.845	234.136	267.134	amino acid	[H]+	-	+	+
110	Prolyl-Glutamine	0.845	367.518	244.129	amino acid	[H]+	+	+	+
111	5-Hydroxy-L-tryptophan	0.842	519.365	221.092	amino acid	[H]+	+	+	+
112	Histidinal	0.836	152.336	140.082	amino acid	[H]+	+	+	+
113	Isoleucyl-Alanine	0.827	237.695	203.139	amino acid	[H]+	+	+	+
114	Prolyl-Tryptophan	0.824	270.408	302.150	amino acid	[H]+	+	+	+
115	Tyrosyl-Asparagine	0.824	328.730	296.123	amino acid	[H]+	+	-	+
116	Isoleucyl-Threonine	0.823	255.244	233.149	amino acid	[H]+	+	+	+
117	Tyrosyl-Serine	0.822	308.249	269.112	amino acid	[H]+	+	-	+
118	Tyrosyl-Threonine	0.820	281.561	283.128	amino acid	[H]+	+	+	+
119	Tryptophyl-Tyrosine	0.817	430.260	368.156	amino acid	[H]+	-	-	+
120	L-Cyclo(alanylglycyl)	0.816	313.192	129.066	amino acid	[H]+	+	+	+
121	Tryptophyl-Arginine	0.815	364.096	361.198	amino acid	[H]+	+	+	+
122	gamma-Glutamylalanine	0.814	428.123	219.097	amino acid	[H]+	+	+	+
123	Pyrroline hydroxycarboxylic acid	0.809	80.962	130.050	amino acid	[H]+	+	+	+
124	Prolylglycine	0.809	331.949	173.092	amino acid	[H]+	+	+	+

125	L-prolyl-L-proline	0.800	333.450	213.123	amino acid	[H]+	+	+	+
126	Isoleucyl-Methionine	0.799	213.612	263.142	amino acid	[H]+	+	+	+
127	gamma-Glutamylleucine	0.798	337.135	261.144	amino acid	[H]+	-	-	+
128	Histidinyl-Aspartate	0.798	436.686	271.103	amino acid	[H]+	+	+	+
129	Alanyl-Proline	0.798	313.133	187.107	amino acid	[H]+	+	+	+
130	Arginyl-Tyrosine	0.796	424.666	338.181	amino acid	[H]+	+	+	+
131	N-Acetyl-L-glutamate 5-semialdehyde	0.793	394.414	174.076	amino acid	[H]+	+	+	+
132	Arginyl-Phenylalanine	0.791	362.105	322.187	amino acid	[H]+	+	+	+
133	Prolylhydroxyproline	0.790	306.003	229.118	amino acid	[H]+	-	-	+
134	Histidinyl-Leucine	0.789	316.622	269.161	amino acid	[H]+	+	+	+
135	L-2-Amino-5-hydroxypentanoic acid	0.788	353.926	134.081	amino acid	[H]+	+	+	+
136	Valyl-Threonine	0.781	242.901	219.134	amino acid	[H]+	+	+	+
137	Tyrosine methylester	0.775	65.219	196.097	amino acid	[H]+	+	+	+
138	Histidinyl-Phenylalanine	0.773	300.749	303.144	amino acid	[H]+	+	+	+
139	Alanyl-Arginine	0.769	438.131	246.156	amino acid	[H]+	+	+	+
140	Histidinyl-Lysine	0.769	517.942	284.171	amino acid	[H]+	+	+	+
141	Tyrosyl-Tryptophan	0.768	229.660	368.160	amino acid	[H]+	+	+	+
142	Hydroxyprolyl-Valine	0.768	124.666	231.134	amino acid	[H]+	-	+	+
143	Lysyl-Valine	0.766	552.461	246.181	amino acid	[H]+	+	+	+
144	Histidinyl-Methionine	0.765	323.480	287.117	amino acid	[H]+	+	+	+
145	Phenylalanyl-Glycine	0.761	255.272	223.108	amino acid	[H]+	+	+	+
146	Methionyl-Threonine	0.752	276.324	251.106	amino acid	[H]+	+	+	+
147	N6-Acetyl-L-lysine	0.750	371.344	189.123	amino acid	[H]+	+	+	+
148	Lysinoalanine	0.747	473.379	234.144	amino acid	[H]+	+	+	+
149	N-[[3-Hydroxy-2-(2-pentenyl)cyclopentyl]acetyl]isoleucine	0.747	141.468	326.232	amino acid	[H]+	-	+	-
150	(3xi,6xi)-Cyclo(alanylvalyl)	0.746	303.026	171.112	amino acid	[H]+	+	+	+
151	Argininosuccinic acid	0.744	488.595	291.130	amino acid	[H]+	+	+	+
152	Histidinyl-Glutamine	0.737	389.432	284.135	amino acid	[H]+	+	+	+
153	Histidinyl-Valine	0.729	333.108	255.144	amino acid	[H]+	+	+	+
154	Histidinyl-Proline	0.729	361.362	253.129	amino acid	[H]+	+	+	+
155	Tyrosyl-Alanine	0.729	268.206	253.118	amino acid	[H]+	+	+	+
156	Alanyl-Isoleucine	0.723	51.957	203.139	amino acid	[H]+	+	+	+
157	Histidinyl-Asparagine	0.716	394.785	270.119	amino acid	[H]+	+	+	+
158	Phenylalanyl-Gamma-glutamate	0.710	283.277	294.145	amino acid	[H]+	+	+	+
159	Tyrosyl-Glutamine	0.709	86.130	310.139	amino acid	[H]+	+	+	+
160	Tyrosyl-Phenylalanine	0.707	215.498	329.149	amino acid	[H]+	+	+	+
161	Lysyl-Arginine	0.706	582.406	303.214	amino acid	[H]+	+	+	+
162	Asparaginyl-Arginine	0.704	472.179	289.161	amino acid	[H]+	-	+	+
163	N5-Acetyl-N2-gamma-L-glutamyl-L-ornithine	0.697	517.126	304.150	amino acid	[H]+	+	+	+
164	5-Hydroxylysine	0.688	529.599	163.107	amino acid	[H]+	+	+	+

165	Prolyl-Lysine	0.684	451.971	244.165	amino acid	[H]+	+	+	+
166	Gamma-glutamyl-L-putrescine	0.681	456.022	218.150	amino acid	[H]+	+	+	+
167	Glutaminyllysine	0.680	471.215	275.171	amino acid	[H]+	+	+	+
168	Arginyl-Serine	0.673	443.955	262.150	amino acid	[H]+	+	+	+
169	Serylvaline	0.669	312.313	205.118	amino acid	[H]+	+	+	+
170	Valyl-Asparagine	0.669	331.395	232.129	amino acid	[H]+	+	+	+
171	Aspartyl-Arginine	0.667	469.880	290.145	amino acid	[H]+	+	+	+
172	Leucyl-Asparagine	0.665	324.391	246.144	amino acid	[H]+	+	+	+
173	Serinyl-Gamma-glutamate	0.664	366.677	234.108	amino acid	[H]+	+	+	+
174	Glutamyllysine	0.663	320.189	276.155	amino acid	[H]+	+	+	+
175	Glutamylphenylalanine	0.660	367.796	295.129	amino acid	[H]+	+	+	+
176	L-Cystine	0.655	453.512	241.030	amino acid	[H]+	+	+	+
177	Tryptophyl-Asparagine	0.654	327.090	319.140	amino acid	[H]+	+	+	+
178	Isoleucyl-Tryptophan	0.643	210.846	318.181	amino acid	[H]+	+	+	+
179	Glutaminylserine	0.641	384.984	234.108	amino acid	[H]+	+	+	+
180	Lysyl-Hydroxyproline	0.637	485.719	260.160	amino acid	[H]+	-	+	-
181	Serylaspartic acid	0.636	437.833	221.076	amino acid	[H]+	+	+	+
182	Tyrosyl-Aspartate	0.636	392.509	297.108	amino acid	[H]+	+	+	+
183	Cysteinyl-Proline	0.632	235.104	219.079	amino acid	[H]+	+	+	+
184	Citrulline	0.624	385.987	176.107	amino acid	[H]+	+	+	+
185	Lysyl-Glycine	0.622	477.962	204.134	amino acid	[H]+	+	+	+
186	2-Methylbutyrylglycine	0.620	325.262	160.097	amino acid	[H]+	+	+	+
187	Isoleucyl-Gamma-glutamate	0.618	283.301	260.160	amino acid	[H]+	+	+	-
188	Serylserine	0.618	373.368	193.082	amino acid	[H]+	+	+	+
189	Hydroxyphenylacetylglycine	0.615	131.551	210.076	amino acid	[H]+	+	+	+
190	Aspartyl-Alanine	0.613	421.136	205.082	amino acid	[H]+	+	+	+
191	Aspartyl-Leucine	0.610	448.712	247.129	amino acid	[H]+	+	+	+
192	Lysyl-Alanine	0.610	514.826	218.150	amino acid	[H]+	+	-	+
193	gamma-Glutamylvaline	0.607	396.464	247.129	amino acid	[H]+	+	+	+
194	N-(4,5-Dihydro-1-methyl-4-oxo-1 H-imidazol-2-yl)alanine	0.604	136.512	186.087	amino acid	[H]+	+	+	+
195	5-Aminopentanamide	0.604	81.602	117.102	amino acid	[H]+	+	+	+
196	Alanyl-Valine	0.949	221.795	189.127	amino acid	[H]+	+	+	+
197	Leucyl-Tyrosine	0.932	231.431	295.165	amino acid	[H]+	-	+	+
198	gamma-Glutamylglutamic acid	0.586	474.535	277.103	amino acid	[H]+	-	+	+
199	Tryptophyl-Methionine	0.578	219.271	336.137	amino acid	[H]+	+	+	+
200	Tyrosyl-Gamma-glutamate	0.590	320.991	310.139	amino acid	[H]+	-	+	+
201	Valyl-Tryptophan	0.571	217.277	304.165	amino acid	[H]+	-	+	+
202	Tryptophyl-Tryptophan	0.550	211.644	391.175	amino acid	[H]+	+	+	+
203	Threoninyl-Serine	0.585	409.705	207.101	amino acid	[H]+	+	-	+
204	Methionyl-Valine	0.528	226.046	249.126	amino acid	[H]+	+	+	-
205	Serylasparagine	0.491	377.862	220.105	amino acid	[H]+	+	+	+
206	Gamma-Aminobutyryl-lysine	0.402	99.530	232.165	amino acid	[H]+	+	+	+
207	Prolyl-Gamma-glutamate	0.579	448.928	244.140	amino acid	[H]+	+	+	+

208	L-Tryptophan	0.526	74.867	203.082	amino acid	[H]+	+	+	+
209	Lysyl-Methionine	0.580	403.764	278.153	amino acid	[H]+	+	+	+
210	3-Furoic acid	0.998	372.607	111.008	organic acid	[H]-	+	+	+
211	Fumaric acid	0.998	247.122	115.002	organic acid	[H]-	+	+	+
212	O-Phosphoethanolamine	0.998	489.014	140.011	organic acid	[H]-	+	+	+
213	L-Malic acid	0.997	447.202	133.013	organic acid	[H]-	+	+	+
214	D-Malic acid	0.994	592.064	133.013	organic acid	[H]-	+	+	+
215	Ketoleucine	0.993	58.181	129.055	organic acid	[H]-	+	+	+
216	Citric acid	0.992	463.459	191.019	organic acid	[H]-	+	+	+
217	Alpha-dimorphecolic acid	0.985	98.323	295.227	organic acid	[H]-	+	+	+
218	Succinic acid	0.984	314.756	117.018	organic acid	[H]-	+	+	+
219	L-2-Hydroxyglutaric acid	0.981	255.465	147.029	organic acid	[H]-	+	+	+
220	4-Dodecylbenzenesulfonic Acid	0.979	28.382	325.184	organic acid	[H]-	+	+	+
221	3-Hydroxybenzoic acid	0.976	47.066	137.023	organic acid	[H]-	+	+	+
222	Taurine	0.975	317.991	124.006	organic acid	[H]-	+	+	+
223	cis-Aconitic acid	0.971	455.433	173.008	organic acid	[H]-	+	+	+
224	3,4-Dihydroxybenzeneacetic acid	0.970	51.182	167.034	organic acid	[H]-	+	+	+
225	(2R,3S)-Piscidic acid	0.957	470.630	255.051	organic acid	[H]-	+	+	+
226	Cyclohexanecarboxylic acid	0.950	44.242	127.075	organic acid	[H]-	+	+	+
227	4-Methylbenzoic acid	0.924	382.998	135.044	organic acid	[H]-	+	+	+
228	Succinylacetone	0.876	65.542	157.050	organic acid	[H]-	+	+	+
229	Isocitric acid	0.874	516.716	173.008	organic acid	[H]-	+	+	+
230	Methylmalonic acid	0.845	221.658	117.018	organic acid	[H]-	+	+	+

231	Malonic acid	0.817	392.763	103.002	organic acid	[H]-	+	+	+
232	p-Anisic acid	0.759	45.356	151.039	organic acid	[H]-	+	+	+
233	3,4-Dimethylbenzoic acid	0.752	216.497	149.060	organic acid	[H]-	+	+	+
234	Terephthalic acid	0.710	61.949	165.022	organic acid	[H]-	+	-	+
235	2-Methylhippuric acid	0.567	214.682	192.066	organic acid	[H]-	+	-	-
236	Salicin	0.491	237.053	271.051	organic acid	[H]-	+	+	+
237	Methyl acrylate	0.991	395.225	87.045	organic acid	[H]+	+	+	+
238	4-Guanidinobutanoic acid	0.975	380.907	146.092	organic acid	[H]+	+	+	+
239	N-(2-Methylpropyl)acetamide	0.811	194.245	116.107	organic acid	[H]+	+	+	+
240	Humulinic acid A	0.613	76.648	267.158	organic acid	[H]+	-	+	-
241	Maleic acid	0.998	276.781	115.002	vitamins	[H]-	+	+	+
242	Nicotinic acid	0.711	239.168	122.024	vitamins	[H]-	+	+	+
243	4-Pyridoxic acid	0.901	46.754	182.045	vitamins	[H]-	+	+	+
244	Riboflavin	0.811	237.357	377.145	vitamins	[H]+	+	+	+
245	24,25-Dihydroxyvitamin D	0.638	31.617	417.336	vitamins	[H]+	+	+	+
246	Nicotinamide riboside	0.884	394.273	253.080	vitamins	[H]-	+	+	+
247	Pyridoxine	0.966	101.418	168.066	vitamins	[H]-	+	+	+
248	Isonicotinic acid	1.000	233.271	124.039	vitamins	[H]+	+	+	+
249	Indole-3-carboxylic acid	0.957	78.009	160.039	alkaloids	[H]-	+	+	+
250	Allantoin	0.956	204.418	157.036	alkaloids	[H]-	+	+	+
251	Imidazoleacetic acid	0.894	95.257	125.034	alkaloids	[H]-	+	+	+
252	Urocanic acid	0.846	199.299	137.034	alkaloids	[H]-	+	+	+
253	Protoporphyrin IX	0.776	309.273	561.254	alkaloids	[H]-	+	+	+
254	1,3,7-Trimethyluric acid	0.434	362.454	209.066	alkaloids	[H]-	+	+	+
255	Hypoxanthine	1.000	236.753	137.046	alkaloids	[H]+	+	+	+
256	Betaine	0.999	293.780	118.086	alkaloids	[H]+	+	+	+
257	3-Isoxazolidinone	0.999	397.408	88.040	alkaloids	[H]+	+	+	+
258	Quinoline	0.998	51.105	130.065	alkaloids	[H]+	+	+	+
259	Trigonelline	0.995	306.699	138.055	alkaloids	[H]+	+	+	+
260	Piperidine	0.995	297.070	86.097	alkaloids	[H]+	+	+	+
261	Xanthine	0.989	330.978	153.040	alkaloids	[H]+	+	+	+
262	Ethyl 2-aminobenzoate	0.953	194.577	166.086	alkaloids	[H]+	+	+	+
263	Fagomine	0.891	321.161	148.097	alkaloids	[H]+	+	+	+
264	Indole	0.846	281.567	118.065	alkaloids	[H]+	+	+	+

265	4-Hydroxy-1H-indole-3-acetonitrile	0.739	51.703	173.071	alkaloids	[H]+	+	+	+
266	Alkaloid A6	0.700	476.159	342.090	alkaloids	[H]+	+	+	+
267	N-Methylcalystegine B2	0.671	414.100	190.107	alkaloids	[H]+	+	+	+
268	Sphinganine	0.653	59.901	302.305	alkaloids	[H]+	+	+	+
269	Nicotine	0.906	69.482	163.123	alkaloids	[H]+	+	-	+
270	Uracil	0.999	174.020	111.019	Nucleotides	[H]-	+	+	+
271	Thymidine	0.989	93.249	241.082	Nucleotides	[H]-	+	+	+
272	Cytidine	0.985	261.061	242.078	Nucleosides	[H]-	+	+	+
273	Uridine	0.982	173.992	243.062	Nucleosides	[H]-	+	+	+
274	Xanthosine	0.973	332.100	283.068	Nucleosides	[H]-	+	+	+
275	Pseudouridine	0.895	263.589	243.062	Nucleosides	[H]-	+	+	+
276	Deoxyinosine	0.999	197.117	253.093	Nucleosides	[H]+	+	+	+
277	Ribitol	0.854	248.984	151.060	Nucleosides	[H]-	+	+	+
278	Cytidine monophosphate	0.780	478.187	322.044	Nucleosides	[H]-	+	+	+
279	Uridine 5'-monophosphate	0.670	435.925	323.028	Nucleosides	[H]-	+	+	+
280	Deoxyribose 5-phosphate	0.654	185.486	213.016	Nucleosides	[H]-	+	+	+
281	N4-Acetylcytidine	0.623	181.203	284.089	Nucleosides	[H]-	+	-	+
282	Adenosine monophosphate	0.547	450.372	346.056	Nucleotides	[H]-	+	+	+
283	Ribothymidine	0.915	151.189	257.077	Nucleotide derivatives	[H]-	+	+	+
284	Cytosine	1.000	215.434	112.051	Nucleotides	[H]+	+	+	+
285	Cytarabine	1.000	260.517	244.093	Nucleosides	[H]+	+	+	+
286	Inosine	0.999	237.500	269.087	Nucleosides	[H]+	+	+	+
287	Nicotinic acid mononucleotide	0.998	359.496	256.081	Nucleotides	[H]+	+	+	+
288	Guanosine	0.995	285.853	284.099	Nucleosides	[H]+	+	+	+
289	Adenosine	0.992	235.061	268.103	Nucleosides	[H]+	+	+	+
290	2-Methylguanosine	0.990	216.393	298.114	Nucleosides	[H]+	+	+	+
291	5-Methylcytidine	0.989	248.189	258.108	Nucleoside	[H]+	+	-	+

					s				
292	Deoxyadenosine	0.989	149.922	252.109	Nucleosides	[H]+	+	+	+
293	5'-Methylthioadenosine	0.975	93.396	298.096	Nucleosides	[H]+	+	+	+
294	Guanosine monophosphate	0.958	479.913	364.065	Nucleosides	[H]+	+	+	+
295	Pyrimidine	0.939	119.976	81.045	Nucleotides	[H]+	+	+	+
296	Citicoline	0.849	462.516	489.115	Nucleotide derivatives	[H]+	+	+	+
297	2-O-Methylcytosine	0.746	168.827	126.066	Nucleotides	[H]+	+	+	+
298	Dihydrouracil	0.831	60.773	115.050	Nucleotides	[H]+	+	+	+
299	Deoxyguanosine	0.996	185.191	268.103	Nucleosides	[H]+	+	+	+
300	3-Methyluridine	0.602	474.198	259.092	Nucleosides	[H]+	+	+	+
301	Uridine diphosphate-N-acetylglucosamine	0.659	450.830	606.073	Nucleosides	[H]-	+	+	+
302	1-Methyladenosine	0.987	132.484	282.120	Nucleosides	[H]+	+	+	+
303	1-Methylhypoxanthine	0.968	155.391	151.061	Nucleotides	[H]+	+	+	+
304	Adenosine 2'-phosphate	0.964	428.737	348.070	Nucleosides	[H]+	+	+	+
305	7-Methyladenine	0.961	258.682	150.077	Nucleotides	[H]+	+	+	+
306	FAPy-adenine	0.951	188.880	154.072	Nucleotides	[H]+	+	+	+
307	Dihydrouracil	0.998	358.269	113.034	Nucleotides	[H]-	+	+	+
308	3'-AMP	0.861	428.259	346.056	Nucleosides	[H]-	-	+	+
309	Niazirinin	0.782	195.480	322.128	Nucleotides	[H]+	+	+	+
310	Thymine	0.918	94.277	127.050	Nucleotides	[H]+	+	-	-
311	2'-O-Methyladenosine	0.997	99.726	282.120	Nucleosides	[H]+	+	+	+
312	Cytidine 2'-phosphate	0.997	456.936	324.059	Nucleosides	[H]+	+	+	+
313	Adenine	1.000	174.493	136.062	Nucleotides	[H]+	+	+	+
314	5-Methylcytosine	0.991	248.238	126.066	Nucleotides	[H]+	+	+	+
315	Guanine	0.998	285.853	152.057	Nucleotides	[H]+	+	+	+
316	1-Methylguanine	0.481	205.999	166.072	Nucleosides	[H]+	+	+	+
317	5-Aminoimidazole ribonucleotide	0.498	407.904	296.066	Nucleotides	[H]+	+	+	+
318	Astragalin	0.942	211.231	447.093	triterpenoid saponins	[H]-	+	+	+
319	Abscisic acid	0.849	83.314	263.128	Sesquiterpenes	[H]-	+	+	+

320	Geranylgeranyl-PP	0.790	242.200	449.186	Terpenes	[H]-	-	+	-
321	Ginkgolide J	0.537	493.305	423.125	terpenoid lactone	[H]-	-	+	-
322	Bilobalide A	0.998	362.203	327.106	Terpenes	[H]+	+	+	+
323	Crispanone	0.996	62.059	335.219	Sesquiterpenes	[H]+	+	-	-
324	primeveroside	0.975	89.880	423.223	Terpenes	[H]+	+	+	+
325	Cynaroside A	0.971	207.499	445.203	Terpenes	[H]+	+	+	+
326	Isopetasoside	0.943	354.139	397.219	Terpenes	[H]+	-	-	+
327	Pyrophaeophorbide	0.942	80.339	535.269	Terpenes	[H]+	+	+	+
328	Solavetivone	0.925	33.335	219.174	Sesquiterpenes	[H]+	+	+	+
329	Licoagroside B	0.892	331.963	433.133	Terpenes	[H]+	+	+	+
330	Glycyrrhetinic acid	0.853	37.902	471.347	Terpenes	[H]+	-	+	+
331	Dehydrovomifoliol	0.832	81.650	223.133	Sesquiterpenes	[H]+	+	+	+
332	S-Furanopetasitin	0.795	495.262	433.203	Terpenes	[H]+	+	+	+
333	Perilloside C	0.794	441.392	317.193	Terpenes	[H]+	+	+	+
334	Cincassiol B	0.790	467.880	401.214	Terpenes	[H]+	+	+	+
335	Soyasaponin A3	0.776	313.033	959.521	Terpenes	[H]+	+	+	+
336	Ganoderic acid Mi	0.761	33.248	545.384	Terpenes	[H]+	+	+	+
337	Citroside A	0.744	221.798	387.200	Terpene glycosides	[H]+	+	+	+
338	Sonchuionoside C	0.737	452.704	387.198	Terpenes	[H]+	-	+	+
339	Cinncassiol C3	0.728	420.665	383.203	Terpenes	[H]+	+	+	+
340	Ganoderal A	0.720	34.374	437.341	Terpenes	[H]+	+	+	+
341	Gerberinol	0.582	190.484	365.102	Terpenes	[H]+	+	+	+
342	Jujubasaponin IV	0.352	306.007	943.524	Terpenes	[H]+	+	+	+
343	Jujubasaponin VI	0.393	270.451	797.468	Terpenes	[H]+	+	+	+
344	Sandosaponin B	0.343	317.124	957.506	Terpenes	[H]+	+	-	+
345	Pisumsaponin II	0.470	300.376	941.511	Terpenes	[H]+	+	+	+
346	L-Arabitol	0.993	97.798	151.060	carbohydrate	[H]-	+	+	+
347	L-Iditol	0.954	309.838	181.071	carbohydrate	[H]-	+	+	+
348	Trehalose	0.950	467.231	341.108	carbohydrate	[H]-	+	+	+
349	Beta-D-Galactose	0.922	259.088	179.055	carbohydrate	[H]-	+	+	+
350	Threonic acid	0.915	334.521	135.029	carbohydrate	[H]-	+	+	+
351	Gluconic acid	0.913	396.520	195.050	carbohydrate	[H]-	+	+	+
352	Galactose 1-phosphate	0.901	496.233	259.022	carbohydrat	[H]-	+	-	+

					e				
353	Stachyose	0.901	517.852	665.215	carbohydrate	[H]-	+	+	+
354	Galactinol	0.889	414.329	341.108	carbohydrate	[H]-	+	+	+
355	D-Xylitol	0.867	274.917	151.060	carbohydrate	[H]-	+	+	+
356	Galactaric acid	0.858	472.398	209.030	carbohydrate	[H]-	+	+	+
357	L-Gulonolactone	0.830	313.432	177.039	carbohydrate	[H]-	+	+	+
358	Rhamnose	0.782	151.216	163.060	carbohydrate	[H]-	+	+	+
359	D-Xylose	0.780	314.593	149.044	carbohydrate	[H]-	+	+	+
360	Melezitose	0.729	667.088	503.162	carbohydrate	[H]-	+	+	+
361	Maltopentaose	0.726	533.553	827.267	carbohydrate	[H]-	+	+	+
362	Maltotetraose	0.674	661.442	665.216	carbohydrate	[H]-	+	+	+
363	Gluconolactone	0.653	123.320	177.039	carbohydrate	[H]-	+	+	+
364	Trehalose 6-phosphate	0.627	497.112	421.075	carbohydrate	[H]-	+	+	+
365	N-Acetyl-glucosamine 1-phosphate	0.594	464.383	300.049	carbohydrate	[H]-	+	+	+
366	Levoglucosan	0.579	223.414	161.045	carbohydrate	[H]-	+	+	+
367	Phenol glucuronide	0.575	363.127	269.066	carbohydrate	[H]-	+	+	+
368	Steviobioside	0.561	400.475	641.314	carbohydrate	[H]-	-	-	+
369	Ethyl glucuronide	0.560	469.649	221.066	carbohydrate	[H]-	+	+	+
370	N-Acetyl-D-glucosamine	0.500	4.597	220.082	carbohydrate	[H]-	+	+	+
371	L-Erythrulose	0.926	201.883	119.034	carbohydrate	[H]-	+	+	+
372	Sucrose	0.999	408.433	365.106	carbohydrate	[H]+	+	+	+
373	D-Maltose	0.997	420.528	365.106	carbohydrate	[H]+	+	+	+
374	(+)-Lyoniresinol 9-glucoside	0.987	155.020	583.236	carbohydrat	[H]+	+	+	+

					e				
375	Turanose	0.987	485.216	365.106	carbohydrate	[H]+	+	+	+
376	Cyanidin 3-glucoside	0.985	343.937	450.114	carbohydrate	[H]+	+	-	-
377	9-Hydroxy-4-methoxypsoralen 9-glucoside	0.971	405.236	395.094	carbohydrate	[H]+	+	+	+
378	Beta-1,4-mannose-N-acetylglucosamine	0.944	351.224	384.153	carbohydrate	[H]+	+	+	+
379	Prenyl arabinosyl-(1->6)-glucoside	0.930	361.358	381.174	carbohydrate	[H]+	-	+	-
380	Calycosin 7-galactoside	0.927	198.127	447.128	carbohydrate	[H]+	+	+	+
381	Taxifolin 3-arabinoside	0.924	36.939	437.105	carbohydrate	[H]+	-	-	+
382	2-Hydroxy-2-phenylacetonitrile O-[b-D-apiosyl-(1->2)-b-D-glucoside]	0.923	360.495	428.153	carbohydrate	[H]+	+	-	-
383	D-Galactose	0.892	517.086	163.060	carbohydrate	[H]+	+	+	+
384	Kojibiose	0.869	387.691	325.112	carbohydrate	[H]+	+	+	+
385	2-Galloylglucose	0.869	367.811	333.085	carbohydrate	[H]+	+	+	+
386	Agaritinal	0.855	378.251	266.111	carbohydrate	[H]+	+	+	+
387	Hydroxytyrosol 1-O-glucoside	0.841	204.073	317.126	carbohydrate	[H]+	+	+	+
388	D-Tagatose	0.800	387.331	163.060	carbohydrate	[H]+	+	+	+
389	Methyl (R)-8-Hydroxy-9-decene-4,6-diynoate glucoside	0.787	327.995	355.138	carbohydrate	[H]+	-	+	-
390	alpha-L-Rhamnopyranosyl-(1->3)-alpha-D-galactopyranosyl-(1->3)-L-fucose	0.769	241.274	473.182	carbohydrate	[H]+	-	-	+
391	beta-D-Glucosamine	0.737	517.046	180.086	carbohydrate	[H]+	+	+	+
392	Ethyl beta-D-glucopyranoside	0.727	223.584	209.103	carbohydrate	[H]+	-	-	+
393	Fructosamine	0.724	466.580	180.087	carbohydrate	[H]+	+	+	+
394	Sorbose 1-phosphate	0.684	455.275	261.036	carbohydrate	[H]+	+	+	+

395	Cholesterol glucuronide	0.652	33.740	563.393	carbohydrate	[H]+	+	+	-
396	Gentiotriose	0.618	469.395	505.176	carbohydrate	[H]+	+	+	+
397	1-[(5-Amino-5-carboxypentyl)amino]-1-deoxyfructose	0.612	476.136	309.165	carbohydrate	[H]+	-	+	-
398	D-Arabinose 5-phosphate	0.608	436.115	213.016	carbohydrate	[H]+	+	-	+
399	Maltotriose	0.993	469.554	527.158	carbohydrate	[H]+	+	+	+
400	Neotrehalose	0.959	661.399	365.105	carbohydrate	[H]+	+	+	+
401	3,3,5-triiodo-L-thyronine-beta-D-glucuronoside	1.000	627.137	132.102	carbohydrate	[H]+	+	+	+
402	Aldehydo-D-xylose	0.764	336.445	149.044	carbohydrate	[H]-	+	+	+
403	Chrysoeriol 7-O-(6''-malonyl-glucoside)	0.590	236.021	549.123	carbohydrate	[H]+	+	-	+
404	1-Deoxy-D-glucitol	0.574	127.835	167.092	carbohydrate	[H]+	+	-	+
405	9-Hydroxy-7-megastigmen-3-one glucoside	0.566	440.555	373.219	carbohydrate	[H]+	+	-	+
406	Lithocholate 3-O-glucuronide	0.443	327.219	553.334	carbohydrate	[H]+	+	-	+
407	4',8-Dimethylgossypetin 3-glucoside	0.498	180.767	509.129	carbohydrate	[H]+	+	+	+
408	4-Methoxybenzyl glucoside	0.469	141.861	301.129	carbohydrate	[H]+	+	+	+
409	Goyaglycoside e	0.483	313.075	781.474	carbohydrate	[H]+	+	+	+
410	4'-Methylliquiritigenin 7-rhamnoside	0.410	143.305	417.154	carbohydrate	[H]+	+	+	+
411	Marmesin rhamnoside	0.411	93.043	393.151	carbohydrate	[H]+	+	+	+
412	Melibiose	0.467	416.475	360.150	carbohydrate	[H]+	+	+	+
413	Citraconic acid	1.000	455.419	129.018	fatty acids	[H]-	+	+	+
414	Myristic acid	1.000	51.084	227.201	fatty acids	[H]-	+	+	+
415	16-Methylheptadecanoic acid	1.000	50.084	283.264	fatty acids	[H]-	+	+	+
416	Ricinoleic acid	0.999	55.227	297.242	fatty acids	[H]-	+	+	+
417	Leucinic acid	0.998	137.152	131.070	fatty acids	[H]-	+	+	+
418	3-Methyl-2-oxovaleric acid	0.997	17.681	129.054	fatty acids	[H]-	+	+	+
419	Mesaconic acid	0.994	467.967	129.018	fatty acids	[H]-	+	+	+
420	Dihydrojasmonic acid	0.992	133.922	211.133	fatty acids	[H]-	+	+	+

421	Myristoleic acid	0.991	716.836	225.185	fatty acids	[H]-	+	+	+
422	9,10-Epoxyoctadecenoic acid	0.991	54.675	295.227	fatty acids	[H]-	+	+	+
423	2-Oxovaleric acid	0.988	39.245	115.039	fatty acids	[H]-	+	+	+
424	16-Hydroxy hexadecanoic acid	0.984	76.769	271.228	fatty acids	[H]-	+	+	+
425	Methylsuccinic acid	0.984	374.444	131.034	fatty acids	[H]-	+	+	+
426	3-Hydroxycapric acid	0.980	104.515	187.133	fatty acids	[H]-	+	+	+
427	Itaconic acid	0.973	66.828	129.018	fatty acids	[H]-	+	+	+
428	9,10-DHOME	0.948	65.547	313.238	fatty acids	[H]-	+	+	+
429	15-Methylpalmitate	0.943	47.091	269.248	fatty acids	[H]-	-	+	+
430	Behenic acid	0.939	47.066	339.326	fatty acids	[H]-	+	+	+
431	(10E,12Z)-9-HODE	0.926	229.011	295.227	fatty acids	[H]-	+	+	+
432	Citramalic acid	0.887	485.862	147.029	fatty acids	[H]-	+	+	+
433	Pelargonic acid	0.870	45.841	157.122	fatty acids	[H]-	-	+	+
434	Traumatic acid	0.862	241.441	227.128	fatty acids	[H]-	+	+	+
435	Dethiobiotin	0.858	307.820	213.124	fatty acids	[H]-	+	+	+
436	2-Methylglutaric acid	0.846	72.160	145.050	fatty acids	[H]-	+	+	+
437	2-Hydroxycinnamic acid	0.820	156.175	163.039	fatty acids	[H]-	+	+	+
438	2,3-Dinor-TXB2	0.791	106.504	341.196	fatty acids	[H]-	-	+	-
439	Prostaglandin F2a	0.786	57.606	353.233	fatty acids	[H]-	-	+	+
440	2-Hydroxystearic acid	0.782	55.416	299.259	fatty acids	[H]-	+	+	+
441	Sinapic acid	0.725	262.788	223.061	fatty acids	[H]-	+	+	+
442	2,3-Dinor-6-keto-prostaglandin F1 a	0.724	103.631	325.202	fatty acids	[H]-	+	+	+
443	(9xi,10xi,12xi)-9,10-Dihydroxy-12 -octadecenoic acid	0.702	79.663	313.238	fatty acids	[H]-	+	+	+
444	(R)-3-Hydroxy-tetradecanoic acid	0.684	71.298	243.196	fatty acids	[H]-	+	+	+
445	3,3-Dimethylglutaric acid	0.680	65.177	159.065	fatty acids	[H]-	+	+	+
446	Suberic acid	0.665	268.397	173.081	fatty acids	[H]-	+	+	+
447	2-Hydroxy-3-methylbutyric acid	0.619	161.627	117.055	fatty acids	[H]-	+	+	+
448	Isopalmitic acid	0.601	229.613	255.232	fatty acids	[H]-	+	+	+
449	2-Isopropylmalic acid	0.600	64.551	175.060	fatty acids	[H]-	+	+	+
450	Dodecanedioic acid	0.588	161.761	229.144	fatty acids	[H]-	-	-	+
451	Azelaic acid	0.585	362.454	187.097	fatty acids	[H]-	+	+	+
452	Palmitic acid	0.575	50.558	255.232	fatty acids	[H]-	+	+	+
453	Glyceric acid	0.500	3.449	105.018	fatty acids	[H]-	+	+	+
454	Oenanthoside A	0.999	344.484	341.121	fatty acids	[H]+	+	+	+
455	20-Hydroxy-PGE2	0.998	107.796	351.213	fatty acids	[H]+	+	+	+
456	gamma-Aminobutyric acid	0.980	170.569	104.071	fatty acids	[H]+	+	+	+
457	Prostaglandin I2	0.997	111.347	353.229	fatty acids	[H]+	+	+	+
458	3-Dehydroxycarnitine	0.991	70.496	146.117	fatty acids	[H]+	+	+	+
459	(9S,10E,12Z,15Z)-9-Hydroxy-10,1 2,15-octadecatrienoic acid	0.930	36.134	277.215	fatty acids	[H]+	+	+	+
460	Aminoadipic acid	0.895	389.438	162.076	fatty acids	[H]+	+	+	+
461	4,8 Dimethylnonanoyl carnitine	0.886	64.175	330.264	fatty acids	[H]+	+	+	+

462	Linoleoyl ethanolamide	0.856	35.904	324.289	fatty acids	[H]+	+	+	+
463	2-Oxo-4-methylthiobutanoic acid	0.847	389.364	149.027	fatty acids	[H]+	-	-	+
464	PS(18:0/22:6(4Z,7Z,10Z,13Z,16Z,19Z))	0.815	178.600	836.541	fatty acids	[H]+	+	+	+
465	L-2,4-diaminobutyric acid	0.802	148.220	119.082	fatty acids	[H]+	+	+	+
466	Prostaglandin H2 2-glyceryl Ester	0.787	57.268	427.266	fatty acids	[H]+	+	-	-
467	Deacetylisovaltrate	0.763	396.479	381.188	fatty acids	[H]+	+	-	-
468	Tetracosahexaenoic acid	0.760	176.027	357.278	fatty acids	[H]+	-	-	-
469	L-Acetylcarnitine	0.755	406.107	204.123	fatty acids	[H]+	+	+	+
470	6,15-Diketo,13,14-dihydro-PGF1a	0.702	477.341	369.224	fatty acids	[H]+	+	+	+
471	(-)-11-Hydroxy-9,15,16-trioxooctadecanoic acid	0.700	434.564	343.209	fatty acids	[H]+	+	+	+
472	PS(18:0/22:0)	0.662	51.001	848.627	fatty acids	[H]+	+	+	+
473	Dimethyl fumarate	0.658	517.126	145.050	fatty acids	[H]+	+	+	+
474	DG(14:1(9Z)/22:4(7Z,10Z,13Z,16Z)/0:0)	0.656	182.582	615.497	fatty acids	[H]+	+	+	-
475	(9R,10S,12Z)-9,10-Dihydroxy-8-oxo-12-octadecenoic acid	0.634	106.250	329.232	fatty acids	[H]+	+	-	+
476	Glycerol	0.741	449.024	93.055	fatty acids	[H]+	+	+	+
477	5-Aminopentanoic acid	0.997	375.751	116.071	fatty acids	[H]-	+	+	+
478	Panaquinquecol 1	0.653	203.871	293.211	fatty acyl	[H]+	+	-	-
479	13-Tetradecene-1,3-diyne-6,7-diol	0.735	64.245	221.153	fatty acyl	[H]+	+	-	-
480	Isopentyl beta-D-glucoside	0.727	159.254	251.150	fatty acyl	[H]+	-	+	+
481	6-Epi-7-isocucurbic acid glucoside	0.721	464.516	375.199	fatty acyl	[H]+	+	+	+
482	Dihydrozeatin-O-glucoside	0.800	411.501	384.187	fatty acyl	[H]+	-	+	+
483	Dieporeticenin	0.789	31.692	573.486	fatty acyl	[H]+	+	+	-
484	Alpha-dimorphecolic acid	0.836	35.959	279.232	fatty acyl	[H]+	+	+	+
485	13-L-Hydroperoxylinoleic acid	0.834	139.604	295.227	fatty acyl	[H]+	+	+	+
486	N-Cyclopropyl-trans-2-cis-6-nonadienamide	0.851	597.101	194.154	fatty acyl	[H]+	+	+	+
487	Polyoxyethylene (600) monoricinoleate	0.877	270.422	341.305	fatty acyl	[H]+	+	+	+
488	Arginyl-Valine	0.871	400.259	274.187	fatty acyl	[H]+	+	+	+
489	Oleamide	0.998	230.549	282.279	fatty acyl	[H]+	+	+	+
490	Lauroyl diethanolamide	0.981	674.365	288.253	fatty acyl	[H]+	+	+	+
491	Palmitic amide	0.966	33.985	256.263	fatty acyl	[H]+	+	+	+
492	10E,12Z-Octadecadienoic acid	1.000	50.022	279.232	fatty acyl	[H]-	+	+	+
493	Alpha-Linolenic acid	1.000	50.315	277.217	fatty acyl	[H]-	+	+	+
494	Linoleic acid	0.992	98.765	279.232	fatty acyl	[H]-	+	+	+
495	Gamma-Linolenic acid	0.992	498.047	277.217	fatty acyl	[H]-	+	+	+
496	13-OxoODE	0.989	38.051	293.212	fatty acyl	[H]-	+	+	+
497	Jasmonic acid	0.988	100.604	209.117	fatty acyl	[H]-	-	+	+
498	(10E,12Z)-(9S)-9-Hydroperoxyoctadeca-10,12-dienoic acid	0.975	99.786	311.223	fatty acyl	[H]-	+	+	+

499	Bovinic acid	0.855	155.727	279.232	fatty acyl	[H]-	+	+	+
500	9,10-epoxyoctadecanoic acid	0.756	37.111	297.242	fatty acyl	[H]-	+	+	+
501	Linoelaidic acid	0.500	37.185	279.232	fatty acyl	[H]-	+	+	+
502	Glycerol 3-phosphate	0.849	453.589	171.005	Glycerophospholipids	[H]-	+	+	+
503	LysoPA(16:0/0:0)	0.703	276.593	409.236	Glycerophospholipids	[H]-	+	+	+
504	LysoPA(18:1(9Z)/0:0)	0.586	273.250	435.252	Glycerophospholipids	[H]-	+	+	+
505	LysoPE(18:3(6Z,9Z,12Z)/0:0)	0.922	230.628	476.277	Glycerophospholipids	[H]+	+	+	+
506	1-Palmitoylglycerophosphoinositol	0.884	273.602	573.302	Glycerophospholipids	[H]+	+	+	+
507	DG(22:5(4Z,7Z,10Z,13Z,16Z)/14:0/0:0)	0.859	32.319	615.497	Glycerolipids	[H]+	+	+	+
508	1-Arachidonoylglycerophosphoinositol	0.832	32.301	621.308	Glycerophospholipids	[H]+	+	-	+
509	2-acetyl-1-alkyl-sn-glycero-3-phosphocholine	0.739	198.728	524.371	Glycerophospholipids	[H]+	-	+	-
510	LysoPC(14:0/0:0)	0.537	228.128	468.308	fatty acids	[H]+	+	+	+
511	LysoPC(16:1(9Z)/0:0)	0.528	225.425	494.325	fatty acids	[H]+	+	+	+
512	LysoPC(18:2(9Z,12Z))	0.630	255.392	520.335	fatty acids	[H]+	-	+	+
513	LysoPC(18:3(6Z,9Z,12Z))	0.534	225.366	518.324	fatty acids	[H]+	+	+	+
514	LysoPE(16:0/0:0)	0.964	229.741	454.293	fatty acids	[H]+	-	+	+
515	LysoPE(18:1(9Z)/0:0)	0.717	226.994	480.309	fatty acids	[H]+	-	+	+
516	LysoPE(20:5(5Z,8Z,11Z,14Z,17Z)/0:0)	0.850	228.739	500.275	fatty acids	[H]+	+	+	+
517	(2'E,4'Z,7'Z,8E)-Colnelenic acid	0.703	63.346	293.211	fatty acids	[H]+	+	+	+
518	MG(0:0/18:3(6Z,9Z,12Z)/0:0)	0.741	35.927	353.268	fatty acids	[H]+	+	-	+
519	MG(0:0/18:4(6Z,9Z,12Z,15Z)/0:0)	0.695	36.239	351.252	fatty acids	[H]+	+	-	+
520	Daidzein	0.999	260.581	253.050	Isoflavones	[H]-	+	+	+
521	Genistein	0.999	37.315	269.045	Isoflavones	[H]-	+	+	+
522	Luteolin 7-glucoside	0.964	189.276	447.093	flavonoids	[H]-	+	+	+
523	(-)-Epiafzelechin	0.962	58.128	273.076	flavonoids	[H]-	+	+	+
524	Quercetin	0.945	91.000	301.034	flavonoids	[H]-	+	+	+
525	Kaempferide	0.944	131.720	299.055	flavonoids	[H]-	+	+	+
526	Diosmetin	0.924	114.005	299.055	flavonoids	[H]-	+	+	+
527	2-(3,4-dihydroxyphenyl)-3,5-dihydroxy-7-methoxy-4H-chromen-4-one	0.880	51.974	315.051	flavonoids	[H]-	+	+	+
528	5,7-dihydroxy-2-(4-hydroxy-3-methoxyphenyl)-3-{[3,4,5-trihydroxy-6-(hydroxymethyl)oxan-2-yl]oxy}-4H-chromen-4-one	0.782	179.797	477.104	flavonoids	[H]-	+	+	+

529	Norizalpinin	0.754	294.630	269.045	flavonoids	[H]-	+	+	+
530	Mulberrin	0.979	169.612	421.162	flavonoids	[H]-	+	+	+
531	Isoquercitrin	0.729	314.534	463.088	flavonoids	[H]-	+	+	+
532	Liquiritin	0.713	164.280	417.119	flavonoids	[H]-	+	+	+
533	Naringin	0.653	224.806	579.172	flavonoids	[H]-	-	-	+
534	Panasenoside	0.628	300.843	609.147	flavonoids	[H]-	+	+	+
535	3,5,7-trihydroxy-2-(4-hydroxyphenyl)-3,4-dihydro-2H-1-benzopyran-4-one	0.521	58.759	287.056	flavonoids	[H]-	+	+	+
536	5,7-dihydroxy-2-phenyl-6-[3,4,5-trihydroxy-6-(hydroxymethyl)oxan-2-yl]-8-(3,4,5-trihydroxyoxan-2-yl)-4H-chromen-4-one	0.408	81.574	547.145	flavonoids	[H]-	+	+	+
537	Phlorizin	0.351	225.433	435.130	flavonoids	[H]-	+	+	+
538	(E)-3-(4-Hydroxyphenyl)-2-propenal	0.969	281.452	147.044	Lignans	[H]-	+	+	+
539	4-Methoxycinnamic acid	0.939	369.638	177.055	Lignans	[H]-	+	+	+
540	Hydrocinnamic acid	0.913	370.661	149.060	phenylpropanoids	[H]-	+	+	+
541	3,4-Dihydroxyhydrocinnamic acid	0.842	96.278	181.050	phenylpropanoids	[H]-	+	+	+
542	3-(2-Hydroxyphenyl)propanoic acid	0.821	117.611	165.055	phenylpropanoids	[H]-	+	+	+
543	Zeranol	0.462	31.719	321.174	phenylpropanoids	[H]-	+	+	+
544	Procyanidin B2	0.861	247.977	577.135	Anthocyanins	[H]-	+	+	+
545	3,3',4'5-Tetrahydroxystilbene	0.933	116.753	243.066	stilbene	[H]-	+	+	+
546	Marmesin	0.897	136.361	245.082	Coumarins	[H]-	+	+	+
547	Protocatechuic acid	0.812	39.594	153.018	Phenolic acid	[H]-	+	+	+
548	Gallic acid	0.649	32.854	169.013	Phenols	[H]-	+	+	+
549	Pyrocatechol	0.993	44.801	109.028	Phenols	[H]-	+	+	+
550	benzene-1,2,4-triol	0.967	392.763	125.023	Phenols	[H]-	+	+	+
551	2-Pyrocatechuic acid	0.998	301.521	153.018	Phenolic acids	[H]-	+	+	+
552	Feruloyl-2-hydroxyputrescine	1.000	152.309	281.149	Lignans	[H]+	+	+	+
553	Maltol	1.000	69.911	127.039	Phenols	[H]+	+	+	+
554	Kaempferol	0.998	299.527	287.055	flavonoids	[H]+	+	+	+
555	11-Methylgerberinol	0.998	405.415	379.121	Coumarins	[H]+	+	+	+
556	Astragalin	0.996	189.703	449.107	flavonoids	[H]+	+	+	+
557	Cosmosiin	0.996	201.839	433.112	flavonoids	[H]+	+	+	+
558	Biochanin A	0.995	193.060	285.075	Isoflavones	[H]+	+	+	+
559	Luteolin	0.994	521.279	287.054	flavonoids	[H]+	+	+	+

560	Daidzin	0.992	193.587	417.117	flavonoids	[H]+	+	+	+
561	Peonidin-3-glucoside	0.990	153.425	463.123	flavonoids	[H]+	+	+	+
562	6-Hydroxydaidzein 4'-glucoside	0.989	155.042	433.112	Isoflavones	[H]+	+	+	+
563	Sideritiflavone	0.989	102.955	361.091	flavonoids	[H]+	+	+	+
564	Genistein 5-glucoside	0.988	126.809	433.112	Isoflavones	[H]+	+	+	+
565	Glycitin	0.985	164.213	447.128	Isoflavones	[H]+	+	+	+
566	Naringenin	0.984	175.125	273.075	flavonoids	[H]+	+	+	+
567	3',4',5,7-Tetrahydroxyisoflavanone	0.982	227.153	289.070	flavonoids	[H]+	+	+	+
568	Isorhamnetin	0.981	192.786	317.066	flavonoids	[H]+	+	+	+
569	Luteolin 4'-glucoside	0.971	523.336	449.107	flavonoids	[H]+	+	+	+
570	6''-O-Acetylgenistin	0.969	161.269	475.123	Isoflavones	[H]+	+	+	+
571	Pelargonin	0.968	127.278	595.166	flavonoids	[H]+	+	+	+
572	6''-Malonylgenistin	0.965	240.327	519.114	Isoflavones	[H]+	+	+	+
573	6''-O-Acetyldaidzin	0.964	61.653	459.129	Isoflavones	[H]+	+	+	+
574	Cyanidin 3-(4-acetylglucoside)	0.960	117.334	491.118	flavonoids	[H]+	+	+	+
575	Carthamone	0.959	475.950	449.107	Lignans	[H]+	+	+	+
576	6''-O-Acetylglycitin	0.946	62.523	489.139	Isoflavones	[H]+	+	+	+
577	Luteolin 7-galactoside	0.944	241.710	449.107	flavonoids	[H]+	+	+	+
578	Kaempferol 3-(6-acetylgalactoside)	0.944	148.659	491.119	polyphenols	[H]+	+	+	+
579	Apigenin 7-O-(6''-O-acetylglucoside)	0.943	107.416	475.124	flavonoids	[H]+	+	+	+
580	Epicatechin	0.938	176.579	291.086	flavonoids	[H]+	+	-	+
581	Genistin	0.937	219.125	433.113	Isoflavones	[H]+	+	+	+
582	Acetaminophen	0.937	102.583	152.070	Phenols	[H]+	+	+	+
583	Occidentoside	0.930	517.126	705.182	flavonoids	[H]+	+	+	+
584	Apiin	0.929	210.098	565.155	flavonoids	[H]+	+	+	+
585	Epicatechin 3-O-(4-methylgallate)	0.909	187.905	457.110	flavonoids	[H]+	+	+	+
586	Subaphylline	0.905	210.486	265.154	Lignans	[H]+	+	+	+
587	(±)-2-(1-Methylpropyl)-4,6-dinitrophenol	0.887	473.430	241.082	Phenols	[H]+	+	+	+
588	Petunidin 3-glucoside	0.887	192.712	479.119	polyphenols	[H]+	+	+	+
589	Cyanidin 3-laminaribioside	0.881	298.582	611.160	Anthocyanins	[H]+	+	+	+
590	Phenol	0.866	325.557	95.050	Phenols	[H]+	+	+	+
591	Trilobatin	0.865	202.976	437.143	flavonoids	[H]+	+	+	+
592	(Z)-Resveratrol 3-(6''-sulfoglucopyranoside)	0.865	189.620	471.089	polyphenols	[H]+	+	+	+
593	(-)-Epigallocatechin 3-(4-methyl-gallate)	0.858	277.151	473.106	Phenolic acids	[H]+	+	+	+
594	6'-Malonyltrifolirhizin	0.844	260.005	533.128	Isoflavones	[H]+	+	+	+
595	Malvidin 3-glucoside	0.839	100.318	493.133	anthocyanin	[H]+	+	+	+

596	2'-Hydroxygenistein 7-(6''-malonylglucoside)	0.834	266.351	535.108	Isoflavones	[H]+	+	+	+
597	Yuccaol C	0.831	468.801	543.131	phenylpropanoids	[H]+	+	+	+
598	Dihydrodaidzin	0.814	120.435	419.133	flavonoids	[H]+	+	+	+
599	Diferuloylputrescine	0.808	56.704	441.201	Lignans	[H]+	+	-	+
600	2-Ethylacrylic acid	0.800	407.108	101.060	flavonoids	[H]+	+	+	+
601	Quercitrin	0.762	189.748	487.064	flavonoids	[H]+	+	+	+
602	Rhoifolin	0.731	299.550	579.171	polyphenols	[H]+	+	+	+
603	6''-O-Malonyldaidzin	0.723	214.586	503.119	Isoflavones	[H]+	+	+	+
604	Flavidulol C	0.711	415.988	515.319	flavonoids	[H]+	-	+	-
605	Ononin	0.684	118.233	431.134	Isoflavones	[H]+	+	+	+
606	[10]-Paradol	0.664	230.540	335.257	Phenols	[H]+	+	+	+
607	trans-Isoasarone	0.641	322.720	209.117	flavonoids	[H]+	+	+	+
608	Dianhydroaurasperone C	0.602	487.248	557.147	flavonoids	[H]+	+	+	+
609	Phlorin	0.994	164.109	289.091	polyphenols	[H]+	+	+	+
610	4-Aminophenol	0.944	51.147	110.060	phenol	[H]+	+	+	+
611	2-(1,2-Diamino-1-propenyl)phenol	0.699	55.811	165.102	phenol	[H]+	+	+	+
612	Citrusinine II	0.448	60.782	288.086	flavonoids	[H]-	+	+	+
613	5-Heptyltetrahydro-2-oxo-3-furancarboxylic acid	0.525	58.136	229.143	Isoflavones	[H]-	+	+	+
614	6''-O-Malonylwistin	0.829	91.712	547.145	Isoflavones	[H]-	+	+	-
615	Soyasaponin IV	0.587	257.040	767.458	flavonoids	[H]-	+	+	-
616	5,7-Dihydroxy-2',6-dimethoxyisoflavone 7-rhamnoside	0.541	190.466	461.144	Isoflavones	[H]-	+	+	+
617	4',5,6-Trimethylscutellarein 7-glucoside	0.800	181.813	491.154	flavonoids	[H]-	+	+	+
618	Calendulaglycoside E	0.453	262.060	795.450	flavonoids	[H]-	+	+	+
619	Taurocholic acid	0.652	333.665	514.287	bile acid	[H]-	+	+	-
620	6-Deoxyalcoholsterone	0.716	32.220	449.362	bile acid	[H]+	+	+	+
621	Quinic acid	0.986	356.415	191.055	alcohol	[H]-	+	+	+
622	2-(3,4-dihydroxyphenyl)-3,4-dihydro-2H-1-benzopyran-3,5,7-triol	0.930	98.050	289.071	alcohol	[H]-	+	+	+
623	myo-Inositol	0.900	412.688	179.055	alcohol	[H]-	+	+	+
624	Glucose 1-phosphate	0.559	476.469	259.022	alcohol	[H]-	+	+	+
625	Diacetone alcohol	0.980	72.839	115.075	alcohol	[H]-	+	+	-
626	2-Phenylethanol	0.984	183.349	105.070	alcohol	[H]+	+	+	+
627	Phytosphingosine	0.916	38.587	318.300	alcohol	[H]+	+	+	+
628	(±)-2-Pentanethiol	0.897	394.777	105.074	alcohol	[H]+	+	+	+
629	1-Hexanethiol	0.853	375.193	119.090	alcohol	[H]+	+	+	+
630	alpha-[3-(Nitrosoamino)propyl]-3-pyridinemethanol	0.778	367.518	196.108	alcohol	[H]+	-	+	-

631	Santalyl acetate	0.771	78.710	263.200	alcohol	[H]+	+	+	+
632	Dehydrophytosphingosine	0.757	160.408	316.284	alcohol	[H]+	+	+	+
633	7,9-Illudadiene-3,14-diol	0.709	94.253	235.169	alcohol	[H]+	+	-	-
634	3-(4-Hydroxyphenyl)-1-propanol	0.509	34.180	153.091	alcohol	[H]+	+	+	+
635	3,4-Dihydroxycinnamoyl-(Z)-2-(3, 4-dihydroxyphenyl)ethenol	0.590	152.353	315.085	alcohol	[H]+	-	+	+
636	3,4-Dihydroxybenzaldehyde	1.000	315.986	137.023	aldehyde	[H]-	+	+	+
637	Succinic acid semialdehyde	0.536	320.428	101.023	aldehyde	[H]-	+	+	+
638	4-Hydroxybenzaldehyde	0.500	38.684	121.028	aldehyde	[H]-	+	+	+
639	3-(3-Furanyl)-2-methyl-2-propenal	0.997	174.435	137.065	aldehyde	[H]+	+	+	+
640	3-Acetamidobutanal	0.992	44.291	130.086	aldehyde	[H]+	+	+	+
641	Benzaldehyde	0.978	368.193	107.049	aldehyde	[H]+	+	+	+
642	4-Trimethylammoniobutanal	0.947	54.612	130.123	aldehyde	[H]+	+	+	+
643	4-Aminobutyraldehyde	0.947	548.446	88.076	aldehyde	[H]+	+	+	+
644	Betaine aldehyde	0.898	272.815	102.092	aldehyde	[H]+	+	+	+
645	5-Aminopentanal	0.872	231.942	102.092	aldehyde	[H]+	+	+	+
646	xi-3-(4-Isopropylphenyl)-2-methyl propanal	0.837	33.956	191.143	aldehyde	[H]+	+	+	+
647	2-Benzofurancarboxaldehyde	0.813	325.745	147.044	aldehyde	[H]+	+	+	+
648	2-Hexenal	0.799	193.749	99.081	aldehyde	[H]+	+	+	+
649	3-(4-Isopropylphenyl)propanal	0.780	62.420	177.127	aldehyde	[H]+	+	+	+
650	Imidazole-4-acetaldehyde	0.751	63.761	111.055	aldehyde	[H]+	+	+	+
651	3-(2-Furanyl)-2-phenyl-2-propenal	0.739	161.669	199.075	aldehyde	[H]+	+	+	+
652	4-Oxo-2-nonenal	0.670	172.180	155.107	aldehyde	[H]+	+	+	+
653	Pyridoxal	0.649	69.656	168.065	aldehyde	[H]+	+	+	+
654	Methylimidazole acetaldehyde	0.647	190.504	125.071	aldehyde	[H]+	+	+	+
655	Vanillin 3-(L-menthoxy)propane-1,2-diol acetal	0.626	50.251	365.231	aldehyde	[H]+	+	+	+
656	Phenylacetaldehyde	0.980	325.403	121.065	aldehyde	[H]+	+	+	+
657	Indoleacetaldehyde	0.966	51.032	160.075	aldehyde	[H]+	+	+	+
658	2-Hydroxybenzaldehyde	1.000	129.627	121.028	aldehyde	[H]-	+	+	+
659	1H-Indole-3-carboxaldehyde	1.000	63.835	144.044	aldehyde	[H]-	+	+	+
660	Oxoglutaric acid	0.989	388.681	145.013	acid	[H]-	+	+	+
661	Phenylpyruvic acid	0.937	186.597	163.039	acid	[H]-	+	+	+
662	Adipic acid	0.830	245.326	145.050	acid	[H]-	+	+	+
663	4-Hydroxyphenylpyruvic acid	0.805	369.670	179.034	acid	[H]-	+	+	+
664	Shikimic acid	0.750	86.736	173.045	acid	[H]-	+	+	+
665	3,4-Dihydroxymandelic acid	0.738	26.297	183.029	acid	[H]-	+	+	+
666	Phenylacetic acid	0.730	205.592	135.044	acid	[H]-	+	+	+
667	trans-trans-Muconic acid	0.729	30.824	141.016	acid	[H]-	+	+	+
668	3,4,5-trihydroxycyclohex-1-ene-1-c arboxylic acid	0.702	105.056	173.045	acid	[H]-	+	+	+
669	cis,cis-Muconic acid	0.680	134.683	141.018	acid	[H]-	+	+	+

670	Syringic acid	0.674	85.059	197.045	acid	[H]-	+	+	+
671	Phthalic acid	0.643	132.115	165.018	acid	[H]-	+	+	+
672	Pyrrole-2-carboxylic acid	0.577	174.011	110.024	acid	[H]-	+	+	+
673	Oxoadipic acid	0.568	73.965	159.029	acid	[H]-	+	+	+
674	Isohomovanillic acid	0.500	37.922	181.050	acid	[H]-	+	+	+
675	3-(4-hydroxy-3-methoxyphenyl)prop-2-enoic acid	0.851	167.700	193.050	acid	[H]-	+	+	+
676	Formylanthranilic acid	0.783	63.284	164.034	acid	[H]-	-	+	-
677	Phenylglyoxylic acid	0.550	56.198	149.023	acid	[H]-	+	+	+
678	Diaminopimelic acid	0.938	362.079	189.076	acid	[H]-	+	+	+
679	Homovanillic acid	0.881	51.639	181.050	acid	[H]-	+	+	+
680	2-Pyrrolidineacetic acid	0.995	65.210	130.086	acid	[H]+	+	+	+
681	L-trans-4-Methyl-2-pyrrolidinecarboxylic acid	0.994	255.163	130.086	acid	[H]+	+	+	+
682	m-Aminobenzoic acid	0.987	334.946	138.055	acid	[H]+	+	+	+
683	D-1-Piperideine-2-carboxylic acid	0.984	474.237	128.071	acid	[H]+	+	+	+
684	Phosphoric acid	0.983	365.356	98.985	acid	[H]+	+	+	+
685	Pipecolic acid	0.982	533.354	130.097	acid	[H]+	+	+	+
686	(±)-4-Methylene-2-pyrrolidinecarboxylic acid	0.969	696.561	128.071	acid	[H]+	+	+	+
687	1-Pyrroline-2-carboxylic acid	0.967	335.197	114.055	acid	[H]+	+	+	+
688	Pi-Methylimidazoleacetic acid	0.944	325.650	141.066	acid	[H]+	+	+	+
689	Colubrinic acid	0.936	32.325	453.334	acid	[H]+	+	+	+
690	alpha-Hydroxy-1-methyl-1H-indole-3-propanoic acid	0.721	209.956	220.097	acid	[H]+	+	+	+
691	3-Methyl-5-pentyl-2-furanundecanoic acid	0.710	177.354	337.273	acid	[H]+	+	+	+
692	1,4'-Bipiperidine-1'-carboxylic acid	0.699	242.901	213.159	acid	[H]+	+	+	+
693	[8]-Paradyl acetate	0.637	36.838	349.237	acid	[H]+	+	+	+
694	Sebiferic acid	0.617	303.963	441.373	acid	[H]+	+	+	+
695	Betulinic acid	0.601	253.623	457.367	acid	[H]+	+	+	+
696	Benzoic acid	0.996	326.112	123.044	acid	[H]+	+	+	+
697	2-cis,6-trans,10-trans-Geranylgeranyl diphosphate	0.802	410.131	451.203	acid	[H]+	-	-	+
698	L-trans-alpha-Amino-2-carboxycyclopropaneacetic acid	0.934	428.155	160.060	acid	[H]+	+	+	+
699	Pyrrolidonecarboxylic acid	1.000	320.278	128.034	acid	[H]-	+	+	+
700	L-Dihydroorotic acid	1.000	321.383	157.024	acid	[H]-	+	+	+
701	Norophthalmic acid	0.581	437.290	276.119	acid	[H]+	+	-	+
702	2-Aminoheptanedioic acid	0.565	285.538	176.091	acid	[H]+	+	-	+
703	(R)-mandelic Acid	0.482	67.757	135.044	acid	[H]+	-	+	+
704	2',4',6'-Trihydroxyacetophenone	0.894	158.107	167.034	ketone	[H]-	+	+	+
705	2-Piperidinone	1.000	55.517	100.076	ketone	[H]+	+	+	+
706	2'-Hydroxyacetophenone	0.993	51.036	137.060	ketone	[H]+	+	+	+

707	Indoxyl	0.975	144.553	134.060	ketone	[H]+	+	+	+
708	alpha-Furyl methyl diketone	0.975	92.524	139.039	ketone	[H]+	+	+	+
709	Aminoacetone	0.967	332.656	74.061	ketone	[H]+	+	+	+
710	5-Methyl-2(3H)-furanone	0.944	387.384	99.044	ketone	[H]+	+	+	+
711	1H-Indole-2,3-dione	0.936	50.202	148.039	ketone	[H]+	+	+	+
712	5-Phenyl-1,3-oxazinane-2,4-dione	0.936	50.640	192.065	ketone	[H]+	+	+	+
713	Menthone 1,2-glyceryl ketal	0.913	437.548	298.100	ketone	[H]+	+	+	+
714	2-Pyrrolidinone	0.912	394.397	86.061	ketone	[H]+	+	+	+
715	6-[2,3-Dihydroxy-1-(hydroxymethyl)propyl]-1,2-dihydro-7-hydroxy-9-methoxy-cyclopenta[c][1]benzopyran-3,4-dione	0.894	409.850	351.104	ketone	[H]+	+	+	+
716	1-(2-Thienyl)-1-heptanone	0.867	532.376	197.100	ketone	[H]+	+	+	+
717	1-(1-Pyrrolidinyl)-2-butanone	0.853	232.592	142.123	ketone	[H]+	+	+	+
718	Tetrahydro-4-hydroxy-1H-isoindole-1,3(2H)-dione	0.774	86.994	168.065	ketone	[H]+	+	+	+
719	5-Hydroxybuspirone	0.761	403.671	402.245	ketone	[H]+	-	-	+
720	Tetrahydro-5-hydroxy-1H-isoindole-1,3(2H)-dione	0.750	117.118	168.065	ketone	[H]+	+	+	+
721	2-(5,8-Tetradecadienyl)cyclobutanone	0.674	34.991	263.237	ketone	[H]+	+	+	+
722	3,5,7-trihydroxy-2-(4-hydroxyphenyl)-3,4-dihydro-2H-1-benzopyran-4-one	0.521	58.759	287.056	ketone	[H]+	+	+	-
723	3,4-Dihydro-2H-1-benzopyran-2-one	0.513	280.690	149.060	ketone	[H]+	+	+	-
724	3-[(3-Methylbutyl)nitrosoamino]-2-butanone	0.576	495.802	187.144	ketone	[H]+	+	+	+
725	3alpha,4,7,7alpha-Tetrahydro-4-hydroxy-1H-isoindole-1,3(2H)-dione	0.774	86.994	168.065	ketone	[H]+	+	-	+
726	(3R,8E)-3-Hydroxy-5,8-megastigmadien-7-one	0.560	229.045	209.153	ketone	[H]+	+	-	+
727	Methyl jasmonate	0.957	80.035	223.133	ester	[H]-	+	+	+
728	Methyl vanillate	0.862	74.220	181.050	ester	[H]-	+	+	+
729	Dihydrolipoate	0.540	67.353	207.050	ester	[H]-	+	+	+
730	Ethylparaben	0.585	45.843	165.055	ester	[H]-	+	+	+
731	Erythrono-1,4-lactone	0.997	480.902	117.018	ester	[H]-	+	+	+
732	4-Hydroxy-2-butenoic acid gamma-lactone	0.992	468.792	85.029	ester	[H]+	+	+	+
733	Dukunolide B	0.977	353.105	499.157	ester	[H]+	+	+	+
734	Phenyl salicylate	0.966	127.193	215.070	ester	[H]+	+	+	+
735	Thiamine monophosphate	0.952	514.422	345.078	ester	[H]+	+	+	+
736	Butyl 3-O-caffeoylquinate	0.922	322.670	411.162	ester	[H]+	+	+	+
737	Cinnamyl benzoate	0.872	528.531	239.106	ester	[H]+	+	+	+

738	Linalyl phenylacetate	0.828	36.449	273.184	ester	[H]+	+	+	+
739	Thujyl 19-trachylobanoate	0.819	33.929	439.357	ester	[H]+	+	+	+
740	Acetyl tributyl citrate	0.777	441.885	403.229	ester	[H]+	+	+	+
741	Hexyl heptanoate	0.749	475.681	639.245	ester	[H]+	-	+	-
742	Alantolactone	0.727	52.502	233.149	ester	[H]+	+	+	+
743	3-Hydroxyadipic acid 3,6-lactone	0.705	387.277	145.050	ester	[H]+	+	+	+
744	Butyl levulinate	0.694	36.855	173.117	ester	[H]+	+	+	+
745	3-Methylbutyl 2-furanbutanoate	0.657	208.097	225.148	ester	[H]+	+	+	+
746	8-Ocimenyl acetate	0.620	207.372	195.138	ester	[H]+	+	+	+
747	2-Carboxy-4-dodecanolide	0.614	207.351	243.159	ester	[H]+	+	+	+
748	2-Hydroxyethanesulfonate	0.969	149.026	124.990	other	[H]-	+	+	+
749	1,2,3-Trihydroxybenzene	0.949	477.273	125.023	other	[H]-	+	+	+
750	Hydrogen phosphate	0.929	177.417	96.969	other	[H]-	+	+	+
751	Indolelactic acid	0.926	199.154	204.066	other	[H]-	+	+	+
752	p-Hydroxymandelic acid	0.922	32.998	167.037	other	[H]-	+	+	+
753	(-)-Gossypol	0.856	465.040	517.189	other	[H]-	+	+	+
754	Meconine	0.832	216.772	193.050	other	[H]-	+	+	+
755	Hypogeic acid	0.803	38.051	253.217	other	[H]-	+	+	+
756	Oxazepam	0.778	51.934	285.040	other	[H]-	+	+	+
757	5,7-dihydroxy-6-methoxy-2-phenyl-4H-chromen-4-one	0.774	184.010	283.051	other	[H]-	+	+	+
758	N1-(2-Hydroxyethyl)flurazepam	0.746	182.110	331.067	other	[H]-	+	+	+
759	Aflatoxin B1	0.710	152.216	311.054	other	[H]-	+	+	+
760	Bilirubin	0.499	300.828	583.260	other	[H]-	+	+	+
761	alpha-Mangostin	0.459	295.024	409.165	other	[H]-	+	+	+
762	Dopamine	0.447	310.717	299.077	other	[H]-	+	+	+
763	(1R,2S,3R)-2-Acetyl-4(5)-(1,2,3,4-tetrahydroxybutyl)imidazole	0.344	440.998	229.082	other	[H]-	+	-	+
764	Methylgingerol	0.544	38.908	307.191	other	[H]-	+	+	-
765	2-Hydroxypyridine	0.999	52.794	96.045	other	[H]+	+	+	+
766	1-Butylamine	0.999	52.850	74.097	other	[H]+	+	+	+
767	Protoanemonin	0.998	517.104	97.029	other	[H]+	+	+	+
768	Choline	0.998	271.064	104.107	other	[H]+	+	+	+
769	Dukunolide A	0.997	344.110	483.163	other	[H]+	+	+	+
770	3-Amino-2-piperidone	0.997	218.187	115.087	other	[H]+	+	+	+
771	5-Oxo-2(5H)-isoxazolepropanenitrile	0.995	199.257	139.050	other	[H]+	+	+	+
772	Nervonyl carnitine	0.994	37.261	102.128	other	[H]+	+	+	+
773	2,6-Dimethylpyrazine	0.993	553.990	109.076	other	[H]+	+	+	+
774	Epsilon-caprolactam	0.993	51.028	114.092	other	[H]+	+	+	+
775	Acetylcholine	0.993	196.998	146.117	other	[H]+	+	+	+
776	Phosphorylcholine	0.993	552.038	184.073	other	[H]+	+	+	+
777	2-Aminonaphthalene	0.991	285.047	144.081	other	[H]+	+	+	+
778	Methylpyrazine	0.990	401.959	95.061	other	[H]+	+	+	+

779	Creatinine	0.985	21.361	114.066	other	[H]+	+	+	+
780	5-(2-Hydroxyethyl)-4-methylthiazole	0.985	48.170	144.048	other	[H]+	+	+	+
781	N,N-Dimethylformamide	0.982	90.747	74.061	other	[H]+	+	+	+
782	7-Hydroxymethyl-12-methylbenz[a]anthracene sulfate	0.977	70.362	353.084	other	[H]+	+	+	+
783	3-Indoleacetonitrile	0.967	52.894	157.076	other	[H]+	+	+	+
784	Harderoporphyrin	0.966	76.226	609.270	other	[H]+	+	+	+
785	(+)-2,3-Dihydro-3-methyl-1H-pyrrole	0.961	202.707	84.081	other	[H]+	+	+	+
786	2,3-Dihydro-5-(3-hydroxypropanoyl)-1H-pyrrolizine	0.958	53.723	180.102	other	[H]+	+	+	+
787	1,2,6,8-Tetrahydroxy-3-methylanthraquinone 2-O-b-D-glucoside	0.958	497.748	449.106	other	[H]+	+	+	+
788	2,3,4,5-Tetrahydropiperidine-2-carboxylate	0.956	619.807	128.071	other	[H]+	+	+	+
789	5-Acetyl-2,4-dimethyloxazole	0.955	61.665	140.070	other	[H]+	+	+	+
790	Isobutylpropylamine	0.950	188.802	116.144	other	[H]+	+	+	+
791	Histamine	0.945	302.632	112.087	other	[H]+	+	+	+
792	1-Nitroheptane	0.945	37.014	146.117	other	[H]+	+	+	+
793	O-Acetylethanolamine	0.943	365.994	104.071	other	[H]+	+	+	+
794	Metenamine	0.942	305.217	141.102	other	[H]+	+	+	+
795	2-Methylbutylamine	0.936	264.735	88.113	other	[H]+	+	+	+
796	Ethylbenzene	0.931	34.995	107.086	other	[H]+	+	+	+
797	N,N-Dimethylaniline	0.931	184.003	122.097	other	[H]+	+	+	+
798	3-Methyl-1-butylamine	0.931	205.542	88.113	other	[H]+	+	+	+
799	L-Carnitine	0.926	324.078	162.112	other	[H]+	+	+	+
800	2-(4-Methyl-1,3-pentadienyl)anthraquinone	0.920	202.718	289.125	other	[H]+	+	+	+
801	Nandrolone	0.914	56.180	275.200	other	[H]+	+	+	+
802	Dinoseb acetate	0.901	397.418	283.090	other	[H]+	+	+	+
803	Sulfamethoxazole N4-hydroxylamine	0.898	83.156	270.052	other	[H]+	+	+	+
804	Annoglabasin A	0.890	36.819	391.245	other	[H]+	+	-	-
805	Triethanolamine	0.890	151.663	150.112	other	[H]+	+	+	+
806	Dukunolide C	0.888	489.688	541.173	other	[H]+	+	+	+
807	Squamolone	0.887	383.699	129.066	other	[H]+	+	+	+
808	7-(1,3-Cyclohexadienyl)-5-hydroxy-2,6-dimethyl-2-hepten-4-one	0.875	120.083	235.169	other	[H]+	+	+	+
809	Carnosine	0.873	363.807	227.113	other	[H]+	+	+	+
810	Serotonin	0.872	140.136	177.102	other	[H]+	+	+	+
811	Leontogenin	0.867	32.949	447.311	other	[H]+	+	+	+
812	Pentanenitrile	0.865	410.756	84.081	other	[H]+	+	+	+
813	2-Methyl-1-hydroxybutyl-ThPP	0.863	83.828	511.121	other	[H]+	-	+	+

814	1-Methyl-1,3-cyclohexadiene	0.863	221.665	95.086	other	[H]+	+	+	+
815	Corticosterone	0.862	47.057	347.222	other	[H]+	+	+	-
816	3-O-Methylniveusin A	0.858	138.785	409.183	other	[H]+	+	+	+
817	Adrenosterone	0.854	199.371	301.179	other	[H]+	+	+	+
818	1-Cyano-2-hydroxy-3-butene	0.840	424.858	98.060	other	[H]+	+	+	+
819	Caffeine	0.832	242.160	195.087	other	[H]+	+	+	+
820	6,7-Dimethyl-8-(1-D-ribityl)lumazine	0.824	384.333	327.127	other	[H]+	+	+	-
821	1-Isothiocyanato-7-(methylthio)heptane	0.819	420.473	204.087	other	[H]+	+	+	+
822	Allixin	0.806	56.300	227.127	other	[H]+	+	+	+
823	22-Acetylpriverogenin B	0.798	32.194	517.388	other	[H]+	+	+	+
824	2-Acetylpyrrolidine	0.795	127.004	114.092	other	[H]+	+	+	+
825	4-Acetyl-3-methylpyridine	0.791	231.457	136.076	other	[H]+	+	+	+
826	Desloratadine	0.787	158.624	311.127	other	[H]+	+	+	+
827	Momordin I	0.786	245.646	765.442	other	[H]+	+	+	+
828	8-Hexanoylneosolaniol	0.783	398.337	481.240	other	[H]+	+	+	+
829	Sterebin D	0.781	199.265	295.227	other	[H]+	+	-	-
830	2-Phenylacetamide	0.774	83.512	136.076	other	[H]+	+	+	+
831	6-Chloro-N-(1-methylethyl)-1,3,5-triazine-2,4-diamine	0.773	285.089	188.070	other	[H]+	+	+	+
832	Glucopyranosylmoranoline	0.772	246.063	326.147	other	[H]+	+	+	+
833	gamma-Calacorene	0.768	153.588	201.164	other	[H]+	+	+	+
834	Cyclotetradecane	0.765	220.481	227.175	other	[H]+	-	+	+
835	3-Isovalidene-3alpha,4-dihydrophthalide	0.763	177.831	205.122	other	[H]+	+	+	+
836	9-(beta-D-Ribofuranosyl)zeatin	0.762	136.573	352.160	other	[H]+	-	-	+
837	Dihydrotestosterone	0.756	31.617	291.231	other	[H]+	+	+	+
838	N-Acetyl-2,3-dihydro-1H-pyrrole	0.750	537.665	112.076	other	[H]+	+	+	+
839	5-(2-Furanyl)-3,4-dihydro-2H-pyrrole	0.746	103.907	136.076	other	[H]+	+	+	+
840	L-Furosine	0.746	447.160	255.133	other	[H]+	+	+	+
841	O-Methylsomniferine	0.739	455.655	623.278	other	[H]+	-	+	-
842	N'-Hydroxymethylnorcotinine	0.734	64.419	193.097	other	[H]+	+	+	-
843	Pregnanetriol	0.733	36.989	337.273	other	[H]+	+	+	+
844	Pterosin O	0.732	108.710	233.149	other	[H]+	+	+	-
845	Quinceoxepine	0.732	36.873	179.143	other	[H]+	+	+	+
846	Ustiloxin D	0.730	493.107	495.242	other	[H]+	+	-	-
847	beta-Sitosterol	0.722	33.703	397.382	other	[H]+	+	+	+
848	Pyrrolidine	0.713	219.006	72.081	other	[H]+	+	+	+
849	Alitame	0.706	375.517	332.166	other	[H]+	-	-	+
850	Ipomeabisfuran	0.699	105.992	247.132	other	[H]+	+	+	+
851	Bisacurone epoxide	0.697	57.045	269.174	other	[H]+	+	+	+
852	Sandoricin	0.696	467.253	589.256	other	[H]+	-	+	-

853	7a-Hydroxy-cholestene-3-one	0.696	33.351	401.341	other	[H]+	+	+	+
854	Tetrahydrodeoxycorticosterone	0.696	183.239	335.257	other	[H]+	+	+	+
855	Mulberrofuran E	0.691	492.949	633.246	other	[H]+	+	+	+
856	Kyotorphin	0.682	372.296	338.182	other	[H]+	+	+	+
857	Eremopetasinorone A	0.676	155.032	207.138	other	[H]+	+	+	+
858	Questinol	0.672	132.959	301.070	other	[H]+	+	+	+
859	Methoxypyrazine	0.668	185.179	111.055	other	[H]+	+	+	+
860	Porphobilinogen	0.662	309.847	227.102	other	[H]+	+	+	+
861	N-Nitroso-pyrrolidine	0.624	358.758	101.071	other	[H]+	+	+	+
862	N'-Hydroxyneosaxitoxin	0.608	303.192	332.134	other	[H]+	+	+	+
863	Salsolinol	0.606	36.878	180.102	other	[H]+	+	+	+
864	6-Hydroxydopamine	0.604	60.781	170.081	other	[H]+	+	+	+
865	1-Pyrroline	0.999	331.963	70.066	other	[H]+	+	+	+
866	2,5-Dihydro-2,4-dimethyloxazole	0.996	41.278	100.076	other	[H]+	+	+	+
867	1,3-Dihydro-(2H)-indol-2-one	0.994	249.072	134.060	other	[H]+	+	+	+
868	Testosterone	0.885	33.281	289.216	other	[H]+	+	+	+
869	Clionasterol	0.862	76.645	397.382	other	[H]+	+	+	-
870	Theaspirone A	0.710	197.130	209.153	other	[H]+	+	+	+
871	Cyclocalopin D	0.806	361.359	517.190	other	[H]+	+	-	-
872	4,4alpha,5,6-Tetrahydro-7-methyl-2(3H)-naphthalenone	0.954	121.903	163.112	other	[H]+	+	+	+
873	Niacinamide	0.995	60.806	123.055	other	[H]+	+	+	+
874	(2E)-Decenoyl-ACP	0.997	330.615	128.070	other	[H]-	+	+	+
875	Isobutyrylglycine	0.989	231.568	144.065	other	[H]-	-	-	+
876	2,4,6-trihydroxy-2-[(4-hydroxyphenyl)methyl]-2,3-dihydro-1-benzofuran-3-one	0.988	293.589	271.060	other	[H]+	+	+	+
877	Aflatoxin G2	0.879	121.020	331.080	other	[H]-	+	+	+
878	Diplodiatoxin	0.645	104.406	309.205	other	[H]-	+	+	+
879	Benzofuran	0.536	325.574	119.049	other	[H]-	+	+	+
880	(±)-Conen	0.629	429.360	305.076	other	[H]-	+	+	+
881	1-(beta-D-Ribofuranosyl)-1,4-dihydronicotinamide	0.590	176.033	257.113	other	[H]+	-	+	+
882	3,11,12-Trihydroxy-1(10)-spirovetiven-2-one	0.561	37.691	269.174	other	[H]-	+	+	+
883	Phosphoribosyl formamidocarboxamide	0.595	418.300	367.062	other	[H]+	+	+	+
884	2-(3,4-dihydroxyphenyl)-5,7-dihydroxy-6-[3,4,5-trihydroxy-6-(hydroxymethyl)oxan-2-yl]-4H-chromen-4-one	0.675	278.916	449.107	other	[H]+	+	+	+
885	Capsiate	0.838	58.911	307.189	other	[H]+	+	+	-
886	2-Methoxy-3-methylpyrazine	0.796	54.950	125.071	other	[H]+	+	+	-
887	Hericrin IV	0.391	180.397	171.065	other	[H]+	+	+	-

888	Beta-Carboline	0.918	50.130	169.076	other	[H]+	+	+	+
889	Oroselone	0.752	160.403	227.070	other	[H]+	+	+	-

Note: score represents the matching degree of metabolites and substances in the database; rt is retention time (s); m/z is mass-to-nucleus ratio; Classification is the classification of metabolites; Ion mode is the ion mode of metabolites scanned in mass spectrometry; "+" means containing the metabolite, "-" means less than containing the metabolite.

Table S2 List of differential metabolites between unprocessed black soybeans and cooked black soybeans

	Name	score	rt	mz	MEAN r	MEAN p	VIP	P-VALUE	Q-VALUE	FOLD CHANGE	LOG_FOLDCHANGE
1	Methionyl-Valine	0.528	226.046	249.126	3.36E-06	6.61E-08	2.763	0.0488	0.0222	50.882	5.669
2	L-Asparagine	0.993	398.701	131.045	1.02E-04	2.91E-06	3.005	0.0017	0.0052	35.114	5.134
3	Tyrosyl-Tryptophan	0.768	229.66	368.160	4.31E-06	3.97E-07	2.206	0.0010	0.0015	10.860	3.441
4	Histidinyl-Methionine	0.765	323.48	287.117	1.82E-05	1.93E-06	2.096	0.0155	0.0078	9.444	3.239
5	Valyl-Tryptophan	0.571	217.277	304.165	5.51E-06	8.89E-07	1.999	0.0016	0.0015	6.195	2.631
6	Glycitein	0.939	260.086	283.061	3.14E-03	5.68E-04	1.765	0.0299	0.0131	5.526	2.466
7	Phenylalanyl-Gamma-glutamate	0.710	283.277	294.145	4.27E-06	8.77E-07	1.701	0.0306	0.0146	4.863	2.282
8	Valyl-Threonine	0.781	242.901	219.134	2.84E-04	6.61E-05	1.654	0.0073	0.0039	4.298	2.104
9	Leucyl-Tyrosine	0.932	231.431	295.165	3.26E-05	8.07E-06	1.652	0.0001	0.0011	4.035	2.013
10	Prolyl-Glutamine	0.845	367.5175	244.129	5.44E-06	1.61E-06	1.532	0.0020	0.0015	3.389	1.761
11	Cysteinyl-Proline	0.634	235.104	219.079	6.52E-04	2.16E-04	1.395	0.0255	0.0123	3.014	1.592
12	Alanyl-Leucine	0.988	253.423	203.139	6.17E-05	2.19E-05	1.447	0.0450	0.0206	2.823	1.497
13	N-Acetyl-L-methionine	0.938	224.008	190.053	9.09E-06	3.25E-06	1.350	0.0437	0.0183	2.800	1.485
14	Tyrosyl-Threonine	0.820	281.5605	283.128	8.92E-06	3.25E-06	1.387	0.0035	0.0020	2.746	1.457
15	D-Glutamine	0.990	395.613	147.076	3.65E-04	1.44E-04	1.342	0.0008	0.0015	2.540	1.345
16	Prolyl-Lysine	0.684	451.9705	244.165	1.07E-05	4.52E-06	1.203	0.0345	0.0163	2.364	1.241
17	Isoleucyl-Serine	0.984	281.5655	219.133	1.16E-04	5.20E-05	1.206	0.0098	0.0052	2.239	1.163
18	Isoleucyl-Tryptophan	0.643	210.846	318.181	4.93E-05	2.21E-05	1.234	0.0011	0.0015	2.230	1.157
19	L-Glutamic acid	0.993	417.911	146.045	3.09E-03	1.61E-03	1.086	0.0015	0.0050	1.917	0.939
20	L-Proline	0.999	333.297	114.055	3.17E-04	1.72E-04	1.066	0.0017	0.0052	1.841	0.880
21	N-Carboxyethyl-g-aminobutyric acid	0.702	288.1005	174.076	5.72E-04	3.16E-04	1.032	0.0029	0.0060	1.811	0.857
22	4',8-Dimethylgossypetin 3-glucoside	0.498	180.767	509.129	1.10E-05	6.61E-08	3.177	0.0099	0.0052	166.152	7.376
23	Hydroxytyrosol 1-O-glucoside	0.841	204.0725	317.126	9.47E-06	3.39E-07	2.607	0.0000	0.0010	27.964	4.805
24	Threonic acid	0.915	334.521	135.029	4.46E-04	1.11E-04	1.601	0.0167	0.0078	4.033	2.012
25	Chrysoeriol 7-O-(6''-malonyl-glucoside)	0.590	236.0205	549.123	4.26E-05	1.29E-05	1.495	0.0022	0.0015	3.294	1.720
26	Gluconolactone	0.653	123.32	177.039	1.96E-04	6.55E-05	1.402	0.0028	0.0059	2.989	1.580
27	4-Methoxybenzyl glucoside	0.469	141.8605	301.129	4.21E-05	1.62E-05	1.342	0.0074	0.0040	2.600	1.379
28	Gluconic acid	0.913	396.52	195.050	2.46E-03	9.73E-04	1.294	0.0029	0.0060	2.528	1.338
29	Rhamnose	0.782	151.2155	163.060	1.60E-04	6.46E-05	1.300	0.0001	0.0009	2.479	1.310
30	L-Iditol	0.954	309.838	181.071	1.32E-03	6.34E-04	1.151	0.0008	0.0039	2.074	1.052
31	L-Arabitol	0.993	97.7975	151.060	1.81E-04	9.47E-05	1.088	0.0007	0.0036	1.906	0.931
32	Deacetylisovaltrate	0.763	396.479	381.188	1.42E-05	6.61E-08	3.253	0.0181	0.0091	214.890	7.747
33	Methylsuccinic acid	0.984	374.444	131.034	5.79E-05	1.29E-05	1.899	0.0109	0.0071	4.502	2.170
34	Leucinic acid	0.998	137.152	131.070	3.05E-03	7.95E-04	1.575	0.0096	0.0070	3.832	1.938
35	Traumatic acid	0.862	241.441	227.128	3.63E-04	9.83E-05	1.562	0.0000	0.0004	3.697	1.886

36	20-Hydroxy-PGE2	0.998	107.796	351.213	6.15E-06	2.34E-06	1.336	0.0138	0.0071	2.625	1.392
37	3,3-Dimethylglutaric acid	0.680	65.1768	159.065	3.07E-04	1.29E-04	1.229	0.0114	0.0071	2.385	1.254
38	L-Acetylcarnitine	0.755	406.107	204.123	2.89E-04	1.38E-04	1.204	0.0001	0.0010	2.091	1.065
39	Itaconic acid	0.973	66.82765	129.018	1.22E-03	6.06E-04	1.130	0.0008	0.0039	2.018	1.013
40	9,10-DHOME	0.948	65.54665	313.238	1.08E-04	5.57E-05	1.073	0.0058	0.0067	1.942	0.958
41	(2'E,4'Z,7'Z,8E)-Colnelenic acid	0.703	63.3459	293.211	1.41E-05	4.86E-06	1.593	0.0229	0.0112	2.906	1.539
42	Panaquinquecol 1	0.653	203.871	293.211	1.32E-06	1.53E-07	2.097	0.0041	0.0023	8.653	3.113
43	Glycerol 3-phosphate	0.849	453.589	171.005	9.49E-05	9.58E-08	3.591	0.0016	0.0051	990.184	9.952
44	5-Heptyltetrahydro-2-oxo-3-furan carboxylic acid	0.525	58.1361	229.143	1.41E-05	5.47E-06	1.344	0.0233	0.0114	2.577	1.365
45	Daidzein	0.999	260.581	253.050	8.78E-03	3.80E-03	1.247	0.0001	0.0012	2.312	1.209
46	5,7-Dihydroxy-2',6-dimethoxyiso flavone 7-rhamnoside	0.541	190.466	461.144	5.88E-04	2.06E-04	1.427	0.0138	0.0071	2.846	1.509
47	Luteolin 7-galactoside	0.944	241.71	449.107	4.30E-03	5.76E-05	2.921	0.0075	0.0040	74.630	6.222
48	Cosmosiin	0.996	201.8385	433.112	3.31E-03	6.75E-05	2.771	0.0121	0.0063	49.088	5.617
49	Trilobatin	0.865	202.976	437.143	1.22E-05	3.78E-07	2.674	0.0083	0.0044	32.225	5.010
50	Luteolin 4'-glucoside	0.971	523.336	449.107	1.42E-03	7.40E-05	2.410	0.0497	0.0226	19.161	4.260
51	Luteolin	0.994	521.279	287.054	1.53E-04	9.65E-06	2.364	0.0101	0.0053	15.882	3.989
52	Peonidin-3-glucoside	0.990	153.4245	463.123	3.02E-04	2.66E-05	2.199	0.0001	0.0011	11.320	3.501
53	Citrusinine II	0.448	60.7817	288.086	5.56E-04	5.52E-05	2.132	0.0006	0.0014	10.071	3.332
54	Isorhamnetin	0.981	192.786	317.066	1.49E-04	1.85E-05	2.030	0.0000	0.0004	8.047	3.008
55	Sideritiflavone	0.989	102.9545	361.091	2.97E-05	4.48E-06	1.928	0.0001	0.0012	6.632	2.730
56	5,7-dihydroxy-2-(4-hydroxy-3-m ethoxyphenyl)-3-{[3,4,5-trihydro xy-6-(hydroxymethyl)oxan-2-yl]o xy}-4H-chromen-4-one	0.782	179.7965	477.104	8.49E-05	2.03E-05	1.622	0.0014	0.0049	4.180	2.064
57	(-)-Epiafzelechin	0.962	58.12795	273.076	3.78E-03	9.48E-04	1.605	0.0001	0.0009	3.988	1.996
58	Phlorizin	0.351	225.4325	435.130	1.22E-04	5.31E-05	1.242	0.0002	0.0016	2.297	1.200
59	Norizalpinin	0.754	294.63	269.045	1.00E-04	4.97E-05	1.127	0.0015	0.0050	2.019	1.014
60	Carthamone	0.959	475.95	449.107	8.95E-04	5.01E-05	2.404	0.0003	0.0014	17.878	4.160
61	3,4-Dihydroxyhydrocinnamic acid	0.842	96.2775	181.050	2.21E-04	8.88E-05	1.254	0.0119	0.0071	2.486	1.314
62	Marmesin	0.897	136.361	245.082	1.01E-04	2.87E-05	1.513	0.0024	0.0057	3.523	1.817
63	Protocatechuic acid	0.812	39.59395	153.018	3.19E-03	2.94E-04	2.076	0.0405	0.0172	10.864	3.441
64	Phenol	0.866	325.5565	95.050	9.66E-06	1.39E-06	2.384	0.0052	0.0029	6.946	2.796
65	Acetaminophen	0.937	102.583	152.070	4.21E-04	1.45E-04	1.432	0.0027	0.0015	2.912	1.542
66	Gallic acid	0.649	32.8544	169.013	2.62E-04	9.42E-05	1.381	0.0001	0.0009	2.787	1.479
67	benzene-1,2,4-triol	0.967	392.763	125.023	2.17E-04	1.25E-04	1.003	0.0009	0.0042	1.739	0.798
68	Inosine	0.999	237.5	269.087	7.63E-06	2.43E-06	1.478	0.0083	0.0044	3.145	1.653
69	Cytidine 2'-phosphate	0.997	456.9355	324.059	4.29E-05	1.41E-05	1.449	0.0058	0.0032	3.039	1.604
70	Cytidine	0.985	261.061	242.078	1.55E-05	6.96E-06	1.174	0.0086	0.0070	2.222	1.152
71	2'-O-Methyladenosine	0.997	99.726	282.120	1.60E-04	7.37E-05	1.228	0.0004	0.0014	2.178	1.123
72	Ribitol	0.854	248.9835	151.060	3.88E-04	1.91E-04	1.142	0.0005	0.0029	2.035	1.025
73	Cytarabine	1.000	260.517	244.093	6.75E-05	3.45E-05	1.124	0.0024	0.0015	1.959	0.970

74	5'-Methylthioadenosine	0.975	93.3963	298.096	3.81E-04	2.15E-04	1.048	0.0006	0.0014	1.769	0.823
75	Thymine	0.918	94.2766	127.050	1.51E-05	6.61E-08	3.272	0.0123	0.0063	227.896	7.832
76	Malvidin 3-glucoside	0.839	100.318	493.133	1.34E-04	3.70E-05	1.590	0.0000	0.0009	3.616	1.854
77	Diferuloylputrescine	0.808	56.70395	441.201	4.94E-05	2.12E-07	3.385	0.0031	0.0018	232.897	7.864
78	2-(3,4-dihydroxyphenyl)-5,7-dihydroxy-6-[3,4,5-trihydroxy-6-(hydroxymethyl)oxan-2-yl]-4H-chromen-4-one	0.675	278.916	449.107	3.50E-04	4.83E-06	2.921	0.0223	0.0109	72.490	6.180
79	Capsiate	0.838	58.9105	307.189	3.10E-06	6.61E-08	2.758	0.0027	0.0016	46.922	5.552
80	Bisacurone epoxide	0.697	57.0446	269.174	3.39E-05	2.90E-06	2.222	0.0001	0.0011	11.695	3.548
81	Benzofuran	0.536	325.574	119.049	1.20E-05	1.72E-06	2.431	0.0047	0.0026	6.967	2.800
82	Aflatoxin G2	0.879	121.02	331.080	1.50E-04	2.54E-05	1.870	0.0000	0.0005	5.901	2.561
83	Diplodiatoxin	0.645	104.4055	309.205	1.26E-05	2.33E-06	1.826	0.0024	0.0015	5.393	2.431
84	(-)-Gossypol	0.856	465.04	517.189	2.30E-06	4.70E-07	1.781	0.0073	0.0069	4.889	2.290
85	2-(3,4-dihydroxyphenyl)-3,5-dihydroxy-7-methoxy-4H-chromen-4-one	0.880	51.97445	315.051	1.69E-04	3.87E-05	1.637	0.0035	0.0062	4.367	2.127
86	(±)-Conen	0.629	429.36	305.076	4.07E-05	1.59E-05	1.339	0.0014	0.0015	2.557	1.355
87	Pregnanetriol	0.733	36.9893	337.273	7.17E-05	2.83E-05	1.262	0.0445	0.0205	2.535	1.342
88	Sulfamethoxazole N4-hydroxylamine	0.898	83.1563	270.052	1.23E-05	4.87E-06	1.286	0.0161	0.0081	2.519	1.333
89	Dopamine	0.447	310.7165	299.077	4.91E-05	2.05E-05	1.230	0.0133	0.0072	2.398	1.262
90	2,4,6-trihydroxy-2-[(4-hydroxyphenyl)methyl]-2,3-dihydro-1-benzofuran-3-one	0.988	293.5885	271.060	1.33E-04	5.74E-05	1.272	0.0005	0.0014	2.313	1.210
91	3,11,12-Trihydroxy-1(10)-spirovetiven-2-one	0.561	37.69105	269.174	1.97E-05	9.40E-06	1.190	0.0023	0.0015	2.092	1.065
92	2-Methoxy-3-methylpyrazine	0.796	54.95045	125.071	3.06E-04	1.49E-04	1.169	0.0021	0.0015	2.056	1.040
93	Herierin IV	0.391	180.3965	171.065	2.57E-05	1.30E-05	1.157	0.0000	0.0010	1.978	0.984
94	1-Pyrroline	0.999	331.963	70.066	4.69E-06	2.45E-06	1.099	0.0058	0.0032	1.915	0.938
95	2-Hydroxyethanesulfonate	0.969	149.026	124.990	2.35E-04	1.34E-04	1.012	0.0007	0.0036	1.747	0.805
96	Questinol	0.672	132.959	301.070	3.77E-04	2.22E-04	1.014	0.0007	0.0014	1.698	0.764
97	Vanillin 3-(L-menthoxy)propane-1,2-diol acetal	0.626	50.2505	365.231	5.62E-06	6.14E-07	2.116	0.0055	0.0030	9.160	3.195
98	3-(4-Isopropylphenyl)propanal	0.780	62.4195	177.127	9.20E-06	3.28E-06	1.424	0.0001	0.0011	2.799	1.485
99	Imidazole-4-acetaldehyde	0.751	63.761	111.055	4.21E-03	1.65E-03	1.348	0.0012	0.0015	2.555	1.353
100	3-Isoxazolidinone	0.999	397.408	88.040	2.49E-05	2.30E-06	2.251	0.0007	0.0014	10.837	3.438
101	Xanthine	0.965	233.541	151.025	3.03E-05	3.21E-06	2.040	0.0148	0.0072	9.431	3.237
102	Hypoxanthine	1.000	236.753	137.046	6.03E-05	1.78E-05	1.786	0.0416	0.0193	3.388	1.760
103	Imidazoleacetic acid	0.894	95.2574	125.034	1.81E-04	7.47E-05	1.269	0.0217	0.0099	2.426	1.279
104	Fagomine	0.891	321.161	148.097	4.09E-05	2.42E-05	1.005	0.0006	0.0014	1.686	0.754
105	2-Aminoheptanedioic acid	0.565	285.5375	176.091	3.04E-04	4.38E-05	1.955	0.0001	0.0013	6.941	2.795
106	Syringic acid	0.674	85.0591	197.045	3.38E-05	8.87E-06	1.571	0.0005	0.0031	3.815	1.932

107	Homovanillic acid	0.881	51.6393	181.050	1.21E-03	4.24E-04	1.371	0.0043	0.0064	2.852	1.512
108	Phthalic acid	0.643	132.1145	165.018	2.97E-04	1.10E-04	1.332	0.0102	0.0070	2.701	1.433
109	[8]-Paradyl acetate	0.637	36.838	349.237	2.29E-05	8.50E-06	1.371	0.0329	0.0156	2.697	1.431
110	Oxoglutaric acid	0.989	388.681	145.013	1.93E-04	7.97E-05	1.241	0.0060	0.0067	2.420	1.275
111	Phenylglyoxylic acid	0.550	56.1983	149.023	3.95E-05	1.64E-05	1.225	0.0182	0.0084	2.415	1.272
112	L-trans-alpha-Amino-2-carboxycyclopropaneacetic acid	0.934	428.155	160.060	1.34E-04	6.50E-05	1.181	0.0005	0.0014	2.055	1.039
113	L-Dihydroorotic acid	1.000	321.3825	157.024	6.08E-05	3.29E-05	1.067	0.0001	0.0009	1.846	0.884
114	Pyrrolidonecarboxylic acid	1.000	417.611	130.050	1.38E-03	7.99E-04	1.029	0.0005	0.0014	1.725	0.787
115	Gerberinol	0.582	190.484	365.102	3.11E-05	6.59E-06	1.733	0.0016	0.0015	4.724	2.240
116	Perilloside C	0.794	441.3915	317.193	8.43E-06	4.94E-06	1.001	0.0047	0.0026	1.707	0.771
117	3,5,7-trihydroxy-2-(4-hydroxyphenyl)-3,4-dihydro-2H-1-benzopyran-4-one	0.521	58.7586	287.056	2.26E-03	2.71E-04	1.992	0.0001	0.0009	8.363	3.064
118	3,4-Dihydro-2H-1-benzopyran-2-one	0.513	280.69	149.060	9.43E-05	3.91E-05	1.291	0.0030	0.0017	2.410	1.269
119	Pyridoxine	0.961	102.47	170.081	2.39E-03	8.45E-04	1.428	0.0001	0.0011	2.829	1.500
120	Thiamine monophosphate	0.952	514.422	345.078	3.65E-05	9.54E-06	1.616	0.0043	0.0024	3.825	1.936
121	Methyl vanillate	0.862	74.22	181.050	5.20E-04	1.64E-04	1.459	0.0004	0.0028	3.167	1.663
122	2-Carboxy-4-dodecanolide	0.614	207.3505	243.159	4.86E-05	2.33E-05	1.195	0.0002	0.0014	2.086	1.060
123	3-Methylbutyl 2-furanbutanoate	0.657	208.097	225.148	3.70E-05	1.91E-05	1.136	0.0004	0.0014	1.939	0.955
124	Dihydrolipoate	0.540	67.353	207.050	3.84E-04	2.16E-04	1.025	0.0018	0.0053	1.780	0.832
125	Dukunolide B	0.977	353.105	499.157	4.82E-05	2.74E-05	1.008	0.0107	0.0056	1.760	0.815
126	N-a-Acetyl-L-arginine	0.853	392.121	217.129	2.27E-04	4.58E-04	1.173	0.0054	0.0030	0.496	-1.011
127	Epsilon-(gamma-Glutamyl)-lysine	0.789	477.674	274.140	6.63E-07	1.59E-06	1.221	0.0111	0.0071	0.416	-1.264
128	Serylasparagine	0.491	377.862	220.105	3.16E-05	8.65E-05	1.390	0.0027	0.0015	0.365	-1.454
129	Isoleucyl-Methionine	0.799	213.612	263.142	4.09E-06	1.15E-05	1.392	0.0096	0.0050	0.356	-1.491
130	Tyrosyl-Glutamine	0.709	86.1295	310.139	8.84E-06	2.86E-05	1.505	0.0015	0.0015	0.309	-1.693
131	Histidinyl-Asparagine	0.716	394.7845	270.119	2.47E-06	8.09E-06	1.466	0.0151	0.0077	0.305	-1.712
132	Asparaginyl-Arginine	0.704	472.179	289.161	4.48E-07	3.39E-06	2.222	0.0192	0.0095	0.132	-2.918
133	Gamma-Aminobutyryl-lysine	0.402	99.53	232.165	1.41E-07	1.26E-06	2.072	0.0442	0.0203	0.112	-3.162
134	Prolyl-Asparagine	0.907	350.807	230.113	4.28E-07	4.39E-06	2.181	0.0023	0.0015	0.098	-3.356
135	Prolyl-Gamma-glutamate	0.579	448.9275	244.140	5.23E-07	6.35E-06	2.230	0.0000	0.0009	0.082	-3.603
136	L-Tryptophan	0.526	74.8669	203.082	1.08E-05	2.49E-04	2.412	0.0136	0.0072	0.043	-4.526
137	Phenylalanyl-Methionine	0.871	208.384	297.126	5.38E-08	4.11E-06	2.913	0.0318	0.0151	0.013	-6.258
138	Lysyl-Methionine	0.580	403.764	278.153	5.02E-07	7.25E-06	2.349	0.0007	0.0014	0.069	-3.852
139	Stachyose	0.785	516.787	689.210	9.65E-05	1.69E-04	1.011	0.0120	0.0062	0.569	-0.813
140	Goyaglycoside e	0.483	313.075	781.474	6.09E-06	1.47E-05	1.307	0.0005	0.0014	0.414	-1.271
141	1-Deoxy-D-glucitol	0.574	127.8345	167.092	3.62E-04	3.03E-03	2.052	0.0000	0.0002	0.119	-3.069
142	(R)-3-Hydroxy-tetradecanoic acid	0.684	71.29765	243.196	1.07E-05	3.56E-05	1.404	0.0292	0.0128	0.301	-1.733
143	4,8 Dimethylnonanoyl carnitine	0.886	64.1748	330.264	1.72E-05	7.34E-05	1.685	0.0183	0.0091	0.234	-2.097
144	PS(18:0/22:6(4Z,7Z,10Z,13Z,16Z,19Z))	0.815	178.5995	836.541	2.87E-07	2.40E-05	3.120	0.0219	0.0108	0.012	-6.386

145	Pelargonic acid	0.870	45.8406	157.122	7.42E-08	6.31E-05	3.546	0.0145	0.0072	0.001	-9.731
146	LysoPE(20:5(5Z,8Z,11Z,14Z,17Z)/0:0)	0.850	228.7385	500.275	3.24E-06	8.70E-06	1.336	0.0442	0.0203	0.373	-1.424
147	gamma-Aminobutyric acid	0.980	170.5685	104.071	5.56E-05	2.52E-04	1.689	0.0102	0.0054	0.221	-2.180
148	Alpha-Linolenic acid	1.000	50.31515	277.217	7.37E-04	1.49E-03	1.094	0.0129	0.0072	0.496	-1.012
149	13-OxoODE	0.989	38.0511	293.212	1.41E-04	3.10E-04	1.200	0.0008	0.0039	0.454	-1.138
150	Isopentyl beta-D-glucoside	0.727	159.254	251.150	1.29E-06	1.14E-05	2.150	0.0003	0.0014	0.113	-3.151
151	LysoPE(18:3(6Z,9Z,12Z)/0:0)	0.922	230.628	476.277	5.34E-05	9.40E-05	1.007	0.0150	0.0076	0.568	-0.817
152	1-Palmitoylglycerophosphoinositol	0.884	273.602	573.302	4.50E-05	9.54E-05	1.126	0.0406	0.0188	0.472	-1.084
153	6''-O-Malonyldaidzin	0.723	214.586	503.119	3.78E-04	8.01E-04	1.213	0.0062	0.0034	0.472	-1.083
154	Genistein 5-glucoside	0.988	126.809	433.112	9.47E-03	2.12E-02	1.229	0.0401	0.0186	0.446	-1.166
155	6''-O-Acetylglycitin	0.946	62.5232	489.139	7.32E-05	3.10E-04	1.689	0.0000	0.0004	0.236	-2.082
156	6''-O-Acetylgenistin	0.969	161.269	475.123	5.61E-05	3.99E-04	1.970	0.0000	0.0003	0.141	-2.829
157	6''-O-Acetyldaidzin	0.964	61.6527	459.129	2.22E-04	1.93E-03	2.067	0.0000	0.0009	0.115	-3.118
158	6''-O-Malonylwistin	0.829	91.7124	547.145	5.38E-08	9.16E-06	3.186	0.0081	0.0043	0.006	-7.412
159	Naringenin	0.955	118.185	271.061	2.98E-04	5.44E-04	1.006	0.0150	0.0072	0.549	-0.866
160	Daidzin	0.937	163.2115	415.102	1.97E-03	4.46E-03	1.230	0.0002	0.0016	0.442	-1.178
161	Liquiritin	0.713	164.28	417.119	6.57E-05	1.49E-04	1.204	0.0061	0.0067	0.441	-1.181
162	Soyasaponin IV	0.587	257.04	767.458	1.25E-05	2.87E-05	1.242	0.0052	0.0029	0.436	-1.198
163	Diosmetin	0.924	114.005	299.055	1.88E-05	7.63E-05	1.571	0.0393	0.0167	0.247	-2.019
164	Sandosaponin B	0.343	317.124	957.506	1.31E-05	5.42E-05	1.642	0.0076	0.0041	0.242	-2.046
165	5,7-dihydroxy-2-phenyl-6-[3,4,5-trihydroxy-6-(hydroxymethyl)oxan-2-yl]-8-(3,4,5-trihydroxyoxan-2-yl)-4H-chromen-4-one	0.408	81.57355	547.145	4.37E-05	2.21E-04	1.729	0.0129	0.0072	0.197	-2.340
166	Cyanidin 3-(4-acetylglucoside)	0.960	117.3335	491.118	1.15E-05	1.53E-04	2.259	0.0044	0.0024	0.075	-3.727
167	Apigenin 7-O-(6''-O-acetylglucoside)	0.943	107.416	475.124	2.06E-06	3.70E-05	2.386	0.0176	0.0088	0.056	-4.169
168	Feruloyl-2-hydroxyputrescine	1.000	152.309	281.149	4.88E-06	5.37E-05	2.247	0.0003	0.0014	0.091	-3.459
169	3,3',4'5-Tetrahydroxystilbene	0.933	116.753	243.066	1.42E-05	5.35E-05	1.554	0.0019	0.0054	0.266	-1.912
170	Maltol	1.000	69.91055	127.039	9.45E-05	9.84E-04	2.154	0.0000	0.0001	0.096	-3.380
171	Phlorin	0.994	164.109	289.091	4.75E-04	3.23E-03	1.948	0.0000	0.0001	0.147	-2.766
172	3-(4-Hydroxyphenyl)-1-propanol	0.509	34.18005	153.091	6.49E-06	1.29E-05	1.162	0.0000	0.0008	0.505	-0.987
173	Diacetone alcohol	0.980	72.8391	115.075	6.85E-05	2.62E-04	1.891	0.0053	0.0066	0.261	-1.937
174	Abscisic acid	0.849	83.3141	263.128	1.05E-05	2.06E-05	1.072	0.0486	0.0201	0.509	-0.974
175	FAPy-adenine	0.951	188.8795	154.072	1.82E-05	8.01E-05	1.710	0.0000	0.0006	0.227	-2.142
176	Dihydrouracil	0.831	60.7731	115.050	9.30E-06	6.83E-05	1.985	0.0000	0.0009	0.136	-2.877
177	5-(2-Hydroxyethyl)-4-methylthiazole	0.985	48.16975	144.048	2.16E-05	4.24E-05	1.056	0.0420	0.0194	0.510	-0.972
178	Ethylbenzene	0.931	34.995	107.086	1.35E-05	2.78E-05	1.134	0.0129	0.0066	0.485	-1.045
179	Dinoseb acetate	0.901	397.4175	283.090	1.35E-06	2.81E-06	1.149	0.0323	0.0153	0.480	-1.058
180	Eremopetasinorone A	0.676	155.032	207.138	4.26E-05	8.98E-05	1.203	0.0005	0.0014	0.474	-1.076
181	Beta-Carboline	0.918	50.1296	169.076	1.46E-05	3.67E-05	1.345	0.0001	0.0012	0.398	-1.327

182	Phosphoribosyl formamidocarboxamide	0.595	418.2995	367.062	8.02E-05	2.17E-04	1.349	0.0181	0.0091	0.370	-1.434
183	Oroselone	0.752	160.403	227.070	7.68E-06	2.25E-05	1.402	0.0139	0.0071	0.341	-1.553
184	7-Hydroxymethyl-12-methylbenz [a]anthracene sulfate	0.977	70.36195	353.084	1.35E-05	1.13E-04	2.028	0.0217	0.0107	0.120	-3.057
185	Niacinamide	0.995	60.8059	123.055	3.23E-04	4.82E-03	2.310	0.0068	0.0037	0.067	-3.902
186	Sebiferic acid	0.617	303.963	441.373	8.19E-05	1.76E-04	1.197	0.0041	0.0023	0.465	-1.105
187	Jujubasaponin IV	0.352	306.007	943.524	4.69E-04	1.00E-03	1.188	0.0044	0.0025	0.469	-1.092
188	Jujubasaponin VI	0.393	270.451	797.468	1.91E-04	5.26E-04	1.397	0.0006	0.0014	0.364	-1.459
189	Ganoderic acid Mi	0.761	33.2484	545.384	1.20E-06	3.69E-06	1.457	0.0035	0.0020	0.326	-1.617
190	S-Furanopetasitin	0.795	495.262	433.203	7.29E-07	2.47E-06	1.493	0.0140	0.0072	0.295	-1.764
191	Ganoderal A	0.720	34.374	437.341	2.85E-07	7.91E-06	2.733	0.0006	0.0014	0.036	-4.793
192	2'-Hydroxyacetophenone	0.993	51.0363	137.060	1.18E-05	2.25E-05	1.053	0.0297	0.0142	0.525	-0.930
193	3-[(3-Methylbutyl)nitrosoamino]-2-butanone	0.576	495.8015	187.144	4.80E-05	1.41E-04	1.451	0.0004	0.0014	0.341	-1.553
194	Malonic acid	0.817	392.763	103.002	5.72E-04	1.10E-03	1.091	0.0020	0.0054	0.518	-0.949
195	O-Phosphoethanolamine	0.921	488.856	142.026	6.95E-06	1.41E-05	1.143	0.0103	0.0054	0.492	-1.024
196	Fumaric acid	0.998	247.122	115.002	3.42E-04	7.73E-04	1.185	0.0092	0.0070	0.442	-1.177
197	Ethylparaben	0.585	45.8428	165.055	4.18E-05	3.58E-04	1.970	0.0473	0.0196	0.116	-3.102

Note: rt is the chromatographic retention time of the substance; Mass is the exact molecular weight of the metabolite; score is the match with the substance in the database; The mean r is the relative quantitative mean of the substances in the unprocessed black bean group; The mean p is the relative quantitative mean of the substances in the cooked black bean group; VIP is the variable projection importance obtained by the OPLS-DA model of the comparison in this group; P-VALUE is the P value obtained by the t-test of the comparison in this group, P value = the probability that the hypothesis is correct but rejected = the number of negative results / the total number of results, which is a test probability for the sample data; Q-VALUE is the hypothesis test statistic (P value) after correction for multiple hypothesis testing The result of , Q value = probability of being rejected but correct = number of false positive results / number of presumed positive results, which is a test probability for the inference obtained by the t test, and a re-statistic for the P value; FOLD CHANGE is the fold relationship of the substance in the comparison between the two groups of experiments; LOG_FOLDCHANGE FOLD CHANGE is the logarithm with the base 2, "-" means the relative content is low.

Table S3 List of differential metabolites between Unprocessed black soybeans and Steamed black soybeans

	Name	score	rt	mz	MEAN r	MEAN ops	VIP	P-VALUE	Q-VALUE	FOLD CHANGE	LOG_FOLDCHANGE
1	Glycitein	0.939	260.086	283.061	3.14E-03	1.26E-03	1.458	0.0156	0.0239	2.485	1.313
2	Phenylalanylproline	0.934	260.008	261.124	7.48E-06	3.37E-06	1.358	0.0076	0.0161	2.222	1.152
3	1-deoxy-1-(N6-lysino)-D-fructose	0.759	424.806	132.029	1.05E-03	3.11E-04	1.724	0.0172	0.0250	3.375	1.755
4	D-Aspartic acid	0.996	424.735	134.045	2.81E-04	6.97E-05	1.894	0.0030	0.0121	4.034	2.012
5	L-Pipecolic acid	0.993	224.347	130.083	5.37E-05	3.43E-05	1.046	0.0088	0.0267	1.564	0.645
6	D-Glutamine	0.990	395.613	147.076	3.65E-04	2.22E-04	1.136	0.0003	0.0027	1.644	0.717
7	Prolylphenylalanine	0.909	257.011	263.139	1.04E-04	6.69E-05	1.068	0.0002	0.0020	1.549	0.632
8	Prolyl-Glutamine	0.845	367.518	244.129	5.44E-06	1.68E-06	1.734	0.0019	0.0088	3.232	1.692
9	N-Acetyl-L-glutamate 5-semialdehyde	0.793	394.414	174.076	1.27E-03	8.31E-04	1.037	0.0247	0.0546	1.531	0.615
10	Valyl-Threonine	0.781	242.901	219.134	2.84E-04	6.50E-05	1.937	0.0059	0.0200	4.373	2.129
11	Histidinyl-Methionine	0.765	323.480	287.117	1.82E-05	2.87E-06	2.198	0.0023	0.0100	6.364	2.670
12	Lysinoalanine	0.747	473.379	234.144	1.18E-05	5.16E-06	1.399	0.0181	0.0437	2.295	1.198
13	gamma-Glutamylvaline	0.607	396.464	247.129	1.61E-04	1.04E-04	1.057	0.0017	0.0081	1.547	0.630
14	Threoninyl-Serine	0.585	409.705	207.101	2.28E-05	1.36E-05	1.113	0.0126	0.0341	1.668	0.738
15	Tryptophyl-Tryptophan	0.550	211.644	391.175	3.19E-05	1.78E-05	1.233	0.0002	0.0020	1.792	0.842
16	L-Iditol	0.954	309.838	181.071	1.32E-03	8.12E-04	1.095	0.0001	0.0008	1.620	0.696
17	D-Xylose	0.780	314.593	149.044	1.35E-03	7.77E-04	1.133	0.0125	0.0215	1.732	0.793
18	Gluconolactone	0.653	123.320	177.039	1.96E-04	9.52E-05	1.321	0.0029	0.0082	2.056	1.040
19	Hydroxytyrosol 1-O-glucoside	0.841	204.073	317.126	9.47E-06	1.81E-06	2.082	0.0001	0.0011	5.225	2.385
20	Chrysoeriol 7-O-(6''-malonyl-glucoside)	0.590	236.021	549.123	4.26E-05	1.98E-05	1.407	0.0005	0.0035	2.149	1.103
21	5,7-Dihydroxy-2',6-dimethoxyisoflavone 7-rhamnoside	0.541	190.466	461.144	5.88E-04	3.34E-04	1.191	0.0060	0.0201	1.761	0.816
22	4',8-Dimethylgossypetin 3-glucoside	0.498	180.767	509.129	1.10E-05	8.31E-07	2.612	0.0008	0.0051	13.201	3.723
23	Leucinic acid	0.998	137.152	131.070	3.05E-03	1.12E-03	1.569	0.0130	0.0219	2.728	1.448
24	Dihydrojasmonic acid	0.992	133.922	211.133	2.66E-06	5.53E-07	1.947	0.0258	0.0292	4.817	2.268
25	9,10-Epoxyoctadecenoic acid	0.991	54.675	295.227	5.40E-04	3.09E-04	1.171	0.0012	0.0042	1.748	0.806
26	16-Hydroxy hexadecanoic acid	0.984	76.769	271.228	7.33E-05	3.27E-05	1.391	0.0056	0.0132	2.242	1.165
27	9,10-DHOME	0.948	65.547	313.238	1.08E-04	6.04E-05	1.202	0.0003	0.0016	1.790	0.840
28	Traumatic acid	0.862	241.441	227.128	3.63E-04	1.28E-04	1.615	0.0000	0.0003	2.841	1.506
29	(9xi,10xi,12xi)-9,10-Dihydroxy-12-octadecenoic acid	0.702	79.663	313.238	8.32E-05	3.91E-05	1.369	0.0002	0.0013	2.130	1.091
30	2-Isopropylmalic acid	0.600	64.551	175.060	1.05E-04	5.48E-05	1.260	0.0017	0.0054	1.913	0.936
31	(9S,10E,12Z,15Z)-9-Hydroxy-10,12,15-octadecatrienoic acid	0.930	36.134	277.215	3.18E-05	1.68E-05	1.274	0.0021	0.0094	1.890	0.918
32	Linoleoyl ethanolamide	0.856	35.904	324.289	7.17E-05	1.90E-05	1.859	0.0003	0.0025	3.770	1.915

33	Deacetylisovaltrate	0.763	396.479	381.188	1.42E-05	5.20E-08	3.838	0.0181	0.0438	273.042	8.093
34	MG(0:0/18:3(6Z,9Z,12Z)/0:0)	0.741	35.927	353.268	3.01E-05	1.21E-05	1.461	0.0408	0.0751	2.494	1.318
35	(2'E,4'Z,7'Z,8E)-Colnelenic acid	0.703	63.346	293.211	1.41E-05	6.55E-06	1.343	0.0152	0.0389	2.158	1.110
36	MG(0:0/18:4(6Z,9Z,12Z,15Z)/0:0)	0.695	36.239	351.252	4.05E-05	1.56E-05	1.565	0.0029	0.0118	2.604	1.381
37	LysoPC(14:0/0:0)	0.537	228.128	468.308	2.11E-05	8.16E-06	1.563	0.0010	0.0058	2.586	1.371
38	LysoPC(18:3(6Z,9Z,12Z))	0.534	225.366	518.324	7.11E-04	3.89E-04	1.170	0.0375	0.0715	1.828	0.870
39	LysoPC(16:1(9Z)/0:0)	0.528	225.425	494.325	2.11E-05	1.12E-05	1.193	0.0352	0.0687	1.891	0.919
40	Alpha-Linolenic acid	1.000	50.315	277.217	7.37E-04	4.52E-04	1.038	0.0314	0.0311	1.630	0.705
41	Panaquinquecol 1	0.653	203.871	293.211	1.32E-06	3.52E-07	1.877	0.0197	0.0464	3.762	1.912
42	Daidzein	0.999	260.581	253.050	8.78E-03	3.84E-03	1.411	0.0011	0.0040	2.283	1.191
43	6''-Malonylgenistin	0.965	240.327	519.114	1.25E-02	7.27E-03	1.185	0.0004	0.0029	1.716	0.779
44	2'-Hydroxygenistein 7-(6''-malonylglucoside)	0.834	266.351	535.108	7.39E-05	4.03E-05	1.236	0.0024	0.0103	1.833	0.874
45	(-)-Epiafzelechin	0.962	58.128	273.076	3.78E-03	7.22E-04	2.034	0.0000	0.0004	5.234	2.388
46	5,7-dihydroxy-2-(4-hydroxy-3-methoxyphenyl)-3-{[3,4,5-trihydroxy-6-(hydroxymethyl)oxan-2-yl]oxy}-4H-chromen-4-one	0.782	179.797	477.104	8.49E-05	5.01E-05	1.077	0.0200	0.0266	1.694	0.760
47	Norizalpinin	0.754	294.630	269.045	1.00E-04	4.77E-05	1.345	0.0008	0.0031	2.106	1.075
48	3,5,7-trihydroxy-2-(4-hydroxyphenyl)-3,4-dihydro-2H-1-benzopyran-4-one	0.521	58.759	287.056	2.26E-03	5.26E-04	1.907	0.0003	0.0016	4.300	2.104
49	Cosmosiin	0.996	201.839	433.112	3.31E-03	1.17E-04	2.960	0.0125	0.0339	28.315	4.824
50	Luteolin	0.994	521.279	287.054	1.53E-04	3.27E-05	1.992	0.0256	0.0559	4.688	2.229
51	Peonidin-3-glucoside	0.990	153.425	463.123	3.02E-04	5.95E-05	2.066	0.0000	0.0007	5.068	2.341
52	Sideritiflavone	0.989	102.955	361.091	2.97E-05	1.14E-05	1.583	0.0000	0.0006	2.602	1.379
53	Isorhamnetin	0.981	192.786	317.066	1.49E-04	3.72E-05	1.911	0.0000	0.0002	4.003	2.001
54	Luteolin 7-galactoside	0.944	241.710	449.107	4.30E-03	2.82E-04	2.674	0.0080	0.0250	15.215	3.927
55	Trilobatin	0.865	202.976	437.143	1.22E-05	1.69E-06	2.285	0.0013	0.0068	7.198	2.848
56	Citrusinine II	0.448	60.782	288.086	5.56E-04	1.07E-04	2.072	0.0116	0.0324	5.197	2.378
57	2-(3,4-dihydroxyphenyl)-5,7-dihydroxy-6-[3,4,5-trihydroxy-6-(hydroxymethyl)oxan-2-yl]-4H-chromen-4-one	0.675	278.916	449.107	3.50E-04	3.49E-05	2.441	0.0263	0.0569	10.041	3.328
58	Carthamone	0.959	475.950	449.107	8.95E-04	1.74E-04	2.073	0.0013	0.0069	5.133	2.360
59	Marmesin	0.897	136.361	245.082	1.01E-04	4.92E-05	1.282	0.0181	0.0256	2.053	1.038
60	benzene-1,2,4-triol	0.967	392.763	125.023	2.17E-04	1.37E-04	1.034	0.0055	0.0130	1.582	0.662
61	Inosine	0.879	237.145	267.073	2.96E-05	1.31E-05	1.383	0.0079	0.0166	2.248	1.168
62	Cytidine 2'-phosphate	0.997	456.936	324.059	4.29E-05	1.26E-05	1.772	0.0020	0.0093	3.389	1.761
63	Abscisic acid	0.693	107.366	265.143	9.10E-06	1.11E-06	2.320	0.0343	0.0677	8.187	3.033
64	Thymine	0.918	94.277	127.050	1.51E-05	5.20E-08	3.859	0.0123	0.0336	289.567	8.178
65	5-Aminoimidazole ribonucleotide	0.498	407.904	296.066	2.28E-04	1.14E-04	1.329	0.0173	0.0423	1.993	0.995

66	Malvidin 3-glucoside	0.839	100.318	493.133	1.34E-04	5.22E-05	1.572	0.0000	0.0006	2.565	1.359
67	Dopamine	0.447	310.717	299.077	4.91E-05	2.47E-05	1.266	0.0163	0.0244	1.991	0.993
68	2,4,6-trihydroxy-2-[(4-hydroxyphenyl)methyl]-2,3-dihydro-1-benzofuran-3-one	0.988	293.589	271.060	1.33E-04	5.35E-05	1.546	0.0000	0.0005	2.480	1.311
69	Aflatoxin G2	0.879	121.020	331.080	1.50E-04	3.62E-05	1.934	0.0000	0.0001	4.131	2.046
70	Capsiate	0.838	58.911	307.189	3.10E-06	5.34E-07	2.170	0.0009	0.0052	5.801	2.536
71	Diferuloylputrescine	0.808	56.704	441.201	4.94E-05	3.12E-06	2.702	0.0001	0.0011	15.807	3.982
72	Dihydrotestosterone	0.756	31.617	291.231	8.32E-06	2.60E-06	1.725	0.0035	0.0135	3.199	1.678
73	Bisacurone epoxide	0.697	57.045	269.174	3.39E-05	8.93E-06	1.866	0.0001	0.0014	3.792	1.923
74	Diplodiatoxin	0.645	104.406	309.205	1.26E-05	7.71E-07	2.713	0.0073	0.0233	16.292	4.026
75	3,11,12-Trihydroxy-1(10)-spirovetiven-2-one	0.561	37.691	269.174	1.97E-05	4.21E-06	2.007	0.0005	0.0035	4.671	2.224
76	3-(4-Isopropylphenyl)propanal	0.780	62.420	177.127	9.20E-06	4.55E-06	1.325	0.0032	0.0127	2.022	1.016
77	Vanillin 3-(L-menthoxy)propane-1,2-diol acetal	0.626	50.251	365.231	5.62E-06	2.62E-06	1.301	0.0428	0.0772	2.144	1.100
78	Imidazoleacetic acid	0.894	95.257	125.034	1.81E-04	1.16E-04	1.004	0.0187	0.0259	1.557	0.639
79	Protoporphyrin IX	0.776	309.273	561.254	1.58E-04	8.65E-05	1.225	0.0000	0.0004	1.826	0.868
80	Nicotine	0.906	69.482	163.123	1.66E-04	9.80E-05	1.167	0.0003	0.0027	1.694	0.760
81	Syringic acid	0.674	85.059	197.045	3.38E-05	1.96E-05	1.087	0.0187	0.0259	1.727	0.788
82	Phthalic acid	0.643	132.115	165.018	2.97E-04	9.93E-05	1.650	0.0001	0.0008	2.995	1.582
83	Phenylglyoxylic acid	0.550	56.198	149.023	3.95E-05	2.44E-05	1.045	0.0164	0.0244	1.620	0.696
84	[8]-Paradyl acetate	0.637	36.838	349.237	2.29E-05	1.20E-05	1.253	0.0180	0.0435	1.914	0.937
85	Norophthalmic acid	0.581	437.290	276.119	2.19E-05	1.33E-05	1.043	0.0463	0.0806	1.638	0.712
86	2-Aminoheptanedioic acid	0.565	285.538	176.091	3.04E-04	5.91E-05	2.072	0.0048	0.0171	5.135	2.360
87	5-Heptyltetrahydro-2-oxo-3-furancarboxylic acid	0.525	58.136	229.143	1.41E-05	5.28E-06	1.581	0.0033	0.0129	2.671	1.417
88	Gerberinol	0.582	190.484	365.102	3.11E-05	1.38E-05	1.414	0.0093	0.0277	2.256	1.173
89	Pisumsaponin II	0.470	300.376	941.511	1.78E-04	7.65E-05	1.491	0.0000	0.0002	2.331	1.221
90	Calendulaglycoside E	0.453	262.060	795.450	8.69E-05	4.21E-05	1.375	0.0001	0.0013	2.063	1.045
91	Menthone 1,2-glyceryl ketal	0.913	437.548	298.100	1.52E-06	5.74E-07	1.529	0.0431	0.0775	2.656	1.409
92	3alpha,4,7,7alpha-Tetrahydro-4-hydroxy-1H-isoindole-1,3(2H)-dione	0.774	86.994	168.065	2.46E-04	1.38E-04	1.229	0.0002	0.0020	1.784	0.835
93	Pyridoxine	0.961	102.470	170.081	2.39E-03	1.60E-03	1.023	0.0002	0.0016	1.493	0.578
94	Dukunolide B	0.977	353.105	499.157	4.82E-05	2.67E-05	1.229	0.0195	0.0460	1.807	0.854
95	Thiamine monophosphate	0.952	514.422	345.078	3.65E-05	2.19E-05	1.147	0.0007	0.0043	1.664	0.734
96	Linalyl phenylacetate	0.828	36.449	273.184	2.70E-06	8.93E-07	1.599	0.0424	0.0767	3.028	1.598
97	N-Acetylleucine	0.887	219.207	172.097	5.28E-05	1.73E-04	1.843	0.0387	0.0329	0.305	-1.714
98	L-Tryptophan	0.526	74.867	203.082	1.08E-05	4.83E-04	3.086	0.0003	0.0016	0.022	-5.482
99	(alpha-D-mannosyl)7-beta-D-mannosyl-diacetylchitobiosyl-L-asparagine, isoform A (protein)	1.000	368.834	90.055	5.52E-04	8.60E-04	1.077	0.0000	0.0007	0.642	-0.639
100	Prolyl-Asparagine	0.907	350.807	230.113	4.28E-07	3.13E-06	2.309	0.0026	0.0111	0.137	-2.868

101	Phenylalanyl-Methionine	0.871	208.384	297.126	5.38E-08	6.19E-06	3.534	0.0053	0.0185	0.009	-6.847
102	Histidinal	0.836	152.336	140.082	1.22E-04	1.86E-04	1.057	0.0001	0.0012	0.653	-0.616
103	Tryptophyl-Tyrosine	0.817	430.260	368.156	5.38E-08	2.78E-06	3.223	0.0048	0.0172	0.019	-5.690
104	Arginyl-Phenylalanine	0.791	362.105	322.187	8.22E-06	1.58E-05	1.251	0.0168	0.0416	0.521	-0.940
105	Histidinyl-Phenylalanine	0.773	300.749	303.144	5.60E-07	4.88E-06	2.734	0.0013	0.0068	0.115	-3.123
106	Lysyl-Valine	0.766	552.461	246.181	2.00E-05	3.34E-05	1.097	0.0274	0.0586	0.597	-0.743
107	N6-Acetyl-L-lysine	0.750	371.344	189.123	6.14E-05	1.20E-04	1.331	0.0000	0.0003	0.509	-0.973
108	Tyrosyl-Glutamine	0.709	86.130	310.139	8.84E-06	5.05E-05	2.141	0.0000	0.0004	0.175	-2.513
109	Valyl-Asparagine	0.669	331.395	232.129	2.53E-06	6.59E-06	1.566	0.0213	0.0492	0.384	-1.381
110	Lysyl-Methionine	0.580	403.764	278.153	5.02E-07	2.41E-06	2.058	0.0148	0.0382	0.209	-2.262
111	Prolyl-Gamma-glutamate	0.579	448.928	244.140	5.23E-07	1.37E-05	2.949	0.0000	0.0004	0.038	-4.717
112	Tryptophyl-Methionine	0.578	219.271	336.137	5.38E-08	4.21E-07	2.319	0.0122	0.0335	0.128	-2.969
113	Gamma-Aminobutyryl-lysine	0.402	99.530	232.165	1.41E-07	1.46E-06	2.510	0.0149	0.0385	0.097	-3.372
114	N-Acetyl-glucosamine 1-phosphate	0.594	464.383	300.049	1.73E-05	3.15E-05	1.118	0.0488	0.0348	0.549	-0.866
115	Sucrose	0.999	408.433	365.106	9.10E-05	2.48E-04	1.616	0.0002	0.0022	0.366	-1.448
116	D-Arabinose 5-phosphate	0.608	436.115	213.016	1.31E-07	1.46E-06	2.574	0.0058	0.0196	0.089	-3.483
117	1-Deoxy-D-glucitol	0.574	127.835	167.092	3.62E-04	4.33E-03	2.559	0.0000	0.0000	0.083	-3.583
118	9-Hydroxy-7-megastigmen-3-one glucoside	0.566	440.555	373.219	1.70E-06	3.15E-06	1.227	0.0104	0.0298	0.539	-0.893
119	Goyaglycoside e	0.483	313.075	781.474	6.09E-06	1.27E-05	1.362	0.0018	0.0085	0.481	-1.056
120	Melibiose	0.467	416.475	360.150	1.21E-04	3.75E-04	1.748	0.0351	0.0686	0.322	-1.634
121	Lithocholate 3-O-glucuronide	0.443	327.219	553.334	5.38E-08	2.77E-06	3.191	0.0454	0.0797	0.019	-5.686
122	Marmesin rhamnoside	0.411	93.043	393.151	4.77E-05	8.21E-05	1.156	0.0051	0.0179	0.581	-0.784
123	Mesaconic acid	0.994	467.967	129.018	5.08E-04	8.04E-04	1.068	0.0001	0.0006	0.632	-0.663
124	(R)-3-Hydroxy-tetradecanoic acid	0.684	71.298	243.196	1.07E-05	2.91E-05	1.547	0.0029	0.0082	0.368	-1.443
125	2-Oxo-4-methylthiobutanoic acid	0.847	389.364	149.027	5.38E-08	5.88E-06	3.510	0.0146	0.0378	0.009	-6.774
126	gamma-Aminobutyric acid	0.980	170.569	104.071	5.56E-05	3.62E-04	2.211	0.0004	0.0033	0.154	-2.701
127	Dihydrozeatin-O-glucoside	0.800	411.501	384.187	1.11E-06	4.34E-06	1.934	0.0073	0.0233	0.256	-1.966
128	Isopentyl beta-D-glucoside	0.727	159.254	251.150	1.29E-06	2.20E-05	2.814	0.0036	0.0137	0.058	-4.098
129	Genistein	0.999	37.315	269.045	1.18E-03	3.27E-03	1.551	0.0087	0.0176	0.360	-1.475
130	Glycitin	0.847	201.651	445.113	3.54E-05	6.52E-05	1.231	0.0002	0.0014	0.543	-0.881
131	6-Hydroxydaidzein 4'-glucoside	0.989	155.042	433.112	4.58E-04	1.56E-03	1.735	0.0118	0.0326	0.293	-1.771
132	Genistein 5-glucoside	0.988	126.809	433.112	9.47E-03	1.78E-02	1.290	0.0000	0.0004	0.531	-0.914
133	6''-O-Acetylgenistin	0.969	161.269	475.123	5.61E-05	3.32E-04	2.164	0.0000	0.0001	0.169	-2.566
134	6''-O-Acetyldaidzin	0.964	61.653	459.129	2.22E-04	2.11E-03	2.436	0.0000	0.0002	0.105	-3.250
135	6''-O-Acetylglycitin	0.946	62.523	489.139	7.32E-05	6.30E-04	2.381	0.0000	0.0001	0.116	-3.105
136	Genistin	0.937	219.125	433.113	4.94E-04	8.58E-04	1.197	0.0005	0.0037	0.575	-0.797
137	6''-O-Malonyldaidzin	0.723	214.586	503.119	3.78E-04	6.85E-04	1.250	0.0000	0.0002	0.552	-0.856
138	Ononin	0.684	118.233	431.134	2.69E-05	7.33E-05	1.621	0.0001	0.0010	0.367	-1.447
139	Luteolin 7-glucoside	0.964	189.276	447.093	1.07E-03	1.73E-03	1.087	0.0006	0.0024	0.618	-0.694
140	Daidzin	0.937	163.212	415.102	1.97E-03	4.53E-03	1.445	0.0000	0.0001	0.434	-1.203
141	Diosmetin	0.924	114.005	299.055	1.88E-05	7.12E-05	1.820	0.0000	0.0004	0.265	-1.918
142	2-(3,4-dihydroxyphenyl)-3,5-dihydroxy-7-methoxy-4H-chromen-4-one	0.880	51.974	315.051	1.69E-04	6.95E-04	1.870	0.0004	0.0019	0.243	-2.038

143	Liquiritin	0.713	164.280	417.119	6.57E-05	1.56E-04	1.457	0.0017	0.0056	0.421	-1.248
144	Naringin	0.653	224.806	579.172	7.42E-08	2.22E-04	4.478	0.0015	0.0049	0.000	-11.545
145	5,7-dihydroxy-2-phenyl-6-[3,4,5-trihydroxy-6-(hydroxymethyl)oxan-2-yl]-8-(3,4,5-trihydroxyoxan-2-yl)-4H-chromen-4-one	0.408	81.574	547.145	4.37E-05	3.55E-04	2.290	0.0000	0.0000	0.123	-3.021
146	Naringenin	0.984	175.125	273.075	1.53E-04	3.34E-04	1.303	0.0493	0.0833	0.459	-1.122
147	Cyanidin 3-(4-acetylglucoside)	0.960	117.334	491.118	1.15E-05	1.44E-04	2.580	0.0000	0.0000	0.080	-3.641
148	Apigenin 7-O-(6''-O-acetylglucoside)	0.943	107.416	475.124	2.06E-06	5.09E-05	2.914	0.0000	0.0001	0.040	-4.628
149	6''-O-Malonylwistin	0.829	91.712	547.145	5.38E-08	9.21E-06	3.682	0.0015	0.0077	0.006	-7.421
150	4',5,6-Trimethylscutellarein 7-glucoside	0.800	181.813	491.154	6.15E-05	9.36E-05	1.033	0.0023	0.0099	0.657	-0.607
151	4'-Methylliquiritigenin 7-rhamnoside	0.410	143.305	417.154	5.38E-08	1.03E-05	3.720	0.0073	0.0232	0.005	-7.583
152	Feruloyl-2-hydroxyputrescine	1.000	152.309	281.149	4.88E-06	8.05E-05	2.809	0.0000	0.0005	0.061	-4.043
153	3-(2-Hydroxyphenyl)propanoic acid	0.821	117.611	165.055	1.02E-04	3.12E-04	2.162	0.0312	0.0310	0.325	-1.620
154	3,3',4'5-Tetrahydroxystilbene	0.933	116.753	243.066	1.42E-05	7.49E-05	2.029	0.0002	0.0011	0.190	-2.396
155	Pyrocatechol	0.993	44.801	109.028	3.69E-05	2.15E-04	2.093	0.0002	0.0011	0.172	-2.540
156	Maltol	1.000	69.911	127.039	9.45E-05	1.26E-03	2.612	0.0000	0.0000	0.075	-3.731
157	Phlorin	0.994	164.109	289.091	4.75E-04	5.99E-03	2.585	0.0002	0.0021	0.079	-3.658
158	Quinic acid	0.986	356.415	191.055	2.82E-05	4.94E-05	1.156	0.0025	0.0074	0.571	-0.809
159	Glucose 1-phosphate	0.559	476.469	259.022	6.95E-07	1.01E-04	3.648	0.0099	0.0189	0.007	-7.183
160	3-(4-Hydroxyphenyl)-1-propanol	0.509	34.180	153.091	6.49E-06	1.53E-05	1.500	0.0000	0.0006	0.424	-1.237
161	4-Aminophenol	0.944	51.147	110.060	7.71E-05	2.77E-04	1.823	0.0162	0.0407	0.278	-1.848
162	Cytidine	0.985	261.061	242.078	1.55E-05	2.93E-05	1.256	0.0002	0.0014	0.527	-0.923
163	Deoxyinosine	0.868	197.586	251.078	2.54E-05	4.56E-05	1.192	0.0022	0.0066	0.556	-0.846
164	3'-AMP	0.861	428.259	346.056	2.25E-07	8.22E-06	3.094	0.0146	0.0232	0.027	-5.191
165	Cytarabine	1.000	260.517	244.093	6.75E-05	1.08E-04	1.106	0.0043	0.0157	0.626	-0.677
166	Guanosine	0.995	285.853	284.099	1.44E-04	2.33E-04	1.121	0.0001	0.0011	0.619	-0.692
167	1-Methyladenosine	0.987	132.484	282.120	1.15E-05	1.15E-04	2.465	0.0004	0.0030	0.100	-3.324
168	5'-Methylthioadenosine	0.975	93.396	298.096	3.81E-04	2.29E-03	2.175	0.0000	0.0000	0.166	-2.589
169	Adenosine 2'-phosphate	0.964	428.737	348.070	2.84E-06	1.64E-05	2.149	0.0001	0.0012	0.173	-2.533
170	Guanosine monophosphate	0.958	479.913	364.065	2.79E-06	9.90E-06	1.813	0.0133	0.0354	0.282	-1.828
171	1-Methylguanine	0.481	205.999	166.072	2.91E-04	7.98E-04	1.619	0.0005	0.0037	0.365	-1.455
172	Thymidine	0.989	93.249	241.082	1.70E-05	4.42E-05	1.539	0.0003	0.0015	0.385	-1.378
173	Nicotinic acid	0.711	239.168	122.024	8.76E-05	1.43E-04	1.101	0.0000	0.0004	0.614	-0.703
174	Guanine	0.998	285.853	152.057	2.36E-04	4.09E-04	1.200	0.0002	0.0016	0.576	-0.796
175	FAPy-adenine	0.951	188.880	154.072	1.82E-05	1.71E-04	2.434	0.0000	0.0000	0.106	-3.238
176	Dihydrouracil	0.831	60.773	115.050	9.30E-06	1.28E-04	2.629	0.0000	0.0001	0.073	-3.781
177	Citicoline	0.849	462.516	489.115	2.86E-05	5.85E-05	1.324	0.0240	0.0534	0.489	-1.033
178	alpha-Mangostin	0.459	295.024	409.165	1.26E-06	4.24E-06	1.690	0.0245	0.0287	0.297	-1.752
179	2-Hydroxypyridine	0.999	52.794	96.045	4.50E-05	3.41E-04	2.309	0.0000	0.0000	0.132	-2.919
180	Niacinamide	0.995	60.806	123.055	3.23E-04	8.95E-03	2.960	0.0000	0.0000	0.036	-4.793

181	5-(2-Hydroxyethyl)-4-methylthiazole	0.985	48.170	144.048	2.16E-05	8.53E-05	1.874	0.0028	0.0115	0.253	-1.981
182	7-Hydroxymethyl-12-methylbenz[a]anthracene sulfate	0.977	70.362	353.084	1.35E-05	1.57E-04	2.522	0.0241	0.0536	0.086	-3.533
183	Beta-Carboline	0.918	50.130	169.076	1.46E-05	2.26E-05	1.060	0.0003	0.0023	0.648	-0.625
184	9-(beta-D-Ribofuranosyl)zeatin	0.762	136.573	352.160	3.87E-07	1.23E-05	3.111	0.0002	0.0016	0.031	-4.992
185	Oroselone	0.752	160.403	227.070	7.68E-06	1.92E-05	1.488	0.0164	0.0409	0.401	-1.319
186	5-(2-Furanyl)-3,4-dihydro-2H-pyrrole	0.746	103.907	136.076	5.44E-05	9.15E-05	1.154	0.0014	0.0071	0.594	-0.751
187	Theaspirone A	0.710	197.130	209.153	5.86E-05	9.03E-05	1.041	0.0029	0.0119	0.649	-0.625
188	Benzofuran	0.536	325.574	119.049	1.20E-05	3.10E-05	1.527	0.0119	0.0328	0.388	-1.366
189	2-Benzofurancarboxaldehyde	0.813	325.745	147.044	2.07E-05	3.14E-05	1.036	0.0005	0.0035	0.660	-0.599
190	Pyridoxal	0.649	69.656	168.065	2.70E-05	4.88E-05	1.226	0.0222	0.0506	0.554	-0.852
191	Xanthine	0.965	233.541	151.025	3.03E-05	1.18E-04	1.840	0.0000	0.0003	0.256	-1.966
192	Betaine	0.999	293.780	118.086	1.34E-03	2.11E-03	1.081	0.0002	0.0022	0.638	-0.648
193	3-Isoxazolidinone	0.999	397.408	88.040	2.49E-05	4.75E-05	1.287	0.0007	0.0047	0.525	-0.929
194	Pyrrolidonecarboxylic acid	1.000	320.278	128.034	9.97E-04	2.71E-03	1.583	0.0000	0.0001	0.368	-1.441
195	Oxoglutaric acid	0.989	388.681	145.013	1.93E-04	2.97E-04	1.019	0.0021	0.0064	0.649	-0.623
196	Phenylpyruvic acid	0.937	186.597	163.039	4.79E-04	8.45E-04	1.190	0.0001	0.0005	0.567	-0.818
197	3-(4-hydroxy-3-methoxyphenyl)prop-2-enoic acid	0.851	167.700	193.050	5.24E-05	1.83E-04	1.771	0.0020	0.0063	0.286	-1.807
198	2-Pyrrolidineacetic acid	0.995	65.210	130.086	4.90E-05	1.60E-04	1.756	0.0009	0.0053	0.306	-1.710
199	Sebiferic acid	0.617	303.963	441.373	8.19E-05	1.41E-04	1.152	0.0052	0.0182	0.580	-0.787
200	Cinncassiol C3	0.728	420.665	383.203	9.71E-06	2.54E-05	1.564	0.0017	0.0081	0.382	-1.387
201	Jujubasaponin VI	0.393	270.451	797.468	1.91E-04	4.45E-04	1.466	0.0007	0.0046	0.429	-1.220
202	Jujubasaponin IV	0.352	306.007	943.524	4.69E-04	7.95E-04	1.127	0.0332	0.0663	0.590	-0.761
203	Sandosaponin B	0.343	317.124	957.506	1.31E-05	4.97E-05	1.855	0.0008	0.0049	0.264	-1.921
204	5-Phenyl-1,3-oxazinane-2,4-dione	0.936	50.640	192.065	7.28E-06	1.42E-05	1.282	0.0061	0.0203	0.513	-0.962
205	2-Pyrrolidinone	0.912	394.397	86.061	8.22E-04	1.52E-03	1.268	0.0000	0.0003	0.542	-0.883
206	3-[(3-Methylbutyl)nitrosoamino]-2-butanone	0.576	495.802	187.144	4.80E-05	1.46E-04	1.710	0.0000	0.0003	0.328	-1.609
207	(3R,8E)-3-Hydroxy-5,8-megastigmadien-7-one	0.560	229.045	209.153	1.31E-04	2.02E-04	1.070	0.0000	0.0001	0.647	-0.627
208	O-Phosphoethanolamine	0.998	489.014	140.011	1.11E-05	2.02E-05	1.123	0.0361	0.0324	0.550	-0.864
209	Methyl acrylate	0.991	395.225	87.045	7.98E-04	1.40E-03	1.218	0.0000	0.0006	0.568	-0.815
210	Ethylparaben	0.585	45.843	165.055	4.18E-05	2.09E-04	2.007	0.0000	0.0003	0.200	-2.323

Note: rt is the chromatographic retention time of the substance; Mass is the exact molecular weight of the metabolite; score is the match with the substance in the database; The MEAN r is the relative quantitative mean of the substances in the unprocessed black bean group; The MEAN ops is the relative quantitative mean of the substances in the steamed black bean group; VIP is the variable projection importance obtained by the OPLS-DA model of the comparison in this group; P-VALUE is the P value obtained by the t-test of the comparison in this group, P value = the probability that the hypothesis is correct but rejected = the number of negative results / the total number of results, which is a test probability for the sample data; Q-VALUE is the hypothesis test statistic (P value) after correction for multiple hypothesis testing The result of , Q value = probability of being

rejected but correct = number of false positive results / number of presumed positive results, which is a test probability for the inference obtained by the t test, and a re-statistic for the P value; FOLD CHANGE is the fold relationship of the substance in the comparison between the two groups of experiments; LOG_FOLDCHANGE FOLD CHANGE is the logarithm with the base 2, "-" means the relative content is low.

Table S4 List of differential metabolites between cooked black soybeans and Steamed black soybeans

	Name	score	rt	mz	MEAN ops	MEAN p	VIP	P-VALUE	Q-VALUE	FOLD CHANGE	LOG_FOLDCHANGE
1	L-Proline	0.999	333.297	114.055	3.97E-04	1.72E-04	1.464	0.0014	0.0080	2.302	1.203
2	L-Glutamic acid	0.993	417.911	146.045	2.85E-03	1.61E-03	1.195	0.0008	0.0061	1.770	0.824
3	L-Asparagine	0.993	398.701	131.045	1.04E-04	2.91E-06	3.496	0.0026	0.0111	35.576	5.153
4	L-Tyrosine	0.962	326.112	182.081	8.35E-04	3.84E-04	1.315	0.0001	0.0001	2.172	1.119
5	D-Alanyl-D-alanine	0.960	338.297	159.076	7.47E-05	3.85E-05	1.223	0.0164	0.0262	1.941	0.957
6	N-Acetylleucine	0.887	219.207	172.097	1.73E-04	7.39E-05	1.447	0.0412	0.0315	2.344	1.229
7	Epsilon-(gamma-Glutamyl)-lysine	0.744	510.723	276.155	2.31E-06	6.22E-07	1.796	0.0190	0.0094	3.720	1.895
8	L-Phenylalanine	0.998	281.562	166.086	5.59E-03	3.49E-03	1.020	0.0003	0.0002	1.599	0.677
9	Glycitein	0.939	260.086	283.061	1.26E-03	5.68E-04	1.425	0.0149	0.0255	2.224	1.153
10	D-Glutamine	0.947	396.057	145.061	3.07E-05	6.24E-06	2.016	0.0001	0.0026	4.918	2.298
11	Isoleucyl-Serine	0.984	281.566	219.133	1.30E-04	5.20E-05	1.396	0.0287	0.0137	2.498	1.321
12	3-Methylhistidine	0.962	404.297	170.092	9.25E-04	5.51E-04	1.073	0.0005	0.0003	1.679	0.747
13	Leucyl-Tyrosine	0.932	231.431	295.165	2.79E-05	8.07E-06	1.659	0.0039	0.0022	3.458	1.790
14	(alpha-D-mannosyl)7-beta-D-mannosyl-diacetylchitobiosyl-L-asparagine, isoform A (protein)	1.000	368.834	90.055	8.60E-04	3.48E-04	1.425	0.0000	0.0001	2.472	1.305
15	4,5-Dihydroorotic acid	0.736	176.453	157.026	5.49E-05	3.37E-05	1.065	0.0124	0.0240	1.630	0.705
16	gamma-Glutamylglutamic acid	0.586	474.535	277.103	7.48E-05	3.87E-05	1.179	0.0052	0.0029	1.932	0.950
17	Histidinal	0.836	152.336	140.082	1.86E-04	9.10E-05	1.270	0.0000	0.0001	2.049	1.035
18	Histidinyl-Phenylalanine	0.773	300.749	303.144	4.88E-06	5.56E-07	2.265	0.0002	0.0001	8.784	3.135
19	Isoleucyl-Tryptophan	0.643	210.846	318.181	4.57E-05	2.21E-05	1.245	0.0020	0.0011	2.066	1.047
20	L-Tryptophan	0.526	74.867	203.082	4.83E-04	2.49E-04	1.281	0.0013	0.0078	1.940	0.956
21	N6-Acetyl-L-lysine	0.750	371.344	189.123	1.20E-04	5.70E-05	1.290	0.0001	0.0001	2.115	1.080
22	Phenylalanyl-Gamma-glutamate	0.710	283.277	294.145	3.98E-06	8.77E-07	1.812	0.0089	0.0047	4.535	2.181
23	Prolyl-Gamma-glutamate	0.579	448.928	244.140	1.37E-05	6.35E-06	1.310	0.0002	0.0002	2.164	1.113
24	Tryptophyl-Methionine	0.578	219.271	336.137	4.21E-07	6.61E-08	2.034	0.0131	0.0067	6.369	2.671
25	Tryptophyl-Tyrosine	0.817	430.260	368.156	2.78E-06	6.61E-08	2.897	0.0049	0.0027	42.016	5.393
26	Tyrosyl-Alanine	0.729	268.206	253.118	1.24E-05	6.10E-06	1.254	0.0003	0.0002	2.037	1.026
27	Tyrosyl-Aspartate	0.636	392.509	297.108	2.83E-06	1.32E-06	1.216	0.0363	0.0169	2.146	1.101
28	Tyrosyl-Gamma-glutamate	0.590	320.991	310.139	2.18E-06	5.73E-07	1.684	0.0078	0.0042	3.800	1.926
29	Tyrosyl-Glutamine	0.709	86.130	310.139	5.05E-05	2.86E-05	1.113	0.0013	0.0007	1.766	0.820
30	Tyrosyl-Phenylalanine	0.707	215.498	329.149	2.40E-05	1.93E-06	2.489	0.0324	0.0152	12.432	3.636
31	Tyrosyl-Threonine	0.820	281.561	283.128	1.05E-05	3.25E-06	1.607	0.0011	0.0007	3.226	1.690
32	Tyrosyl-Tryptophan	0.768	229.660	368.160	2.83E-06	3.97E-07	2.113	0.0063	0.0034	7.136	2.835
33	Valyl-Asparagine	0.669	331.395	232.129	6.59E-06	2.04E-06	1.604	0.0021	0.0012	3.234	1.693
34	Valyl-Tryptophan	0.571	217.277	304.165	6.21E-06	8.89E-07	2.193	0.0074	0.0040	6.983	2.804
35	4-Methoxybenzyl glucoside	0.469	141.861	301.129	4.10E-05	1.62E-05	1.427	0.0023	0.0013	2.535	1.342
36	4'-Methylliquiritigenin	0.410	143.305	417.154	1.03E-05	6.61E-08	3.368	0.0073	0.0039	156.06	7.286

	7-rhamnoside									6	
37	Aldehydo-D-xylose	0.764	336.445	149.044	3.83E-04	2.31E-04	1.116	0.0021	0.0099	1.661	0.732
38	Gluconic acid	0.913	396.520	195.050	1.72E-03	9.73E-04	1.183	0.0020	0.0098	1.764	0.819
39	Hydroxytyrosol 1-O-glucoside	0.841	204.073	317.126	1.81E-06	3.39E-07	1.931	0.0032	0.0018	5.352	2.420
40	L-Arabitol	0.993	97.798	151.060	1.57E-04	9.47E-05	1.132	0.0003	0.0036	1.660	0.732
41	Marmesin rhamnoside	0.411	93.043	393.151	8.21E-05	3.97E-05	1.260	0.0008	0.0005	2.066	1.047
42	Melibiose	0.467	416.475	360.150	3.75E-04	1.09E-04	1.671	0.0207	0.0102	3.428	1.777
43	N-Acetyl-glucosamine 1-phosphate	0.594	464.383	300.049	3.15E-05	1.45E-05	1.289	0.0416	0.0316	2.176	1.122
44	Rhamnose	0.782	151.216	163.060	1.30E-04	6.46E-05	1.326	0.0005	0.0049	2.006	1.005
45	Threonic acid	0.915	334.521	135.029	3.55E-04	1.11E-04	1.732	0.0000	0.0007	3.213	1.684
46	2-Methylglutaric acid	0.846	72.160	145.050	2.09E-04	1.29E-04	1.050	0.0309	0.0302	1.625	0.701
47	2-Oxo-4-methylthiobutanoic acid	0.847	389.364	149.027	5.88E-06	1.08E-06	2.364	0.0180	0.0090	5.432	2.442
48	Itaconic acid	0.973	66.828	129.018	9.80E-04	6.06E-04	1.080	0.0259	0.0292	1.619	0.695
49	Mesaconic acid	0.994	467.967	129.018	8.04E-04	4.75E-04	1.146	0.0110	0.0230	1.692	0.759
50	Methylsuccinic acid	0.984	374.444	131.034	4.78E-05	1.29E-05	2.092	0.0148	0.0254	3.718	1.894
51	Dihydrozeatin-O-glucoside	0.800	411.501	384.187	4.34E-06	8.76E-07	1.889	0.0010	0.0006	4.954	2.309
52	Isopentyl beta-D-glucoside	0.727	159.254	251.150	2.20E-05	1.14E-05	1.127	0.0347	0.0162	1.927	0.947
53	Jasmonic acid	0.988	100.604	209.117	5.49E-06	1.85E-06	1.603	0.0104	0.0225	2.963	1.567
54	Glycerol 3-phosphate	0.849	453.589	171.005	9.24E-05	9.58E-08	4.211	0.0007	0.0058	964.151	9.913
55	5,7-Dihydroxy-2',6-dimethoxyisoflavone 7-rhamnoside	0.541	190.466	461.144	3.34E-04	2.06E-04	1.032	0.0005	0.0003	1.616	0.693
56	6'-Malonyltrifolirhizin	0.844	260.005	533.128	9.54E-04	4.08E-04	1.363	0.0004	0.0002	2.336	1.224
57	6''-O-Acetylglycitin	0.946	62.523	489.139	6.30E-04	3.10E-04	1.260	0.0001	0.0001	2.031	1.022
58	Genistein	0.999	37.315	269.045	3.27E-03	9.46E-04	1.764	0.0039	0.0138	3.456	1.789
59	Glycitin	0.847	201.651	445.113	6.52E-05	2.88E-05	1.427	0.0008	0.0059	2.265	1.179
60	2-(3,4-dihydroxyphenyl)-3,5-dihydroxy-7-methoxy-4H-chromen-4-one	0.880	51.974	315.051	6.95E-04	3.87E-05	2.729	0.0039	0.0137	17.934	4.165
61	2-(3,4-dihydroxyphenyl)-5,7-dihydroxy-6-[3,4,5-trihydroxy-6-(hydroxymethyl)oxan-2-yl]-4H-chromen-4-one	0.675	278.916	449.107	3.49E-05	4.83E-06	2.103	0.0010	0.0006	7.220	2.852
62	4',5,6-Trimethylscutellarein 7-glucoside	0.800	181.813	491.154	9.36E-05	3.78E-05	1.423	0.0001	0.0001	2.475	1.307
63	5,7-dihydroxy-2-(4-hydroxy-3-methoxyphenyl)-3-{[3,4,5-trihydroxy-6-(hydroxymethyl)oxan-2-yl]oxy}-4H-chromen-4-one	0.782	179.797	477.104	5.01E-05	2.03E-05	1.500	0.0038	0.0136	2.468	1.303
64	5,7-dihydroxy-2-phenyl-6-[3,4,5-trihydroxy-6-(hydroxymethyl)oxan-2-yl]-8-(3,4,5-trihydroxyoxan-2-yl)-4H-chromen-4-one	0.408	81.574	547.145	3.55E-04	2.21E-04	1.069	0.0033	0.0124	1.602	0.680

65	Citrusinine II	0.448	60.782	288.086	1.07E-04	5.52E-05	1.155	0.0110	0.0057	1.938	0.954
66	Cosmosiin	0.996	201.839	433.112	1.17E-04	6.75E-05	1.038	0.0256	0.0123	1.734	0.794
67	Epicatechin	0.938	176.579	291.086	1.99E-06	6.61E-08	2.741	0.0469	0.0212	30.109	4.912
68	Isorhamnetin	0.981	192.786	317.066	3.72E-05	1.85E-05	1.219	0.0024	0.0014	2.010	1.007
69	Luteolin 7-galactoside	0.944	241.710	449.107	2.82E-04	5.76E-05	1.867	0.0025	0.0015	4.905	2.294
70	Naringin	0.653	224.806	579.172	2.22E-04	9.58E-08	4.471	0.0015	0.0082	2314.012	11.176
71	Peonidin-3-glucoside	0.990	153.425	463.123	5.95E-05	2.66E-05	1.262	0.0252	0.0122	2.234	1.159
72	Phlorizin	0.351	225.433	435.130	1.02E-04	5.31E-05	1.292	0.0001	0.0021	1.914	0.937
73	Sideritiflavone	0.989	102.955	361.091	1.14E-05	4.48E-06	1.376	0.0113	0.0059	2.549	1.350
74	(E)-3-(4-Hydroxyphenyl)-2-propenal	0.969	281.452	147.044	7.06E-05	4.15E-05	1.139	0.0051	0.0161	1.702	0.767
75	Subaphylline	0.905	210.486	265.154	1.80E-05	5.63E-06	1.603	0.0282	0.0134	3.187	1.672
76	Protocatechuic acid	0.812	39.594	153.018	6.64E-04	2.94E-04	1.409	0.0075	0.0196	2.259	1.176
77	Acetaminophen	0.937	102.583	152.070	2.81E-04	1.45E-04	1.201	0.0010	0.0006	1.944	0.959
78	Gallic acid	0.649	32.854	169.013	2.17E-04	9.42E-05	1.456	0.0007	0.0057	2.299	1.201
79	Phenol	0.866	325.557	95.050	1.46E-05	1.39E-06	2.751	0.0016	0.0009	10.491	3.391
80	Pyrocatechol	0.993	44.801	109.028	2.15E-04	4.97E-05	1.942	0.0000	0.0016	4.317	2.110
81	Phlorin	0.994	164.109	289.091	5.99E-03	3.23E-03	1.180	0.0000	0.0001	1.856	0.892
82	4-Aminophenol	0.944	51.147	110.060	2.77E-04	1.23E-04	1.329	0.0052	0.0029	2.253	1.172
83	Indoxyl	0.975	144.553	134.060	2.22E-05	1.20E-05	1.150	0.0035	0.0020	1.854	0.891
84	3,4-Dihydroxycinnamoyl-(Z)-2-(3,4-dihydroxyphenyl)ethenol	0.590	152.353	315.085	4.20E-05	2.23E-05	1.185	0.0003	0.0002	1.881	0.911
85	Glucose 1-phosphate	0.559	476.469	259.022	1.01E-04	1.36E-06	3.417	0.0097	0.0219	74.314	6.216
86	myo-Inositol	0.900	412.688	179.055	6.03E-04	3.45E-04	1.190	0.0003	0.0035	1.750	0.808
87	Quinic acid	0.986	356.415	191.055	4.94E-05	2.83E-05	1.181	0.0009	0.0063	1.743	0.802
88	1-Methyladenosine	0.987	132.484	282.120	1.15E-04	2.77E-05	1.771	0.0003	0.0002	4.160	2.057
89	1-Methylguanine	0.481	205.999	166.072	7.98E-04	4.35E-04	1.123	0.0110	0.0057	1.834	0.875
90	2'-O-Methyladenosine	0.997	99.726	282.120	2.04E-04	7.37E-05	1.507	0.0000	0.0001	2.772	1.471
91	5-Methylcytidine	0.989	248.189	258.108	2.54E-06	6.61E-08	2.848	0.0274	0.0131	38.483	5.266
92	5'-Methylthioadenosine	0.975	93.396	298.096	2.29E-03	2.15E-04	2.306	0.0000	0.0001	10.644	3.412
93	Cytarabine	1.000	260.517	244.093	1.08E-04	3.45E-05	1.593	0.0029	0.0017	3.132	1.647
94	Cytidine	0.985	261.061	242.078	2.93E-05	6.96E-06	1.912	0.0001	0.0023	4.214	2.075
95	Deoxyinosine	0.868	197.586	251.078	4.56E-05	2.62E-05	1.145	0.0084	0.0206	1.742	0.801
96	Guanosine	0.995	285.853	284.099	2.33E-04	1.27E-04	1.151	0.0005	0.0003	1.828	0.871
97	Guanosine monophosphate	0.958	479.913	364.065	9.90E-06	4.67E-06	1.263	0.0090	0.0048	2.119	1.083
98	Inosine	0.999	237.500	269.087	6.83E-06	2.43E-06	1.491	0.0139	0.0071	2.816	1.493
99	Pseudouridine	0.895	263.589	243.062	1.76E-05	9.46E-06	1.234	0.0238	0.0287	1.866	0.900
100	Ribitol	0.854	248.984	151.060	3.32E-04	1.91E-04	1.114	0.0360	0.0309	1.743	0.802
101	Dihydrouracil	0.831	60.773	115.050	1.28E-04	6.83E-05	1.183	0.0001	0.0001	1.872	0.904
102	FAPy-adenine	0.951	188.880	154.072	1.71E-04	8.01E-05	1.307	0.0000	0.0001	2.138	1.097
103	Guanine	0.998	285.853	152.057	4.09E-04	2.15E-04	1.193	0.0002	0.0001	1.900	0.926
104	Thymidine	0.989	93.249	241.082	4.42E-05	2.79E-05	1.022	0.0148	0.0254	1.584	0.664
105	Citicoline	0.849	462.516	489.115	5.85E-05	1.89E-05	1.593	0.0000	0.0001	3.090	1.627

106	(±)-Conen	0.629	429.360	305.076	3.04E-05	1.59E-05	1.170	0.0206	0.0101	1.910	0.933
107	1-(beta-D-Ribofuranosyl)-1,4-dihydronicotinamide	0.590	176.033	257.113	3.21E-05	1.05E-05	1.549	0.0118	0.0061	3.064	1.615
108	1,3-Dihydro-(2H)-indol-2-one	0.994	249.072	134.060	1.94E-05	9.84E-06	1.173	0.0139	0.0071	1.969	0.977
109	1-Pyrroline	0.999	331.963	70.066	5.85E-06	2.45E-06	1.375	0.0025	0.0014	2.388	1.256
110	2-Hydroxypyridine	0.999	52.794	96.045	3.41E-04	1.38E-04	1.408	0.0015	0.0009	2.472	1.306
111	5-(2-Furanyl)-3,4-dihydro-2H-pyrrole	0.746	103.907	136.076	9.15E-05	5.70E-05	1.023	0.0015	0.0009	1.607	0.684
112	5-(2-Hydroxyethyl)-4-methylthiazole	0.985	48.170	144.048	8.53E-05	4.24E-05	1.173	0.0182	0.0091	2.012	1.009
113	9-(beta-D-Ribofuranosyl)zeatin	0.762	136.573	352.160	1.23E-05	6.61E-08	3.429	0.0046	0.0025	186.502	7.543
114	Benzofuran	0.536	325.574	119.049	3.10E-05	1.72E-06	2.996	0.0031	0.0017	17.952	4.166
115	Bisacurone epoxide	0.697	57.045	269.174	8.93E-06	2.90E-06	1.531	0.0195	0.0096	3.084	1.625
116	Diferuloylputrescine	0.808	56.704	441.201	3.12E-06	2.12E-07	2.514	0.0237	0.0115	14.734	3.881
117	Herierin IV	0.391	180.397	171.065	2.80E-05	1.30E-05	1.301	0.0003	0.0002	2.149	1.103
118	Isobutyrylglycine	0.989	231.568	144.065	2.87E-06	9.58E-08	2.952	0.0141	0.0250	30.004	4.907
119	Niacinamide	0.995	60.806	123.055	8.95E-03	4.82E-03	1.164	0.0006	0.0003	1.854	0.891
120	Sulfamethoxazole N4-hydroxylamine	0.898	83.156	270.052	1.38E-05	4.87E-06	1.483	0.0047	0.0026	2.834	1.503
121	Imidazole-4-acetaldehyde	0.751	63.761	111.055	2.78E-03	1.65E-03	1.072	0.0017	0.0010	1.688	0.755
122	Vanillin 3-(L-menthoxy)propane-1,2-diol acetal	0.626	50.251	365.231	2.62E-06	6.14E-07	1.786	0.0296	0.0140	4.273	2.095
123	3-Isoxazolidinone	0.999	397.408	88.040	4.75E-05	2.30E-06	2.712	0.0000	0.0001	20.627	4.366
124	Hypoxanthine	1.000	236.753	137.046	5.27E-05	1.78E-05	1.824	0.0242	0.0117	2.965	1.568
125	Imidazoleacetic acid	0.894	95.257	125.034	1.16E-04	7.47E-05	1.060	0.0009	0.0063	1.558	0.640
126	Xanthine	0.965	233.541	151.025	1.18E-04	3.21E-06	3.053	0.0000	0.0003	36.834	5.203
127	(R)-mandelic Acid	0.482	67.757	135.044	1.14E-05	3.59E-06	1.601	0.0109	0.0057	3.185	1.671
128	3-(4-hydroxy-3-methoxyphenyl)prop-2-enoic acid	0.851	167.700	193.050	1.83E-04	8.83E-05	1.367	0.0002	0.0028	2.076	1.054
129	Benzoic acid	0.996	326.112	123.044	8.62E-05	3.74E-05	1.366	0.0000	0.0001	2.303	1.204
130	Homovanillic acid	0.881	51.639	181.050	1.31E-03	4.24E-04	1.698	0.0003	0.0038	3.103	1.634
131	Isohomovanillic acid	0.500	37.922	181.050	2.04E-04	5.93E-05	1.785	0.0000	0.0003	3.442	1.783
132	Oxoglutaric acid	0.989	388.681	145.013	2.97E-04	7.97E-05	1.817	0.0005	0.0049	3.728	1.898
133	Phenylpyruvic acid	0.937	186.597	163.039	8.45E-04	4.10E-04	1.364	0.0000	0.0011	2.061	1.043
134	Pyrrolidonecarboxylic acid	1.000	320.278	128.034	2.71E-03	1.62E-03	1.138	0.0003	0.0037	1.667	0.737
135	Perilloside C	0.794	441.392	317.193	1.06E-05	4.94E-06	1.304	0.0000	0.0001	2.139	1.097
136	2-Pyrrolidinone	0.912	394.397	86.061	1.52E-03	4.89E-04	1.592	0.0000	0.0001	3.097	1.631
137	4-Pyridoxic acid	0.893	47.427	184.060	7.52E-05	4.04E-05	1.152	0.0061	0.0033	1.862	0.897
138	Pyridoxine	0.966	101.418	168.066	1.72E-04	1.02E-04	1.145	0.0010	0.0067	1.685	0.753
139	4-Dodecylbenzenesulfonic Acid	0.979	28.382	325.184	6.30E-03	3.20E-03	1.310	0.0012	0.0073	1.973	0.980
140	Ketoleucine	0.993	58.181	129.055	1.85E-02	1.20E-02	1.048	0.0005	0.0047	1.544	0.627
141	Methyl acrylate	0.991	395.225	87.045	1.40E-03	4.80E-04	1.555	0.0000	0.0001	2.928	1.550

142	O-Phosphoethanolamine	0.921	488.856	142.026	3.11E-05	1.41E-05	1.309	0.0026	0.0015	2.198	1.136
143	Dihydrolipoate	0.540	67.353	207.050	3.78E-04	2.16E-04	1.172	0.0057	0.0171	1.756	0.812
144	1-deoxy-1-(N6-lysino)-D-fructose	0.759	424.806	132.029	3.11E-04	7.49E-04	1.506	0.0000	0.0015	0.415	-1.270
145	D-Aspartic acid	0.996	424.735	134.045	6.97E-05	2.59E-04	1.716	0.0000	0.0001	0.269	-1.893
146	L-Pipecolic acid	0.993	224.347	130.083	3.43E-05	7.07E-05	1.269	0.0002	0.0001	0.485	-1.043
147	gamma-Glutamylvaline	0.607	396.464	247.129	1.04E-04	1.65E-04	1.013	0.0004	0.0002	0.631	-0.665
148	Histidinyl-Asparagine	0.716	394.785	270.119	3.75E-06	8.09E-06	1.249	0.0208	0.0102	0.464	-1.107
149	Lysyl-Methionine	0.580	403.764	278.153	2.41E-06	7.25E-06	1.559	0.0184	0.0091	0.332	-1.590
150	N-a-Acetyl-L-arginine	0.853	392.121	217.129	2.77E-04	4.58E-04	1.050	0.0010	0.0006	0.605	-0.724
151	Serylasparagine	0.491	377.862	220.105	4.55E-05	8.65E-05	1.166	0.0063	0.0034	0.526	-0.927
152	Tryptophyl-Tryptophan	0.550	211.644	391.175	1.78E-05	3.36E-05	1.163	0.0067	0.0036	0.530	-0.916
153	Trehalose 6-phosphate	0.627	497.112	421.075	6.20E-05	1.03E-04	1.121	0.0032	0.0123	0.602	-0.732
154	16-Methylheptadecanoic acid	1.000	50.084	283.264	4.00E-04	8.17E-04	1.229	0.0482	0.0322	0.489	-1.032
155	6,15-Diketo,13,14-dihydro-PGF1a	0.702	477.341	369.224	1.92E-05	3.38E-05	1.102	0.0048	0.0027	0.568	-0.815
156	9,10-Epoxyoctadecenoic acid	0.991	54.675	295.227	3.09E-04	8.21E-04	1.584	0.0002	0.0029	0.376	-1.411
157	DG(22:6(4Z,7Z,10Z,13Z,16Z,19Z)/14:0/0:0)	0.734	32.334	613.482	1.43E-05	4.62E-05	1.519	0.0392	0.0181	0.309	-1.695
158	Linoleoyl ethanolamide	0.856	35.904	324.289	1.90E-05	8.10E-05	1.789	0.0031	0.0017	0.235	-2.089
159	LysoPC(14:0/0:0)	0.537	228.128	468.308	8.16E-06	3.07E-05	1.699	0.0057	0.0031	0.265	-1.914
160	LysoPC(16:1(9Z)/0:0)	0.528	225.425	494.325	1.12E-05	3.37E-05	1.489	0.0270	0.0130	0.332	-1.592
161	LysoPC(18:2(9Z,12Z))	0.630	255.392	520.335	5.20E-08	9.24E-07	2.506	0.0455	0.0206	0.056	-4.151
162	LysoPC(18:3(6Z,9Z,12Z))	0.534	225.366	518.324	3.89E-04	8.27E-04	1.235	0.0206	0.0101	0.471	-1.087
163	LysoPE(16:0/0:0)	0.964	229.741	454.293	1.32E-04	4.26E-04	1.551	0.0237	0.0115	0.309	-1.695
164	LysoPE(18:1(9Z)/0:0)	0.717	226.994	480.309	3.32E-05	9.92E-05	1.449	0.0404	0.0186	0.335	-1.580
165	LysoPE(20:5(5Z,8Z,11Z,14Z,17Z)/0:0)	0.850	228.739	500.275	4.00E-06	8.70E-06	1.247	0.0310	0.0147	0.460	-1.120
166	Myristic acid	1.000	51.084	227.201	1.25E-04	2.07E-04	1.051	0.0362	0.0310	0.606	-0.723
167	PS(18:0/22:6(4Z,7Z,10Z,13Z,16Z,19Z))	0.815	178.600	836.541	7.61E-07	2.40E-05	2.789	0.0228	0.0111	0.032	-4.980
168	Ricinoleic acid	0.999	55.227	297.242	1.39E-04	2.45E-04	1.178	0.0072	0.0193	0.567	-0.820
169	10E,12Z-Octadecadienoic acid	1.000	50.022	279.232	2.30E-03	6.65E-03	1.620	0.0064	0.0182	0.345	-1.534
170	13-OxoODE	0.989	38.051	293.212	9.99E-05	3.10E-04	1.694	0.0003	0.0034	0.322	-1.633
171	Alpha-Linolenic acid	1.000	50.315	277.217	4.52E-04	1.49E-03	1.727	0.0037	0.0134	0.304	-1.717
172	1-Palmitoylglycerophosphoinositol	0.884	273.602	573.302	3.02E-05	9.54E-05	1.548	0.0168	0.0084	0.317	-1.657
173	LysoPE(18:3(6Z,9Z,12Z)/0:0)	0.922	230.628	476.277	4.04E-05	9.40E-05	1.364	0.0013	0.0008	0.430	-1.218
174	Calendulaglycoside E	0.453	262.060	795.450	4.21E-05	9.48E-05	1.335	0.0010	0.0006	0.445	-1.170
175	11-Methylgerberinol	0.998	405.415	379.121	2.35E-04	4.10E-04	1.112	0.0081	0.0043	0.574	-0.802
176	Solavetivone	0.925	33.335	219.174	9.41E-07	2.32E-06	1.362	0.0125	0.0064	0.406	-1.300
177	Diacetone alcohol	0.980	72.839	115.075	7.96E-08	2.62E-04	4.572	0.0009	0.0064	0.000	-11.687
178	5-Aminoimidazole ribonucleotide	0.498	407.904	296.066	1.14E-04	2.99E-04	1.462	0.0088	0.0047	0.382	-1.387
179	Adenine	0.999	174.905	134.046	1.82E-03	2.76E-03	1.022	0.0014	0.0080	0.661	-0.598

180	3,11,12-Trihydroxy-1(10)-spirovetiven-2-one	0.561	37.691	269.174	4.21E-06	9.40E-06	1.337	0.0005	0.0003	0.448	-1.159
181	Beta-Carboline	0.918	50.130	169.076	2.26E-05	3.67E-05	1.038	0.0003	0.0002	0.615	-0.702
182	Dinoseb acetate	0.901	397.418	283.090	6.08E-07	2.81E-06	1.884	0.0023	0.0013	0.216	-2.210
183	Ethylbenzene	0.931	34.995	107.086	1.61E-05	2.78E-05	1.084	0.0048	0.0027	0.579	-0.788
184	Gymnodimine	0.476	223.867	508.339	4.03E-06	1.07E-05	1.395	0.0299	0.0142	0.375	-1.414
185	Phosphoribosyl formamidocarboxamide	0.595	418.300	367.062	1.22E-04	2.17E-04	1.122	0.0026	0.0015	0.561	-0.833
186	Testosterone	0.885	33.281	289.216	1.11E-06	3.97E-06	1.630	0.0253	0.0122	0.280	-1.835
187	Ganoderal A	0.720	34.374	437.341	4.55E-07	7.91E-06	2.592	0.0007	0.0004	0.057	-4.121
188	Ganoderic acid Mi	0.761	33.248	545.384	8.07E-07	3.69E-06	1.837	0.0011	0.0007	0.219	-2.192
189	Pisumsaponin II	0.470	300.376	941.511	7.65E-05	1.53E-04	1.236	0.0005	0.0003	0.502	-0.996
190	S-Furanopetasitin	0.795	495.262	433.203	4.80E-07	2.47E-06	1.972	0.0156	0.0078	0.194	-2.367
191	6-[2,3-Dihydroxy-1-(hydroxymethyl)propyl]-1,2-dihydro-7-hydroxy-9-methoxy-cyclopenta[c][1]benzopyran-3,4-dione	0.894	409.850	351.104	3.83E-05	8.03E-05	1.270	0.0205	0.0101	0.477	-1.068
192	Fumaric acid	0.998	247.122	115.002	4.32E-04	7.73E-04	1.142	0.0313	0.0302	0.560	-0.838
193	Erythrono-1,4-lactone	1.00	480.90	117.02	0.00	0.00	1.09	0.01	0.02	0.62	-0.693

Note: rt is the chromatographic retention time of the substance; Mass is the exact molecular weight of the metabolite; score is the match with the substance in the database; The MEAN p is the relative quantitative mean of the substances in the cooked black bean group; The MEAN ops is the relative quantitative mean of the substances in the steamed black bean group; VIP is the variable projection importance obtained by the OPLS-DA model of the comparison in this group; P-VALUE is the P value obtained by the t-test of the comparison in this group, P value = the probability that the hypothesis is correct but rejected = the number of negative results / the total number of results, which is a test probability for the sample data; Q-VALUE is the hypothesis test statistic (P value) after correction for multiple hypothesis testing The result of , Q value = probability of being rejected but correct = number of false positive results / number of presumed positive results, which is a test probability for the inference obtained by the t test, and a re-statistic for the P value; FOLD CHANGE is the fold relationship of the substance in the comparison between the two groups of experiments; LOG_FOLDCHANGE FOLD CHANGE is the logarithm with the base 2, "-" means the relative content is low.
